# 2dFDR: a new approach to confounder adjustment substantially increases detection power in omics association studies

**DOI:** 10.1186/s13059-021-02418-8

**Published:** 2021-07-13

**Authors:** Sangyoon Yi, Xianyang Zhang, Lu Yang, Jinyan Huang, Yuanhang Liu, Chen Wang, Daniel J. Schaid, Jun Chen

**Affiliations:** 1 Department of Statistics, Texas A&M University, College Station, TX 77843 USA; 2 Division of Computational Biology, Department of Quantitative Health Sciences, Mayo Clinic, Rochester, MN 55905 USA; 3 State Key Laboratory of Medical Genomics, Shanghai Institute of Hematology, National Research Center for Translational Medicine, Rui-Jin Hospital, Shanghai Jiao Tong University, Shanghai, 200025 China

**Keywords:** Confounding, False discovery rate, Association testing

## Abstract

One challenge facing omics association studies is the loss of statistical power when adjusting for confounders and multiple testing. The traditional statistical procedure involves fitting a confounder-adjusted regression model for each omics feature, followed by multiple testing correction. Here we show that the traditional procedure is not optimal and present a new approach, 2dFDR, a two-dimensional false discovery rate control procedure, for powerful confounder adjustment in multiple testing. Through extensive evaluation, we demonstrate that 2dFDR is more powerful than the traditional procedure, and in the presence of strong confounding and weak signals, the power improvement could be more than 100%.

**Supplementary Information:**

The online version contains supplementary material available at 10.1186/s13059-021-02418-8.

## Background

High-throughput genomic profiling technologies enable the interrogation of the biological system at different omics levels, generating enormous amounts of omics data [1]. One central task of statistical analysis of omics data is to test the association between omics features and a covariate of interest [2]. The associated omics features, once validated, can provide biological insights into health and disease, act as potential targets for intervention and serve as biomarkers for clinical applications [3, 4]. However, observational omics studies are subject to various types of confounding [5–7]. Confounding arises when the relationship between the primary variable and the omics feature is distorted by some other variable (confounder) due to its association with both. Demographic variables like age, gender, race, and obesity are frequent confounders in omics association studies. For example, in cancer studies, cancer patients are often older than benign controls [8]. In other diseases such as rheumatoid arthritis, the prevalence could differ by gender [9]. Since these demographic variables are known to impact the omics profiles [7], their correlation with the variable of interest could confound the association analysis. Biological heterogeneity, such as the cell mixture in the tissue sample, is also a significant source of confounding. This is because the cell types have distinct omics profiles, and their composition could vary with the variable of interest. For example, the leukocyte composition in the peripheral blood often shifts in the disease condition, and therefore it could severely confound blood-based omics studies [5]. Finally, technical variation, or batch effect, which occurs when the biological samples are not processed or measured together, could strongly confound the associations of interest if the samples are not randomized into the batches [6]. Although the batch confounding can be avoided by careful study design, it is unfortunately prevalent in omics studies [6].

Confounding not only reduces the statistical power by introducing extra variability but also increases the chance of false findings if not properly accounted for. Standard statistical approaches to address confounding include stratification and regression, with the latter being most widely used due to its flexibility [10]. Although adjusting the confounders in the regression model controls false positives, it nevertheless reduces the statistical power. The need for multiple testing correction in omics association analysis further deteriorates statistical power [11]. In the presence of strong confounding, it is not unusual that no significant associations could be recovered after adjusting for confounders and multiple testing. Therefore, improving the power under confounding and multiple testing is a topic of critical importance and could potentially rescue an underpowered study.

Previous methods separate confounder adjustment from multiple testing. The standard approach, which fits a confounder-adjusted regression model for all omics features followed by multiple testing correction such as false discovery rate (FDR) control [12, 13], is used predominantly [14]. However, confounders may affect only a subset of omics features [15–18], and adjusting confounders for every omics feature will be an over-adjustment, leading to substantial power loss. To rescue the power, one naïve idea is to test the significance of the confounder first, and if it is not significant, we exclude the confounder in the regression model. Although this strategy substantially improves the power, controlling the type I error is difficult and heavily depends on the choice of the significance cutoff. We found that this strategy fails to control the type I error properly, even if we use a very lenient cutoff.

In this study, we take a different approach to this problem and integrate the confounder adjustment into multiple testing (FDR control) framework. The new approach uses the statistic from the unadjusted analysis to filter out omics features that are less likely to be associated with the covariate of interest or the confounder. FDR control is then performed based on the adjusted statistic on the remaining features. The challenge here is to account for the dependency between the unadjusted and adjusted statistic so that the FDR is controlled. We provide a robust and powerful procedure, two-dimensional false discovery rate control procedure (2dFDR), which is proved to offer asymptotic FDR control and dominate the power of the traditional procedure.

## Results

### Overview of the two-dimensional false discovery rate control procedure (2dFDR)

**Fig. 1:**
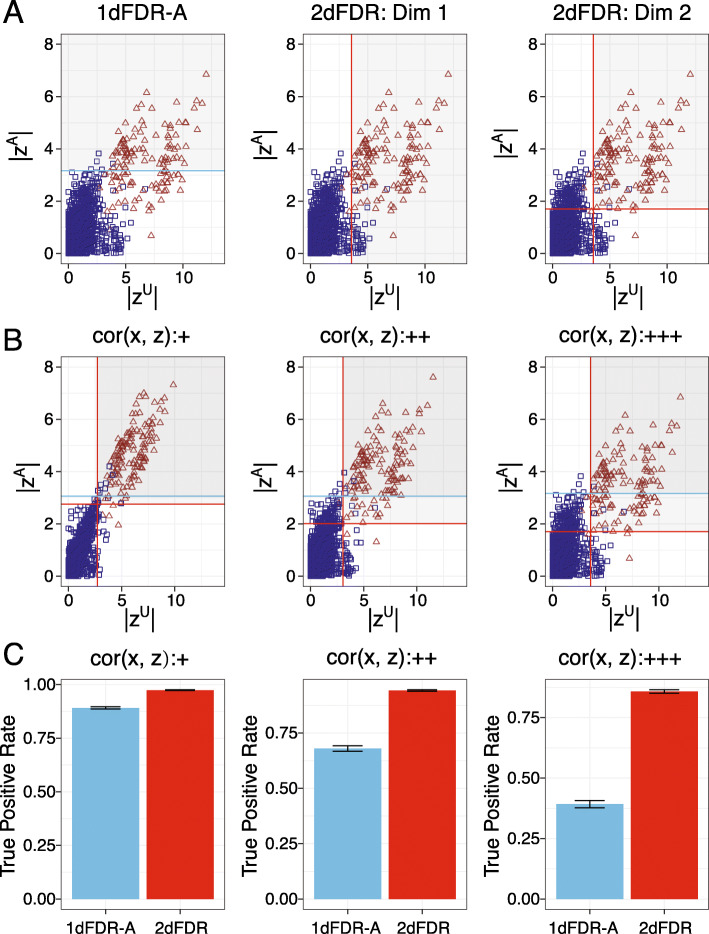
Illustration of the 2dFDR procedure using simulated datasets. **A** The decision boundaries for 1dFDR-A (blue line) and 2dFDR (red line) at 5% FDR for a highly confounded scenario (ρ ≈ 0.8). 1dFDR-A relies on adjusted statistic |*Z*^A^| only (one dimension), while 2dFDR is based on both the adjusted and unadjusted statistic |*Z*^A^| and |*Z*^U^| (two dimensions). |*Z*^U^| is used to exclude a large number of irrelevant features (red vertical line). After that, a less stringent cutoff of |*Z*^A^| (red horizontal line) can be used to achieve a higher power while maintaining the same FDR. The aim of 2dFDR is thus to find the best cutoffs on the two dimensions to maximize the power. **B, C** The power (true positive rate) difference between 1dFDR-A and 2dFDR increases with the correlation between the variable of interest and the confounder. When the correlation is low (“+,” ρ ≈ 0.2), |*Z*^A^| and |*Z*^U^| are highly correlated, and |*Z*^U^| provides little extra information. The power of 2dFDR is thus similar to that of 1dFDR-A. When the correlation is higher (“++,” “+++,” ρ ≈ 0.6, 0.8), the signals (brown) and noises (blue) are more difficult to separate on |*Z*^A^|. By using |*Z*^U^|, 2dFDR excludes a large number of noises without losing many signals. The signal density on |*Z*^A^| is thus enriched, leading to a significant power gain

2dFDR is based on linear models with the measurement of the omics feature as the outcome, which is one popular modeling approach for functional omics data, and assumes the confounders are known. It depends on the unadjusted and adjusted test statistics, denoted by ${Z}_i^U$ and ${Z}_i^A$, respectively, from fitting both the unadjusted and the confounder-adjusted model to the *i*th omics feature. 2dFDR proceeds in two dimensions. In the first dimension, it uses the unadjusted statistic ${Z}_i^U$ to screen out a large number of irrelevant features (noises) that are not associated with the covariate of interest or the confounder. In the second dimension, it uses the adjusted statistic ${Z}_i^A$ to identify the true signals on the remaining features and control the FDR at the desired level. Although the unadjusted statistic is biased and captures the effects from both the covariate of interest and the confounder, it can be leveraged to increase the signal density and reduce multiple testing burden in the second dimension. Thus, 2dFDR boils down to selecting features with $\mid {Z}_i^U\mid \ge {t}_1$ (first dimension) and $\mid {Z}_i^A\mid \ge {t}_2$ (second dimension). The cutoffs *t*_1_ and *t*_2_ are chosen to achieve maximum power while controlling the FDR at the desired level. Figure 1A–C illustrate the idea using simulated data (Additional file 1: Note S1), where we plot ${Z}_i^A$ against ${Z}_i^U$ for confounded scenarios. The standard approach performs (one-dimensional) FDR control based on the adjusted statistic ${Z}_i^A$ only (we refer it as 1dFDR-A). When there the correlation between the variable of interest and the confounder (denoted as “cor(x, z)”) is high, the signals (brown) and noises (blue) overlap much on ${Z}_i^A$ due to loss of power with confounder adjustment (Fig. 1A). To achieve the desired FDR level, 1dFDR-A requires a high $\mid {Z}_i^A\mid$ cutoff (blue line). For 2dFDR, it first uses ${Z}_i^U$ to exclude a large number of irrelevant features (vertical red line). Next, a much lower $\mid {Z}_i^A\mid$ cutoff (horizontal red line) is used to achieve the same FDR level. As a result, it achieves significant power improvement, and the improvement increases with the correlation between the variable of interest and the confounder (Fig. 1B, C).

A particular challenge for this new approach is to address the dependency between the two dimensions. 2dFDR simultaneously selects *t*_1_ and *t*_2_ and considers the selection effect in the first dimension (“Methods” and Additional file 1: Note S2). 2dFDR can be viewed as a two-dimensional generalization of the classical Benjamini-Hochberg (BH) procedure [12], where we search for the cutoff values in a two-dimensional space. An intrinsic difficulty is to estimate the expected number of false rejections at a given *t*_1_ and *t*_2_; this is achieved by a non-parametric Empirical Bayes method [19] (Additional file 1: Note S2.3). We have conducted a thorough theoretical investigation of the proposed procedure and all the theoretical results are included in Additional file 1: Note S3 and S4. Under suitable assumptions, we show that 2dFDR provides asymptotic FDR control (Additional file 1: Note S3), and the power dominates the standard 1dFDR-A (Additional file 1: Note S4).

### Simulation studies to evaluate FDR control and power

We demonstrate the power and robustness of 2dFDR using comprehensive simulations comparing to 1dFDR-U and 1dFDR-A, two one-dimensional FDR procedures based on the unadjusted and adjusted model, respectively. A heuristic strategy (1dFDR-H), which starts with the adjusted model and uses the unadjusted model if the effect of the confounder is not significant, was also compared. We refer the omics features affected by the variable of interest as “true signals” and the omics features affected by the confounder as “confounding signals.” For both the true and confounding signals, we use “signal density” and “signal strength” to represent the percentage of features affected and their effect size, respectively.

**Fig. 2:**
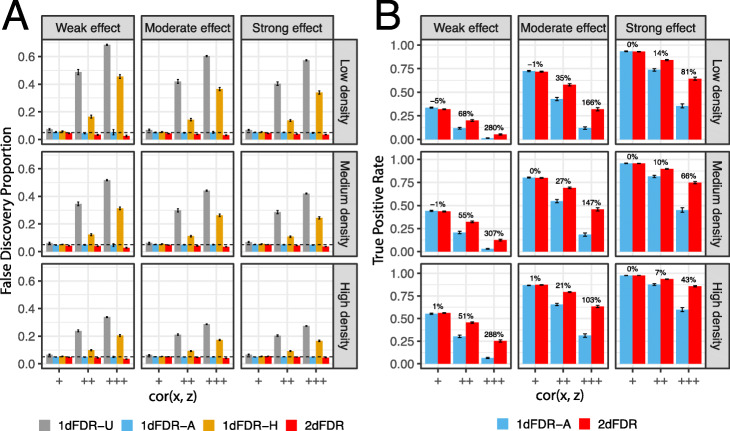
Performance on simulated datasets across varying density and strength of the *true signals*. Average false discovery proportions and true positive rates are compared at 5% FDR using simulated datasets. 1dFDR-U and 1dFDR-A represent the one-dimensional FDR control procedures based on the unadjusted model and confounder-adjusted model, respectively. 1dFDA-H is a heuristic adaptive procedure that uses the adjusted or unadjusted model depending on whether the confounder effect is significant (nominal *p* value < 0.05). The performance is evaluated at varying signal strength (left: weak, right: strong), signal density (top: low, bottom: high), and the correlation between the variable of interest and the confounder (inside the panel, “+,” “++,” and “+++” represent a low, medium, and high correlation (ρ ≈ 0.2, 0.6, 0.8), respectively). The density of the confounding signals is 10%, and the strength is moderate. 2dFDR and 1dFDR-A control the FDR at the target level (dashed line), while 1dFDR-U and 1dFDR-H fail to control the FDR properly when the confounding is not weak (**A**). 2dFDR becomes substantially more powerful than 1dFDR-A as the correlation between the variable of interest and the confounder increases (**B**). The difference is more pronounced when the signals are weak and sparse, as indicated by the percent increase shown on top of the bars

We first study the performance of 2dFDR under varying signal density, signal strength, and cor(x, z) (“Methods”). Both 1dFDR-A and 2dFDR controlled the FDR at the target level across settings, while 1dFDR-U and 1dFDR-H failed to control the FDR under a medium or high cor(x, z) (Fig. 2A). 2dFDR was substantially more powerful than 1dFDR-A when cor(x, z) was high and was comparable when cor(x, z) was low (Fig. 2B). The power increase was more pronounced for weak and sparse signals, with a percent increase of more than 100% (Fig. 2B). This is particularly relevant for real applications, where weak signals and strong confounding are the most challenging situation that needs novel methodological developments.

**Fig. 3:**
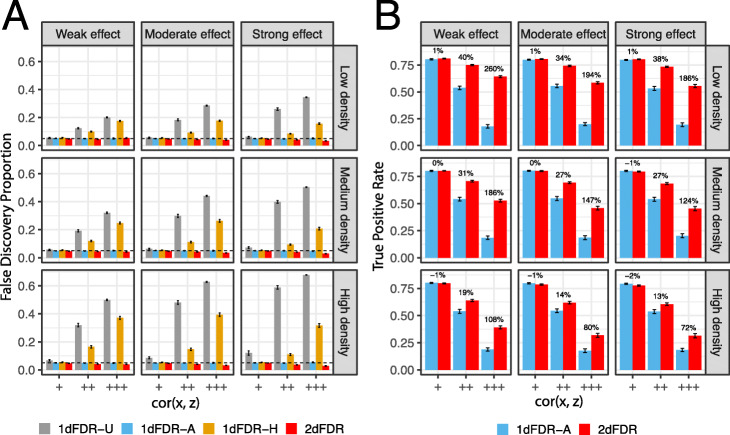
Performance on simulated datasets across varying density and strength of the *confounding signals.* Average false discovery proportions (**A**) and true positive rates (**B**) are compared at 5% FDR using simulated datasets. 1dFDR-U and 1dFDR-A represent the one-dimensional FDR control procedures based on the unadjusted model and confounder-adjusted model, respectively. 1dFDA-H is a heuristic adaptive procedure that uses the adjusted or unadjusted model depending on whether the confounder effect is significant (nominal *p* value < 0.05). The density of the true signals is 10%, and the strength is moderate. The performance is evaluated at varying confounding signal strength (left: weak, right: strong), confounding signal density (top: low, bottom: high), and the correlation between the variable of interest and the confounder (inside the panel, “+,” “++,” and “+++” represent a low, medium, and high correlation (ρ ≈ 0.2, 0.6, 0.8), respectively). 2dFDR maintains the FDR at the target level across settings and is significantly more powerful when the correlation between the variable of interest and the confounder is not low (++/+++). The power difference decreased as the confounding signals become denser (top to bottom)

We next study the effect of the confounding signals’ strength and density by varying their magnitudes (Fig. 3) while fixing the true signals’ strength and density. Similarly, 2dFDR maintained the FDR at the target level across settings (Fig. 3A) and was significantly more powerful when cor(x, z) was medium or high (Fig. 3B). The power difference, however, decreased as the confounding signals became denser (top to bottom). When the confounder affected 50% of the features, 2dFDR could be less powerful than 1dFDR-A even when cor(x, z) was high (Additional file 2: Figure S1). This is expected since if the confounder affects every omics feature, 1dFDR-A, which adjusts the confounder for every omics feature, is optimal. Higher strength of the confounding signals (left to right) also reduced the power difference. The results remained the same if we simulated five confounders (Additional file 2: Figure S2).

We also studied the effect of colocation between the true and confounding signals, where the omics features were affected by both the variable of interest and the confounder (Additional file 2: Figure S3). We found that 2dFDR was more powerful than 1dFDR-A when the density of the confounding signals was low and cor(x, z) was high. However, as the confounding signals became denser, 2dFDR could be less powerful than 1dFDR-A when cor(x, z) was low.

Since 2dFDR is developed based on the assumption that the omics features are independent, it is important to study the robustness of 2dFDR to the correlations among omics features. We thus simulated block and autoregressive correlation structures (“Methods”), which were commonly observed for omics data. We found that 2dFDR was quite robust to these two correlation structures (Additional file 2: Figures S4 and S5), and the FDR was controlled near the target level. 2dFDR maintained the power in these scenarios.

2dFDR offers asymptotic FDR control, i.e., the FDR is proved to be controlled if the sample size and feature size are large. It is interesting to study the sample size and feature size where it breaks down. We thus simulated sample sizes of 50 and 25 (Additional file 2: Figure S6) and feature sizes of 500 and 100 (Additional file 2: Figure S7). We found that the performance 2dFDR remained robust and powerful at the sample size of 50 and the feature size of 500. However, it became less powerful than the traditional procedure at the sample size of 25. The FDR also started to be inflated at the feature size of 100, especially when cor(x, z) was high. We also found that increasing the sample size or feature size alone did not rescue the performance deterioration due to the other being small (Additional file 2: Figure S8). Overall, 2dFDR is robust up to a moderate sample size and feature size. For a very small sample or feature size, applying 2dFDR is not recommended.

2dFDR is computationally efficient and can scale up to a large sample and feature size. It can be run in parallel on each grid point to further increase its computational speed. Additional file 2: Figure S9 shows the computational time for running a simulation instance at different sample sizes and feature sizes. With n = 800, m = 512k and one confounder, 2dFDR completes the analysis in 546 s using a search grid of 50 × 50 without parallelization on a MacBook Pro laptop. The memory requirement is the same as fitting regular linear regressions and requires only accommodating a matrix multiplication of A_p × n_ *B*_*n* × *m*_, where p, n, and m are the number of covariates, sample size, and feature size, respectively.

### Evaluation of the detection power on real omics datasets

We apply 2dFDR to three different types of omics datasets to demonstrate its empirical power on real data. We compare to the traditional adjusted procedure 1dFDR-A based on the numbers of detected omics features at the same FDR level.

**Fig. 4:**
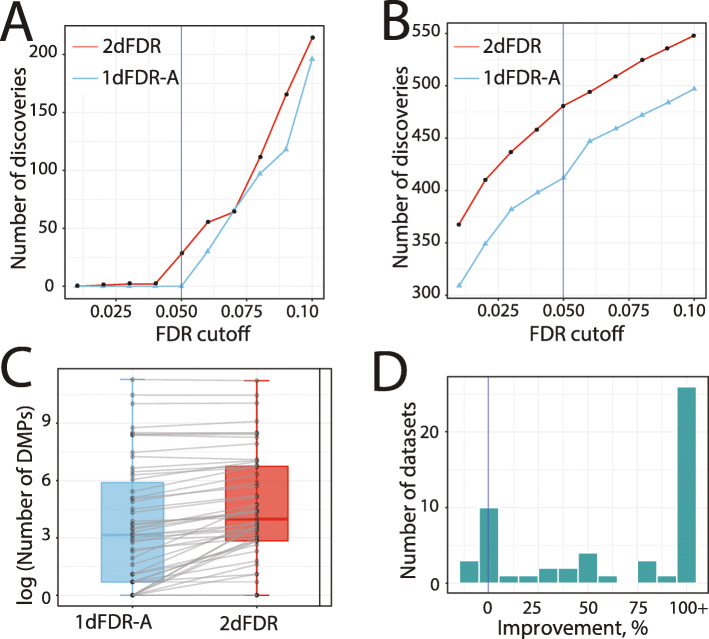
The empirical power of 2dFDR on real omics datasets. 2dFDR made more discoveries than 1dFDR-A across FDR target levels for **A** TCGA hepatocellular carcinoma transcriptomics (RNA-Seq) dataset (m = 19,329, confounder: gender and ethnicity), **B** insulin resistance metabolomics dataset pooling polar metabolites and molecular lipids (m = 1201, confounder: BMI). **C, D** Evaluation of 2dFDR on 54 epigenomics (450 K methylation array) datasets from EWAS of various phenotypes (m ≅ 450,000, confounder: cell mixtures). **C** Boxplot comparing the number of DMPs (differentially methylated positions) detected by 2dFDR and 1dFDR-A at 5% FDR over the 54 datasets. 2dFDR recovered more DMPs than 1dFDR-A in 43 datasets. Each gray dot represents a dataset, and the same dataset is connected by a line. **D** The distribution of the percent increase in detection power over the 54 datasets. 2dFDR achieves a median percent increase of 136% over 1dFDR-A

The first is a hepatocellular carcinoma transcriptomics dataset from TCGA [20] (n = 342, m = 19,329), which is used to detect gene expressions associated with human hepatitis B virus (HBV) infection [21]. Gender and ethnicity are confounders for this dataset and were adjusted in the model. 2dFDR detected more genes than 1dFDR-A across different FDR levels (Fig. 4A). At the standard 5% FDR level, 1dFDR-A failed to identify any HBV-associated genes, while 2dFDR successfully identified 27 genes.

The second is a metabolomics dataset [22, 23] (n = 289, m = 1,201), where the aim is to identify serum metabolites associated with insulin resistance (IR), accounting for the confounding effect of body mass index (BMI). Again, 2dFDR detected more IR-associated metabolites at different FDR levels (Fig. 4B). At 5% FDR, 2dFDR and 1dFDR-A recovered 481 and 412 metabolites, respectively. 2dFDR was able to identify the majority of the metabolites by 1dFDR-A (378 out of 412) and it also recovered 103 metabolites missed by 1dFDR-A.

Finally, we benchmark 2dFDR using an extensive collection of epigenomics datasets from various epigenome-wide association studies (EWAS) using tissue samples [24] (Additional file 2: Table S1). The objective is to identify differentially methylated CpG positions (DMPs) associated with a condition of interest. Since a tissue sample contains a mixture of cell types, each with a distinct methylation profile, the covariation of their mixture proportion with the condition could strongly confound the associations of interest [5]. To capture the cell mixture, we used surrogate variable analysis (SVA), and the estimated surrogate variables were adjusted in the model [25]. For these EWAS datasets, 2dFDR detected significantly more DMPs than 1dFDR-A in most datasets with a median increase of 136% (Fig. 4C, D, Additional file 2: Table S1). Consistent with the simulations, the power improvement was more pronounced when the signals were weak (lower part of the box plot in Fig. 4C). Moreover, 2dFDR was able to detect DMPs in six datasets, where 1dFDR-A failed to identify any.

### Validation of the increased detection power on EWAS datasets

**Fig. 5:**
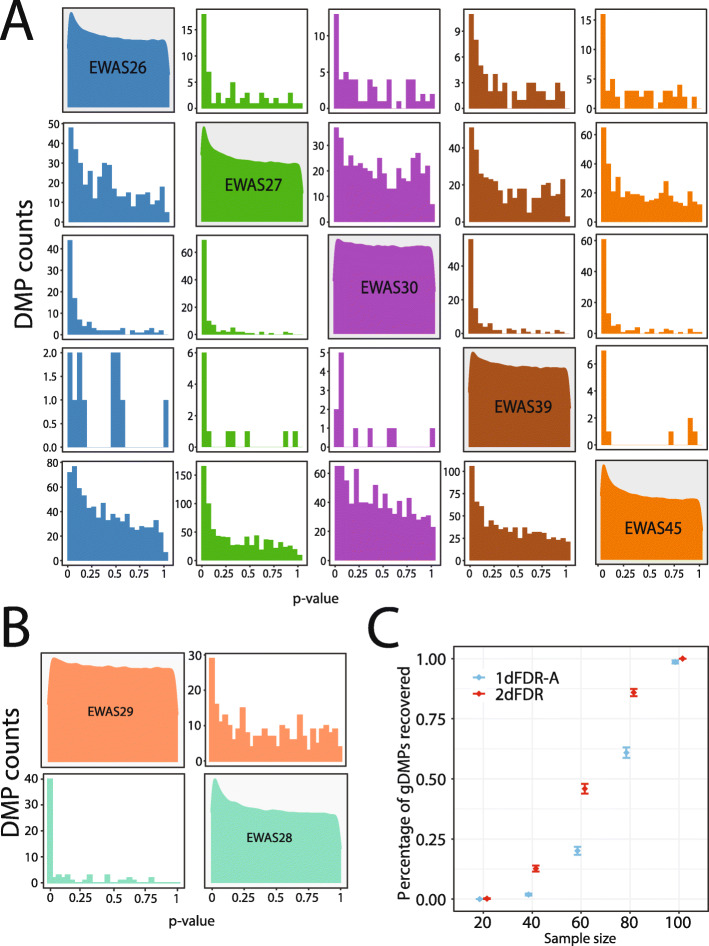
Validation of 2dFDR using EWAS datasets. **A** The distribution of the confounder-adjusted *p* values for those 2dFDR-exclusive DMPs in other age EWAS datasets. The diagonal parts show the densities of *p* values of all loci for the five age EWAS datasets, serving as a baseline. The off-diagonal parts show the distribution of *p* values of 2dFDR-exclusive DMPs in other age datasets. For instance, the first row shows the distribution of *p* values for the 2dFDR-exclusive DMPs from EWAS26 in EWAS27 (green), EWAS30 (purple), EWAS39 (brown), and EWAS45 (orange). The distribution is enriched in smaller *p* values than the distribution of all *p* values for the respective dataset (diagonal). **B** Similar validation using two systemic lupus erythematosus (SLE) EWAS datasets (EWAS28 and EWAS29, Additional file 2: Table S1). **C** Validation using a downsampling strategy on EWAS22 (*n* = 111). First, a list of “gold standard” DMPs (gDMPs) was created using Bonferroni correction based on the *p* values from the adjusted analysis on the full dataset. Next, the full dataset was downsampled to smaller sample sizes, and the ability of 2dFDR and 1dFDR-A in recovering those gDMPs was compared. For each sample size, 100 replications were performed, and means and standard errors are plotted. 2dFDR is more powerful in identifying these gDMPs at smaller sample sizes

To validate the additional DMPs detected by 2dFDR, we resorted to the five age-related EWAS datasets (Additional file 2: Table S1) to see if the additional DMPs from one age dataset had evidence of support from the other four. This was achieved by examining the confounder-adjusted *p* value distribution of the DMPs detected by 2dFDR only (at 5% FDR) in the other four age datasets. If these DMPs from one age dataset were truly age-associated, we expect to see smaller *p* values for them in the other age datasets, compared to the *p* values of random CpG loci. Clearly, the distribution was enriched in small *p* values, indicating the plausibility of DMPs detected by 2dFDR (Fig. 5A). Validation based on the two SLE datasets reached a similar conclusion (Fig. 5B).

We further performed a downsampling analysis to validate the improved power of 2dFDR. We first curate a list of highly significant features using Bonferroni correction based on the *p* values from the adjusted analysis on the full dataset. Next, we downsample the full dataset to smaller sizes and compare the ability of 2dFDR and 1dFDR-A in recovering these highly significant features as a way of power assessment. We illustrate this strategy using one EWAS dataset (EWAS22, *n* = 111), where the genome-wide methylation difference was compared between smokers and non-smokers in African American women using peripheral blood mononuclear cells [26]. With Bonferroni correction (alpha = 0.05) to the *p* values based on the full dataset, we identified 10 differentially methylated CpG positions (DMPs) and these DMPs were treated as “gold standard” (we label them as “gDMPs”). We then subsampled the full dataset to sample sizes of 20, 40, 60, 80, and 100, and compared the power (recovery rate) of 2dFDR and 1dFDR-A in recovering these gDMPs. We observed that 2dFDR outperformed 1dFDR-A at nearly all sample sizes (Fig. 5C). The power improvement was more significant in the middle range (i.e., *n* = 60 and 80). At the sample size of 100, the recovery rates of both 2dFDR and 1dFDR-A both reached nearly 100%.

## Discussion

Confounding and high-dimensionality are the two major statistical challenges in omics data analysis. Previous research separates these two problems, and methodological developments are focused on each of them. In this study, we integrate the confounder adjustment into multiple testing by performing two-dimensional false discovery rate control based on both the adjusted and unadjusted statistics. Although the unadjusted statistic is biased, it can be leveraged to enrich signals and reduce the multiple testing burden. The resulting procedure, 2dFDR, has proven to offer asymptotic FDR control and dominate the power of the traditional procedure based on the adjusted statistic only. Through simulations and real data applications, we demonstrate that 2dFDR is substantially more powerful than the traditional procedure. We also show that 2dFDR is robust to the typical correlation structures seen in omics data and performs well at moderate sample sizes and feature sizes.

The 2dFDR procedure is the most powerful when the correlation between the variable of interest and the confounder is high, and/or the signals are weak. This makes it a practically very useful approach since existing methods have limited power in these scenarios. 2dFDR also works best when the confounder only affects a subset of omics features. This is usually a reasonable assumption for many conditions, such as age and gender [15–18]. However, there could be situations where the assumption is violated. For example, strong batch effects could possibly affect a large number of omics features. In such a case, 2dFDR has a limited power advantage or could be less powerful than the traditional procedure (Additional file 2: Figure S1). One diagnostic approach is to calculate the percentage of the genomic variance explained (R^2^) by the variable of interest and the confounder, respectively, using multivariate methods such as PERMANOVA [27]. If the R^2^ of the confounder is substantially larger than that of the variable of interest, it indicates that the confounder signals may be very dense. Another approach is to study the distribution of the *p* values of the confounder from the adjusted analysis. If we see a spike on the left end of the *p* value distribution, it also suggests dense confounder signals.

Since 2dFDR depends on the unadjusted statistic to filter features, when the confounder and variable of interest have opposite effects on the omics feature with similar magnitude, they will cancel out each other’s effect, and the omics feature could be excluded erroneously in the first dimension. The optimal cutoff of $\mid {Z}_i^U\mid$ is thus determined based on the tradeoff between power reduction due to erroneously excluding these relevant features in the first dimension and power increase due to reducing the multiple testing burden and increasing the signal density in the second dimension. If the true signals can only be revealed after adjusting for the confounder, for example, when the true and confounding signals co-locate with opposite directions, unadjusted statistics will not be informative. In this case, the best cutoff on $\mid {Z}_i^U\mid$ should be 0 and 2dFDR is then reduced to the traditional 1dFDR-A. However, in finite samples, it may not always be possible to reduce 2dFDR to 1dFDR-A exactly and 2dFDR could be less powerful than 1dFDR-A in such situations (Additional file 2: Figure S10A, 10% true and confounding signals co-locate with opposite directions**)**.

When the correlation between the variable of the interest and the potential confounder is small, adjusting the confounder will lead to power improvement if the confounder has large effects on the outcome and will not hurt the power much if the confounder has no effects on the outcome. Therefore, in such situation, performing adjusted analyses is a good choice. In fact, when cor(x, z) is 0, the test statistic ${Z}_i^U$ and ${Z}_i^A$ are almost perfectly correlated (Additional file 2: Figure 10B), and performing two-dimensional FDR control based on ${Z}_i^U$ and ${Z}_i^A$ is almost equivalent to performing one-dimensional FDR control based on ${Z}_i^A$. Again, in finite samples, there could be some power loss for 2dFDR (Additional file 2: Figure 10B). Fortunately, cor(x, z) can be known before the analysis. We thus do not recommend running 2dFDR when cor(x, z) is small.

The proposed method belongs to the general topic of using auxiliary data to increase power in multiple testing, which has been an active research area recently [24, 28–32]. To be effective, the auxiliary data has to be informative of the probability of the null hypothesis or the statistical power. In our case, the unadjusted statistic can be considered as a particular type of auxiliary data, which informs the prior null probability (the smaller the statistic, the more likely the null hypothesis). However, as the auxiliary data (unadjusted statistic) and the primary data (adjusted statistic) are correlated even under the null hypothesis, existing structure-adaptive multiple testing methods are not directly applicable in our problem. 2dFDR explicitly addresses such correlation to enable asymptotic FDR control.

Although developed in the linear model setting, the idea of 2dFDR can be extended to the generalized linear model or generalized linear mixed effects model [33] using the Wald z-statistic. Another interesting extension is to adapt 2dFDR to the setup where the omics features are treated as covariates. For example, in genome-wide association studies (GWAS), the genetic variants are usually modeled as covariates. We expect that the same 2dFDR idea could be applied to GWAS to significantly increase its power, since population stratification, which occurs when samples come from genetically diverse underlying populations, is an important confounder for GWAS [34].

In summary, 2dFDR is a new approach to confounder adjustment under multiple testing. It is powerful, robust, and scalable. As a general methodology, we envision its broad applicability of 2dFDR in omics association studies.

## Methods

### The 2dFDR procedure

Here we give an overview of the method. Details could be found in Additional file 1: Note S2. Consider the following *m* linear models

1 $$ E\left({Y}_{ij}\right)={x}_j{\alpha}_i+{z}_j^T{\beta}_i,1\le j\le n,1\le i\le m $$

where *n* and *m* are the sample size and feature size, respectively, (*Y*_i1_, …, *Y*_*in*_)^*T*^ ∈ *ℝ*^*n* × 1^ is the measurement of the omics feature *i*, ***x*** = (*x*_1_, …, *x*_*n*_)^*T*^ ∈ *ℝ*^*n* × 1^ is the vector of the covariate of interest, (*z*_1_, …, *z*_*n*_)^*T*^ ∈ *ℝ*^*n* × *d*^ is the design matrix for the confounding factors, and *α*_*i*_ ∈ *ℝ*, *β*_*i*_ ∈ *ℝ*^*d* × 1^ are the coefficients associated with the covariate and confounding factors respectively. Under the model, there are four different categories of features to consider: (A) Solely associated with the covariate of interest: *α*_*i*_ ≠ 0, *β*_*i*_ = 0;(B) Solely associated with the confounder: *α*_*i*_ = 0, β_*i*_ ≠ 0; (C) Associated with both the covariate of interest and the confounder: *α*_*i*_ ≠ 0, *β*_*i*_ ≠ 0; (D) Not associated with either the covariate of interest or the confounder: *α*_*i*_ = 0, *β*_*i*_ = 0. Our goal is to develop a multiple testing procedure for simultaneously testing *m* hypotheses *H*_0, *i*_ : *α*_*i*_ = 0 vs *H*_1, *i*_ : *α*_*i*_ ≠ 0 (*i* = 1, …, *m*) in the presence of confounding effects. Let ${Z}_i^A$ be the z-statistic for testing whether *α*_*i*_ = 0 after adjusting for the confounding factors, and ${Z}_i^U$ be the unadjusted version without adjusting for the confounding factors, i.e., setting *β*_*i*_ = 0 in the model. Given the thresholds *t*_1_, *t*_2_ ≥ 0, the 2dFDR procedure can be described as follows:

Dimension 1: Signal enrichment. Use the unadjusted statistic ${Z}_{\mathrm{i}}^U$ to retain more promising features ${S}_1=\Big\{1\le i\le m:\left|{Z}_{\mathrm{i}}^U\right|\ge {t}_1$}.

Dimension 2: Excluding false positives. For *i* ∈ *S*_1_, reject *H*_0, *i*_ with ${Z}_i^A\ge {t}_2.$As a result, the final set of discoveries is given by ${S}_2=\Big\{1\le i\le m:\left|{Z}_i^U\right|\ge {t}_1,\left|{Z}_i^U\right|\ge {t}_2$}.

Given *t*_1_, *t*_2_ ≥ 0 , the FDP is defined as

2 $$ \mathrm{FDP}\left({t}_1,{t}_2\right)=\frac{\sum_{i=1}^m\mathbf{1}\left\{|{Z}_i^U|\ge {t}_1,|{Z}_i^A|\ge {t}_2,{\alpha}_i=0\right\}}{1\vee {\sum}_{i=1}^m\mathbf{1}\left\{|{Z}_i^U|\ge {t}_1,|{Z}_i^A|\ge {t}_2\right\}} $$

The key here is to select the thresholds *t*_1_, *t*_2_ to maximize the number of discoveries while controlling the FDR (expectation of FDP) at the desired level.

As the number of false rejections is unknown (the numerator of FDP(t_1_, t_2_)), we replace it by the expected number of false rejections. We can show that the expected number is given by ${\sum}_{i:{\alpha}_i=0}L\left({\mu}_i,{t}_1,{t}_2\right)=P\left(\left|{V}_1+{\mu}_i\right|\ge {t}_1,\left|{V}_2\right|\ge {t}_2\right)$ with *μ*_*i*_ being a nuisance parameter that depends on the confounding effect (the magnitude of *β*_*i*_ and the correlation between x and *z*) and (*V*_1_, *V*_2_) being bivariate mean-zero normal random variables, whose covariance can be calculated from the data (Additional file 1: Note S2.2). Note that the correlation between *V*_1_ and *V*_2_ is not equal to zero in general, which captures the dependence between the two dimensions.

An intrinsic difficulty here is that the expected number of false rejections depends on the effects of the confounding factors (i.e., *μ*_*i*_) on each feature. As the number of features could be huge, it thus requires estimating a large number of nuisance parameters. To tackle this challenge, we adopt a Bayesian viewpoint by assuming that the nuisance parameters *μ*_*i*_ are generated from a common prior distribution *G*. The Bayesian viewpoint allows us to express the expected number of false rejections as a functional of the prior distribution. Therefore, we can translate the task into estimating the prior distribution instead of the direct estimation of a large number of nuisance parameters. The prior distribution can be estimated via the general maximum likelihood empirical Bayes estimation [19, 35], which can be cast into a convex optimization problem (Additional file 1: Note S2.3). Denote $\hat{G}$ the estimate of *G*.Then we can derive an approximate upper bound for FDP(t_1_, t_2_)), as follows:

3 $$ \mathrm{FDP}\left({t}_1,{t}_2\right)\lesssim \frac{m^{-1}{\sum}_{i=1}^mL\left({\mu}_i,{t}_1,{t}_2\right)}{m^{-1}{\sum}_{i=1}^m\mathbf{1}\left\{\left|{Z}_i^U\right|\ge {t}_1,\left|{Z}_i^A\right|\ge {t}_2\right\}}\approx \frac{\int L\left(\mu, {t}_1,{t}_2\right)d\hat{\mathrm{G}}\left(\mu \right)}{m^{-1}{\sum}_{i=1}^m\mathbf{1}\left\{\left|{Z}_i^U\right|\ge {t}_1,\left|{Z}_i^A\right|\ge {t}_2\right\}}:= \hat{\mathrm{FDP}}\left({t}_1,{t}_2\right). $$

For a desired FDR level α ∈ (0, 1), we choose the optimal threshold such that

4 $$ \left({t}_1^{\ast },{t}_2^{\ast}\right)={\mathrm{argmax}}_{\left({t}_1,{t}_2\right)\in {\mathrm{\mathcal{F}}}_{\alpha }}{\sum}_{i=1}^m1\left\{\left|{Z}_i^U\right|\ge {t}_1,\left|{Z}_i^A\right|\ge {t}_2\right\}, $$

where ${\mathcal{F}}_{\alpha }=\left\{\left({t}_1,{t}_2\right)\in {\mathbb{R}}^{+}\times {\mathbb{R}}^{+}:\hat{\mathrm{FDP}}\left({t}_1,{t}_2\right)\le \alpha \right\}.$ Finally, we select features with $\left|{Z}_i^U\right|\ge {t}_1^{\ast },\left|{Z}_i^A\right|\ge {t}_2^{\ast }$. This procedure can be viewed as two-dimensional generalization of the Benjamini-Hochberg (BH) procedure [12]. It is well known that when the number of signals is a substantial proportion of the total number of hypotheses, the BH procedure will be overly conservative. To adapt to the signal density, we develop a modification of John Storey’s approach [13] in our setting (Additional file 1: Note S2.4).

### Data simulation

We conduct comprehensive simulations to evaluate the finite-sample performance of the proposed method and compare it to competing methods. For genome-scale multiple testing, the number of hypotheses could range from thousands to millions. For demonstration purposes, we start with *m* = 10,000 features, *n* = 100 samples, one covariate of interest **x**,and one confounder **z**. To comprehensively evaluate the proposed method’s performance under different scenarios, we study the following important parameters:

- The correlation between the covariate of interest (**x**) and the confounder (**z**) (denoted as “cor(x, z)”);
- The density and strength of the true signals;
- The density and strength of the confounding signals;
- The degree of colocation of the true signals and confounding signals.

To induce the correlation between **x** and **z**, we let **x**_0_~*N*(0, 1), **x** = *c***x**_0_ + *N*(0, 1), **z** = *c***x**_0_ + *N*(0, 1). We set *c* = 0.5, 1.25 and 2, which achieves a correlation (ρ) about 0.2, 0.6, and 0.8 respectively, representing weak (“+”), moderate (“++”) and strong confounding (“+++”) level. For the multiple-confounder scenario, we simulate each **z** in the same way.

Next, we generate

5 $$ {\mathbf{y}}_i={\alpha}_i\mathbf{x}+{\beta}_i\mathbf{z}+{\boldsymbol{\upepsilon}}_i,\kern0.5em \left(i=1,\dots, m\right) $$

where ${\alpha}_i,{\beta}_i\sim \frac{\uppi}{2}\  Unif\Big(-$*l*$-\updelta, -l\Big)+\frac{\uppi}{2} Unif\left(l,l+\delta \right)+\left(1-\uppi \right)\ {I}_0$, *I*_0_ is a mass at 0 and ***ϵ***_*i*_~*N*(0, 1). For both the covariate of interest and the confounder, we simulate three levels of signal density (π = 5 % , 10%, and 20%—representing low, medium, and high density) and three levels of signal strength (δ = 0.2 and *l* = 0.2, 0.3 and 0.4, representing weak, moderate, and strong effect). In the basic setup, *α*_*i*_, *β*_*i*_ are independently drawn from the distribution mentioned above. To study the effect of the colocation of the true and confounding signals (nonzero *α*_*i*_ and *β*_*i*_), we further simulate two scenarios:

- Non-colocation: ∀j, *α*_*i*_*β*_*i*_ = 0
- 50% colocation: ∣S_αβ_ ∣  = 0.5 min{| S_α_| , | S_β_| }, where S_αβ_ = {*i* |*α*_*i*_*β*_*i*_ ≠ 0}, S_α_ = {i | *α*_*i*_ ≠ 0}, S_β_ = {*i*|*β*_*i*_ ≠ 0}.

In addition, we investigate the robustness of the proposed method to correlated errors. Specifically, we study:

- First-order autoregressive (AR(1)) correlation structure, where ρ(**ϵ**_*i*_, **ϵ**_*k*_) = 0.6^|*i* − *k*|^.
- Block correlation structure, where we simulate 100 blocks with within-block correlation 0.6.

We compare 2dFDR to the following methods:

- 1dFDR-U: linear regression with the covariate of interest without adjusting for the confounder.
- 1dFDR-A: linear regression with the covariate of interest adjusting for the confounder, which is the traditional procedure.
- 2dFDR-H: a heuristic hybrid procedure, which first runs “1dFDR-A,” and if the confounder is not significant (nominal *p* < 0.05), “1dFDR-U” is used.

All the methods use the q-value approach for FDR control [13], following the computation of feature-wise *p* values. We evaluate the performance based on FDR control (false discovery proportion) and power (true positive rate) at a target FDR level of 5%. Results are averaged over 100 simulation runs (for small feature sizes, 1000 simulation runs are performed to reduce variability). Both the means and 95% CIs are reported in the bar plots.

### Real datasets

#### Transcriptomics dataset

The dataset consists of 342 RNA-Seq samples from patients with hepatocellular carcinoma from The Cancer Genome Atlas (TCGA) [20]. We use this dataset to identify gene expressions associated with chronic infection of the hepatitis B virus (HBV). HBV status was examined by counting the percentage of reads mapped to HBV genome [36], resulting in 103 and 239 HBV-positive and HBV-negative cases. Ethnicity and gender are confounders in this analysis since they are correlated with the HBV status (OR 0.051 and 2.67, respectively, *p* < 0.001) and are known to affect the gene expressions. The raw FASTQ files were processed through Mayo’s internal MAP-RSeq pipeline (Version 3.0) [37]. The gene counts were generated by FeatureCounts using the gene definitions files from Ensembl v78 [38]. Quality control was carried out using RSeqQC [39], and a total of 19,329 genes were included. Transcript per million (TPM) was calculated, quantile normalized, and log-transformed before analysis.

#### Metabolomics dataset

The dataset came from a study of the impact of the gut microbiome on host serum metabolome and insulin sensitivity in non-diabetic Danish adults [22]. It consists of measurements of 1201 metabolites (325 serum polar metabolites and 876 serum molecular lipids) on 289 serum samples using mass spectrometry. The cleaned dataset was downloaded from https://bitbucket.org/hellekp/clinical-micro-meta-integration [23]. We use this data set to identify insulin resistance (IR)-associated metabolites. IR was estimated by the homeostatic model assessment [22]. Body mass index (BMI) is a confounder for this dataset since it is highly correlated with IR (Spearman’s *ρ* = 0.67) and is known to affect the serum metabolome. Two samples without IR measurement were excluded. For metabolites with zero measurements, zeros were replaced by half of the minimal nonzero value. Log transformation was performed to make the data more symmetrically distributed before analysis.

#### Epigenomics datasets

The datasets came from 51 epigenome-wide association studies (EWAS) of various phenotypes using Infinium Human Methylation 450 K BeadChip. They were collected and processed as previously described [24]. A total of 54 datasets with binary or continuous phenotypes and sample sizes larger than 100 were included in the evaluation (Additional file 2: Table S1). Since these EWAS studies all used tissue samples, which consist of a mixture of different cell types, each with a distinct methylation profile, the shift in the cell mixture proportions with regard to the phenotype of interest could strongly confound the association analysis [5]. For the peripheral blood sample, it consists of different leukocyte subtypes, whose composition usually changes with the onset of disease as a host immune defense mechanism. Since the cell mixture proportions were not directly available for these datasets, we used the surrogate variable analysis [25] to infer the latent factors (also called surrogate variables) that capture the cell mixtures. Specifically, the “isva” R package [40] was used to estimate the number of surrogate variables based on random matrix theory, and the “SmartSVA” R package [18] was used to compute the surrogate variables. The inferred surrogate variables were highly correlated with the phenotypes (a median R^2^ 0.49, Additional file 2: Table S1) and were adjusted in the regression analysis. All the analysis was performed on the methylation M-values [41], and 5% FDR was used to identify differentially methylated CpG positions (DMPs).

## Supplementary information

**Additional file 1: Note S1.** Simulation Setup for Fig. 1a-c. **Note S2.** Full Method Description. **Note S3.** Asymptotic FDR Control. **Note S4.** Power analysis.

**Additional file 2: Figure S1.** Performance comparison when 50% of the features are affected by the confounder. **Figure S2.** Performance on simulated datasets across varying density (top to bottom) and strength (left to right) of the confounding signals when there are five confounders. **Figure S3.** Performance across varying density (top to bottom) and strength (left to right) of the confounding signals when the confounding and true signals do not overlap (“NoCoLoc”) and when the true and confounding signals have extensive overlap (“CoLoc”). **Figure S4.** Performance across varying density (top to bottom) and strength (left to right) of the confounding signals when the errors have a block correlation structure. **Figure S5.** Performance across varying density (top to bottom) and strength (left to right) of the confounding signals when the errors have the first-order auto-regressive (AR(1)) correlation structure. **Figure S6.** Performance comparison across varying density (top to bottom) and strength (left to right) of the confounding signals under smaller sample sizes. **Figure S7.** Performance comparison across varying density (top to bottom) and strength (left to right) of the confounding signals under smaller feature sizes. **Figure S8.** Performance comparison across varying sample size (top to bottom) and feature size (left to right). **Figure S9.** The computation time (in seconds) under different sample sizes and feature sizes based on simulated datasets with one confounder, medium density and strength of the true and confounding signals, and a medium confounding level. **Figure S10.** The decision boundaries of 2dFDR and 1dFDR-A under two unfavorable scenarios for 2dFDR. **Table S1.** EWAS datasets used in the evaluation of the empirical power of 2dFDR.

**Additional file 3.** Review history.

## Data Availability

Codes and data to reproduce the presented results are available at the repository Zenodo (10.5281/zenodo.4977446) [42] and at GitHub (https://github.com/jchen1981/tdfdr) [43] under GNU General Public License v2.0. Detailed documentation and tutorial are available on the GitHub page. Transcriptomics dataset is from [20] and metabolomics dataset from [22]. The epigenomics datasets are listed in Additional file 2: Table S1.

## Abbreviations

EWAS: Epigenome-wide association studies
FDR: False discovery rate
BH: Benjamini-Hochberg step-up procedure
DMPs: Differentially methylated CpG positions
HBV: Hepatitis B virus
BMI: Body mass index
IR: Insulin resistance
SLE: Systemic lupus erythematosus
SVA: Surrogate variable analysis
TCGA: The Cancer Genome Atlas
2dFDR: Two-dimensional false discovery rate control procedure
