# Supplementary material for: 2dFDR: a new approach to confounder adjustment substantially increases detection power in omics association studies
Source: Genome Biol. 2021 Jul 13;22:208. doi: 10.1186/s13059-021-02418-8 (PMC8276451; doi:10.1186/s13059-021-02418-8)
Supplement: Supplementary file 1 — Additional file 1: Note S1. Simulation Setup for Fig. 1a-c. Note S2. Full Method Description. Note S3. Asymptotic FDR Control. Note S4. Power analysis. [file 13059_2021_2418_MOESM1_ESM.pdf]

Additional file 1:
Supplementary Notes for "A new approach to confounder adjustment substantially increases detection power in omics association studies"

Contents

Note S1: Simulation Setup for Fig. 1a-c **3**

Note S2: Full Method Description **4**
2.1 Basic setup 4
2.2 Approximation of the false discovery proportion 5
2.3 Nonparametric empirical Bayes 6
2.4 Two-dimensional Benjamini-Hochberg procedure 7

Note S3: Asymptotic FDR Control **10**
3.1 Statement of the theorem 10
3.2 Full proof of the theorem 12

Note S4: Power analysis **34**

Note S1: Simulation Setup for Fig. 1a-c

To generate Fig. 1a-c, we consider the following linear models:

$$Y_i = X\alpha_i + Z\beta_i + e_i, \quad e_i \overset{\text{i.i.d.}}{\sim} N(0, \sigma_i^2 \mathbf{I}_n), \quad 1 \le i \le m, \tag{1}$$

where $Y_i \in \mathbb{R}^{n\times 1}$ is the response vector, $X = (X_1, \ldots, X_n)^\top \in \mathbb{R}^{n\times 1}$ is the covariate of interest, $Z = (z_1, \ldots, z_n)^\top \in \mathbb{R}^{n\times 1}$ is the confounding factor, and $\alpha_i \in \mathbb{R}$ and $\beta_i \in \mathbb{R}$ are the parameters associated with the covariate and confounding factor, respectively.

The 300 independent simulation runs are conducted with $n = 100$ and $m = 2000$. We simulate $X = (X_1, \cdots, X_{100})^\top, Z = (Z_1, \cdots, Z_{100})^\top$ by

$$X_k \sim N(\rho\varepsilon_k^*, 1), \quad Z_k \sim N(\rho\varepsilon_k^*, 1), \quad k = 1, ..., n. \tag{2}$$

where $\rho \in \{1.0, 1.5, 2.0\}$ controls the degree of confounding, and $\varepsilon_k^* \overset{i.i.d.}{\sim} N(0, 1)$ is the random noise. We generate $Y_i \in \mathbb{R}^{100\times 1}$ under (1) with $\alpha = (\alpha_1, \ldots, \alpha_m)^\top$ and $\beta = (\beta_1, \ldots, \beta_m)^\top$ such that

- Category A : $\alpha_i = 0.5, \quad \beta_i = 0 \quad \text{for} \quad i \in I_A,$
- Category B : $\alpha_i = 0, \quad \beta_i = 0.5 \quad \text{for} \quad i \in I_B,$
- Category C : $\alpha_i = 0.5, \quad \beta_i = 0.5 \quad \text{for} \quad i \in I_C,$
- Category D : $\alpha_i = 0, \quad \beta_i = 0 \quad \text{for} \quad i \in I_D,$

where $I_A, I_B, I_C, I_D \subset \{1, \ldots, m\}$ denote some mutually disjoint index sets (see illustration below).

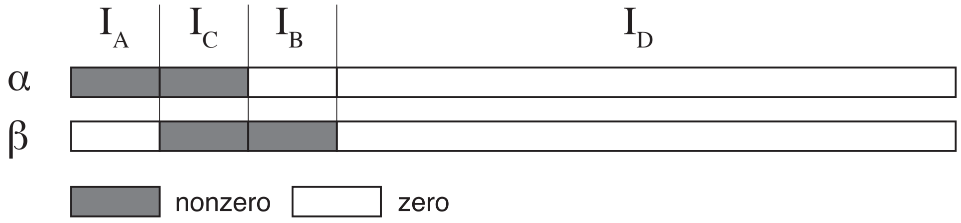

We fix $|I_A| = 67, |I_B| = 66, |I_C| = 66$ and randomly generate these index sets for each simulation run. We compare the proposed two-dimensional FDR control procedure (2dFDR) to the one-dimensional FDR control procedure based on the confounder-adjusted model (1dFDR-A), and the power is evaluated using true positive rate.

Note S2: Full Method Description

Notation. For $x, y \in \mathbb{R}$, let $x \vee y = \max(x, y)$ and $x \wedge y = \min(x, y)$. Let $d_H^2(f, g) = (1/2)\int(\sqrt{f(x)} - \sqrt{g(x)})^2 dx$ be the square Hellinger distance between two densities f and g. For a matrix $\mathbf{C}$, denote by $P_{\mathbf{C}} = \mathbf{C}(\mathbf{C}^\top\mathbf{C})^{-1}\mathbf{C}^\top$ the projection matrix associated with the column space of $\mathbf{C}$ and define $P_{\mathbf{C}}^\perp = \mathbf{I} - P_{\mathbf{C}}$. Let $\|\mathbf{C}\|_2$ and $\|\mathbf{C}\|_{\max}$ be the spectral norm and the elementwise maximum norm of $\mathbf{C}$, respectively. Denote by $\lambda_{\min}(\mathbf{C})$ and $\lambda_{\max}(\mathbf{C})$ the minimum and maximum eigenvalues of $\mathbf{C}$. Let $\phi(\cdot)$ and $\Phi(\cdot)$ be the probability density function and cumulative distribution function of the standard normal distribution. Denote by χ_k^2 the chi-square distribution with k degrees of freedom.

2.1 Basic setup

Consider the following linear models:

$$Y_i = \mathbf{1}_{n\times 1} b + X\alpha_i + \mathbf{Z}\boldsymbol{\beta}_i + e_i, \quad e_i \overset{\text{i.i.d.}}{\sim} N(0, \sigma_i^2 \mathbf{I}_n), \quad 1 \le i \le m, \tag{3}$$

where $Y_i \in \mathbb{R}^{n\times 1}$ is the response vector, $X = (X_1, \ldots, X_n)^\top \in \mathbb{R}^{n\times 1}$ is the covariate of interest, $\mathbf{Z} = (z_1, \ldots, z_n)^\top \in \mathbb{R}^{n\times d}$ is the design matrix associated with the confounding factors, and $\alpha_i \in \mathbb{R}$ and $\boldsymbol{\beta}_i = (\beta_{i1}, \ldots, \beta_{id})^\top \in \mathbb{R}^{d\times 1}$ are the parameters associated with the covariate and confounding factors respectively. By centering the response, covariate and confounding factors, we can assume without loss of generality that $b = 0$ throughout the following discussions.

Under (3), there are four different categories to consider

A. Solely associated with the variable of interest: $\alpha_i \neq 0, \boldsymbol{\beta}_i = 0$;

B. Solely associated with the confounder: $\alpha_i = 0, \boldsymbol{\beta}_i \neq 0$;

C. Associated with both the variable of interest and confounder: $\alpha_i \neq 0, \boldsymbol{\beta}_i \neq 0$;

D. Not associated with either the variable of interest or confounder: $\alpha_i = 0, \boldsymbol{\beta}_i = 0$.

The goal here is to develop a multiple testing procedure for simultaneously testing m hypotheses

$$H_{0,i} : \alpha_i = 0 \quad \text{versus} \quad H_{a,i} : \alpha_i \neq 0, \quad i = 1, 2, \ldots, m,$$

while adjusting for the confounding effects. We let $\hat{\alpha}_i^A$ be the estimator of α_i after adjusting for the confounding effect, and $\hat{\alpha}_i^U$ be the unadjusted version without taking into account the confounding factors. Specifically, we have

$$\hat{\alpha}_i^A = (X^\top P_{\mathbf{Z}}^\perp X)^{-1} X^\top P_{\mathbf{Z}}^\perp Y_i = \alpha_i + (X^\top P_{\mathbf{Z}}^\perp X)^{-1} X^\top P_{\mathbf{Z}}^\perp e_i,$$
$$\hat{\alpha}_i^U = (X^\top X)^{-1} X^\top Y_i = \alpha_i + (X^\top X)^{-1} X^\top \mathbf{Z}\boldsymbol{\beta}_i + (X^\top X)^{-1} X^\top e_i.$$

Under (3), the estimator of the noise level σ_i^2 is given by

$$\hat{\sigma}_i^2 = \frac{1}{n-d-1}(Y_i - X\hat{\alpha}_i^A - \mathbf{Z}\hat{\boldsymbol{\beta}}_i)^\top (Y_i - X\hat{\alpha}_i^A - \mathbf{Z}\hat{\boldsymbol{\beta}}_i) = \frac{1}{n-d-1} Y_i^\top P_{\mathbf{W}}^\perp Y_i,$$

where $\hat{\boldsymbol{\beta}}_i = (\mathbf{Z}^\top P_X^\perp \mathbf{Z})^{-1} \mathbf{Z}^\top P_X^\perp Y_i$ and $\mathbf{W} = (X, \mathbf{Z})$. Let $\Omega = X^\top X/n$, $\Gamma = X^\top \mathbf{Z}/n$, $\Omega_{X|\mathbf{Z}} = X^\top P_{\mathbf{Z}}^\perp X/n$ and $\Omega_{\mathbf{Z}|X} = \mathbf{Z}^\top P_X^\perp \mathbf{Z}/n$. The adjusted and unadjusted z-statistics for testing $H_{0,i}$ can

be defined as

$$Z_i^A = \sqrt{n}\Omega_{X|\mathbf{Z}}^{1/2}\hat{\alpha}_i^A/\hat{\sigma}_i = \sqrt{n}\Omega_{X|\mathbf{Z}}^{1/2}\alpha_i/\hat{\sigma}_i + \Omega_{X|\mathbf{Z}}^{-1/2}X^\top P_{\mathbf{Z}}^\perp e_i/(\sqrt{n}\hat{\sigma}_i),$$
$$Z_i^U = \sqrt{n}\Omega^{1/2}\hat{\alpha}_i^U/\hat{\sigma}_i = \sqrt{n}\Omega^{1/2}\alpha_i/\hat{\sigma}_i + \sqrt{n}\Omega^{-1/2}\Gamma\boldsymbol{\beta}_i/\hat{\sigma}_i + \Omega^{-1/2}X^\top e_i/(\sqrt{n}\hat{\sigma}_i),$$

where we have used the variance estimator under model (3) for both statistics. Given the thresholds $t_1, t_2 \geq 0$, the two-dimensional procedure can be described as follows:

Dimension 1. Use the unadjusted statistics to determine a preliminary set of features $\mathcal{D}_1 = \{1 \leq i \leq m : |Z_i^U| \geq t_1\}$.

Dimension 2. Reject $H_{0,i}$ for $|Z_i^A| \geq t_2$ and $i \in \mathcal{D}_1$. As a result, the final set of discoveries is given by $\mathcal{D}_2 = \{1 \leq i \leq m : |Z_i^U| \geq t_1, |Z_i^A| \geq t_2\}$.

The basic idea of this procedure is to use the unadjusted statistics to screen out a large number of noises in Category D and further use the adjusted statistics to identify signals from Categories A and C. Although the unadjusted statistics are unable to distinguish the noise in Category B from the signals, they can preserve or even increase the signal strength. To see this, we note that

$$|\Omega^{1/2}\alpha_i| \geq |\Omega_{X|Z}^{1/2}\alpha_i|.$$

When $\boldsymbol{\beta}_i = 0$, the unadjusted statistics can better preserve the signal strength comparing to the adjusted one.

2.2 Approximation of the false discovery proportion

Recall that t_i is the threshold in Dimension i for $i = 1, 2$. We propose a method to simultaneously select the two thresholds. Note that the ith hypothesis will be rejected if and only if

$$|Z_i^U| \geq t_1 \quad \text{and} \quad |Z_i^A| \geq t_2.$$

In Dimension 2, all rejections from Categories B and D ($\alpha_i = 0$) will be considered as false rejections/discoveries. Therefore, the false discovery proportion (FDP) is defined as

$$\begin{aligned}
&\text{FDP}(t_1, t_2) \\
=&\frac{\sum_{i:\alpha_i=0} \mathbf{1}\{|Z_i^U| \geq t_1, |Z_i^A| \geq t_2\}}{\sum_{i=1}^m \mathbf{1}\{|Z_i^U| \geq t_1, |Z_i^A| \geq t_2\}} \\
=&\frac{\sum_{i:\alpha_i=0} \mathbf{1}\{|\sqrt{n}\Omega^{-1/2}\Gamma\boldsymbol{\beta}_i/\hat{\sigma}_i + \Omega^{-1/2}X^\top e_i/(\sqrt{n}\hat{\sigma}_i)| \geq t_1, |\Omega_{X|\mathbf{Z}}^{-1/2}X^\top P_{\mathbf{Z}}^\perp e_i/(\sqrt{n}\hat{\sigma}_i)| \geq t_2\}}{\sum_{i=1}^m \mathbf{1}\{|Z_i^U| \geq t_1, |Z_i^A| \geq t_2\}}. \qquad (4)
\end{aligned}$$

Let (V_1, V_2) be the bivariate normal random variables such that

$$\begin{pmatrix} V_1 \\ V_2 \end{pmatrix} \sim N\left(\begin{pmatrix} 0 \\ 0 \end{pmatrix}, \begin{pmatrix} 1 & \Omega^{-1/2}\Omega_{X|\mathbf{Z}}^{1/2} \\ \Omega_{X|\mathbf{Z}}^{1/2}\Omega^{-1/2} & 1 \end{pmatrix}\right).$$

Replacing the numerator by the corresponding expectation (conditional on $X, \mathbf{Z}$ and $\boldsymbol{\beta}_i$'s) and $\hat{\sigma}_i$ by σ_i in (4), we obtain

$$\mathrm{FDP}(t_1, t_2) \approx \frac{\sum_{i:\alpha_i=0} L(\mu_i, t_1, t_2)}{\sum_{i=1}^m \mathbf{1}\{|Z_i^U| \geq t_1, |Z_i^A| \geq t_2\}} \leq \frac{\sum_{i=1}^m L(\mu_i, t_1, t_2)}{\sum_{i=1}^m \mathbf{1}\{|Z_i^U| \geq t_1, |Z_i^A| \geq t_2\}}, \tag{5}$$

where $\mu_i = \mu_{i,n} := \sqrt{n}\Omega^{-1/2}\Gamma\boldsymbol{\beta}_i/\sigma_i$ and $L(\mu, t_1, t_2) = P\left(|\mu + V_1| \geq t_1, |V_2| \geq t_2|\mu\right)$.

The major challenge here is the estimation of the expected number of false rejections given by $\sum_{i=1}^m L(\mu_i, t_1, t_2)$, which involves a large number of nuisance parameters μ_i's. A natural strategy is to estimate each μ_i separately by $\hat{\mu}_i$, and replace $L(\mu_i, t_1, t_2)$ by the plug-in estimate $L(\hat{\mu}_i, t_1, t_2)$. It seems natural to use the least squares estimator given by $\hat{\mu}_i = \sqrt{n}\Omega^{-1/2}\Gamma\hat{\boldsymbol{\beta}}_i/\hat{\sigma}_i$. However, this method does not lead to a consistent estimation of the number of false rejections when there is non-negligible proportion of weak confounding factors.[1] To see this, we note that $\hat{\mu}_i$ approximately follows a normal distribution with mean μ_i and variance $A^2 = \Omega^{-1/2}\Gamma\Omega_{\mathbf{Z}|X}^{-1}\Gamma^T\Omega^{-1/2}$. Some algebra shows that

$$L(\hat{\mu}_i, t_1, t_2) \approx P(|\mu_i + \tilde{V}_1| \geq t_1, |\tilde{V}_2| \geq t_2|\mu_i) \neq L(\mu_i, t_1, t_2),$$

where

$$\begin{pmatrix}\tilde{V}_1\\ \tilde{V}_2\end{pmatrix} \sim N\left(\begin{pmatrix}0\\0\end{pmatrix}, \begin{pmatrix}1 + A^2 & \Omega^{-1/2}\Omega_{X|\mathbf{Z}}^{1/2}\\ \Omega_{X|\mathbf{Z}}^{1/2}\Omega^{-1/2} & 1\end{pmatrix}\right).$$

Compared to the joint distribution of (V_1, V_2), we see that the least squares estimator introduces extra variation to the first component of the bivariate normal distribution. We have also considered soft and hard thresholding estimators for μ_i. The consistency of these regularized estimators requires a minimum signal assumption which again rules out the case of weak confounding factors. To overcome the difficulty, we shall adopt a Bayesian viewpoint by assuming that μ_i's are generated from a common prior distribution. The Bayesian viewpoint allows us to borrow cross-sectional information (from different linear models) to estimate the number of false rejections without estimating individual μ_i explicitly.

2.3 Nonparametric empirical Bayes

In this subsection, we propose a nonparametric empirical Bayes approach to estimate the number of false rejections. Define

$$\hat{\mathbf{a}} = \frac{\Omega^{-1/2}\Gamma}{\sqrt{\Omega^{-1/2}\Gamma\Omega_{\mathbf{Z}|X}^{-1}\Gamma^\top\Omega^{-1/2}}}, \quad \hat{\boldsymbol{\xi}}_i = \sqrt{n}\hat{\boldsymbol{\beta}}_i/\sigma_i, \quad \boldsymbol{\xi}_i = \sqrt{n}\boldsymbol{\beta}_i/\sigma_i.$$

Under (3) and conditional on $\mathbf{W} = (X, \mathbf{Z})$, we have the Gaussian location model,

$$\hat{\eta}_i = \eta_i + \epsilon_i \tag{6}$$

where $\hat{\eta}_i = \hat{\mathbf{a}}^\top\hat{\boldsymbol{\xi}}_i$ is an estimator for $\eta_i = \hat{\mathbf{a}}^\top\boldsymbol{\xi}_i$ and $\epsilon_i \overset{\text{i.i.d.}}{\sim} N(0, 1)$.

Suppose $\boldsymbol{\xi}_i$'s are independently generated from some distribution, see Assumption .5. Under

[1] A confounding factor is said to be weak if its coefficient $\boldsymbol{\beta}_i$ decays to zero at the rate $n^{-1/2}$ or faster. In this case, $\limsup_{n\to+\infty} |\mu_{i,n}| = \delta_i \in [0, +\infty)$.

suitable assumptions detailed below, we can show that $\hat{\mathbf{a}} \rightarrow^{a.s.} \mathbf{a}$ for a vector $\mathbf{a}$ defined in equation (11). Denote by G_0 the (prior) distribution for $\mathbf{a}^\top \boldsymbol{\xi}_i$. The goal here is to estimate G_0 based on $\{\hat{\eta}_i\}$. It will become clear later that how the estimate of G_0 is useful in estimating the expected number of false rejections. Following Kiefer and Wolfowitz (1956) and Jiang and Zhang (2009), we consider the general maximum likelihood estimator (GMLE) $\widehat{G}_{m,n}$ for G_n defined as

$$\widehat{G}_{m,n} = \underset{G \in \mathcal{G}}{\text{argmax}} \sum_{i=1}^{m} \log f_G(\hat{\eta}_i) \tag{7}$$

where $\mathcal{G}$ denotes the set of all probability distributions on $\mathbb{R}$ and $f_G(x) = \int \phi(x-u)dG(u)$ is the convolution between G and ϕ. As σ_i's are generally unknown in practice, $\widehat{G}_{m,n}$ is not obtainable. To obtain a feasible estimator, we consider the GMLE $\widetilde{G}_{m,n}$ defined as

$$\widetilde{G}_{m,n} = \underset{G \in \mathcal{G}}{\text{argmax}} \sum_{i=1}^{m} \log f_G(\tilde{\eta}_i) \tag{8}$$

where $\tilde{\eta}_i = \hat{\mathbf{a}}^\top \tilde{\boldsymbol{\xi}}_i$ for $\tilde{\boldsymbol{\xi}}_i = \sqrt{n}\hat{\boldsymbol{\beta}}_i / \hat{\sigma}_i$. By the Carathéodory's theorem, there exist discrete solutions to (7) and (8) with no more than $m+1$ support points . Thus we can write the solutions as

$$\widehat{G}_{m,n}(u) = \sum_{j=1}^{m} \hat{\pi}_j \mathbf{1}\{\hat{s}_j \le u\}, \quad \widetilde{G}_{m,n}(u) = \sum_{j=1}^{m} \tilde{\pi}_j \mathbf{1}\{\tilde{s}_j \le u\}$$

where $\sum_{j=1}^{m} \hat{\pi}_j = \sum_{j=1}^{m} \tilde{\pi}_j = 1$ for $\hat{\pi}_j, \tilde{\pi}_j \ge 0$, and $\{\hat{v}_1, \cdots, \hat{v}_m\}$ and $\{\tilde{v}_1, \cdots, \tilde{v}_m\}$ are two sets of support points for $\widehat{G}_{m,n}$ and $\widetilde{G}_{m,n}$, respectively. From the definitions of $\widehat{G}_{m,n}$ and $f_{\widehat{G}_{m,n}}$, the support of $\widehat{G}_{m,n}(u)$ is always within the range of $\hat{\eta}_i$ due to the monotonicity of $\phi(x-u)$ in $|x-u|$. Similarly, the support of $\widetilde{G}_{m,n}(u)$ is always within the range of $\tilde{\eta}_i$. These observations would be useful for our theoretical analysis, see Section 3.2. It is also worth noting that the optimization in (8) can be cast as convex optimization problem that can be efficiently solved by modern interior point methods. The readers are referred to Koenker and Mizera (2014) for more detailed discussions.

2.4 Two-dimensional Benjamini-Hochberg procedure

Given the feasible estimator $\widetilde{G}_{m,n}$ of the prior distribution and in view of (5), we consider an approximate upper bound for $\text{FDP}(t_1, t_2)$ defined as

$$\widetilde{\text{FDP}}(t_1, t_2) := \frac{\sum_{i=1}^{m} \int L(Ax, t_1, t_2) d\widetilde{G}_{m,n}(x)}{\sum_{i=1}^{m} \mathbf{1}\{|Z_i^U| \ge t_1, |Z_i^A| \ge t_2\}} = \frac{m \int L(Ax, t_1, t_2) d\widetilde{G}_{m,n}(x)}{\sum_{i=1}^{m} \mathbf{1}\{|Z_i^U| \ge t_1, |Z_i^A| \ge t_2\}}.$$

For a desired FDR level $q \in (0,1)$, we choose the optimal threshold such that

$$(T_1^*, T_2^*) = \underset{(t_1,t_2) \in \mathcal{F}_q}{\text{argmax}} \sum_{i=1}^{m} \mathbf{1}\{|Z_i^U| \ge t_1, |Z_i^A| \ge t_2\} \tag{9}$$

where $\mathcal{F}_q = \{(t_1, t_2) \in \mathbb{R}^+ \times \mathbb{R}^+ : \widetilde{\text{FDP}}(t_1, t_2) \le q\}$ with $\mathbb{R}^+ = (0, +\infty)$. This procedure can be viewed as a variant of the Benjamini-Hochberg (BH) procedure adapted to the two-dimensional approach introduced in Section 2.1.

Remark 1. The rejection region we consider is of the form

$$\{(z^U, z^A) : |z^U| \geq t_1, |z^A| \geq t_2\}.$$

In particular, if $t_1 = 0$, it reduces to the usual rejection region $\{z^A : |z^A| \geq t_2\}$ from the one-dimensional approach based on the adjusted statistics. Therefore, our approach produces at least as many rejections as the one-dimensional approach as we are searching over a larger class of rejection regions to maximize the number of discoveries.

It is well known that when the number of signals is a substantial proportion of the total number of hypotheses, the BH procedure will be overly conservative. To adapt to the proportion of signals, we develop a modification of John Storey's approach [Storey (2002)] in our setting. To illustrate the idea, we assume that Z_i^A approximately follows the mixture model:

$$\pi_{0i} N(0,1) + (1 - \pi_{0i}) N(\mu_i^A, 1)$$

where $\mu_i^A = \sqrt{n} \Omega_{X|\mathbf{Z}}^{1/2} \alpha_i / \sigma_i$ and π_{0i} denotes the prior probability that $\alpha_i = 0$.[2] Notice that

$$P(|Z_i^A| \leq \lambda) = \pi_{0i}(1 - 2\Phi(-\lambda)) + (1 - \pi_{0i}) P(|N(\mu_i^A, 1)| \leq \lambda) \approx \pi_{0i}(1 - 2\Phi(-\lambda)),$$

provided that $P(|N(\mu_i^A, 1)| \leq \lambda) \approx 0$. Thus $\mathbf{1}\left\{|Z_i^A| \leq \lambda\right\} / \{1 - 2\Phi(-\lambda)\}$ can be viewed as a conservative estimator for the mixing probability π_{0i}. We note

$$\begin{aligned} \frac{1}{m} \sum_{i:\alpha_i = 0} L(\mu_i, t_1, t_2) =& \frac{1}{m} \sum_{i=1}^m \mathbf{1}\{\alpha_i = 0\} L(\mu_i, t_1, t_2) \\ \approx& \int L(Ax, t_1, t_2) d\widetilde{G}_{m,n}(x) \frac{1}{m} \sum_{i=1}^m \pi_{0i} \\ \leq& \int L(Ax, t_1, t_2) d\widetilde{G}_{m,n}(x) \frac{1}{m} \sum_{i=1}^m \frac{P(|Z_i^A| \leq \lambda)}{1 - 2\Phi(-\lambda)} \\ \approx& \int L(Ax, t_1, t_2) d\widetilde{G}_{m,n}(x) \frac{1}{m} \sum_{i=1}^m \frac{\mathbf{1}\left\{|Z_i^A| \leq \lambda\right\}}{1 - 2\Phi(-\lambda)}, \end{aligned}$$

where to get the first approximation, we implicitly assume that the prior distribution of μ_i remains the same regardless of whether α_i is equal to zero or not. In view of the above derivation, we consider the FDR estimate given by

$$\widetilde{\mathrm{FDP}}_\lambda(t_1, t_2) = \frac{\int L(Ax, t_1, t_2) d\widetilde{G}_{m,n}(x) \sum_{i=1}^m \mathbf{1}\left\{|Z_i^A| \leq \lambda\right\}}{(1 - 2\Phi(-\lambda)) \sum_{i=1}^m \mathbf{1}\left\{|Z_i^U| \geq t_1, |Z_i^A| \geq t_2\right\}},$$

where λ is a prespecified number as in John Storey's approach. With this modification, for a desired FDR level $q \in (0,1)$, we choose the optimal threshold such that

$$(\widetilde{T}_1^*, \widetilde{T}_2^*) = \underset{(t_1, t_2) \in \mathcal{F}_{q,\lambda}}{\operatorname{argmax}} \sum_{i=1}^m \mathbf{1}\left\{|Z_i^U| \geq t_1, |Z_i^A| \geq t_2\right\}, \tag{10}$$

[2] We emphasize that the validity of our procedure does not rely on the mixture model assumption which is merely used to motivate John Storey's procedure.

where

$$\mathcal{F}_{q,\lambda} := \left\{ (t_1, t_2) \in \mathbb{R}^+ \times \mathbb{R}^+ : \widetilde{\mathrm{FDP}}_\lambda(t_1, t_2) \leq q \right\}.$$

Note S3: Asymptotic FDR Control

3.1 Statement of the theorem

The two-dimensional procedure is shown to provide asymptotic FDR control under suitable assumptions. Denote by m_0 and m_1 the number of null and alternative hypotheses among the m hypotheses respectively. Let $\breve{Z}_i^U$ and $\breve{Z}_i^A$ be the z-statistics by replacing $\hat{\sigma}_i$ with σ_i in Z_i^U and Z_i^A, respectively. Define $L_0(\mu, t_1, t_2) = \mathbb{P}(|\mu + \breve{V}_1| \geq t_1, |\breve{V}_2| \geq t_2 | \mu)$ where

$$\begin{pmatrix} \breve{V}_1 \\ \breve{V}_2 \end{pmatrix} \sim N\left(\begin{pmatrix} 0 \\ 0 \end{pmatrix}, \begin{pmatrix} 1 & \mathbb{E}[\Omega]^{-1/2} C_{X|\mathbf{Z}}^{1/2} \\ C_{X|\mathbf{Z}}^{1/2} \mathbb{E}[\Omega]^{-1/2} & 1 \end{pmatrix}\right)$$

with $C_{X|\mathbf{Z}} = \mathbb{E}[\Omega] - \mathbb{E}[\Gamma]\mathbb{E}[\Psi]^{-1}\mathbb{E}[\Gamma]^\top$ for $\Psi = \mathbf{Z}^\top \mathbf{Z}/n$. We also let $A_0^2 = \mathbb{E}[\Omega]^{-1/2}\mathbb{E}[\Gamma] C_{\mathbf{Z}|X}^{-1} \mathbb{E}[\Gamma]^\top \mathbb{E}[\Omega]^{-1/2}$ and

$$\mathbf{a}^\top = \frac{\mathbb{E}[\Omega]^{-1/2}\mathbb{E}[\Gamma]}{\sqrt{\mathbb{E}[\Omega]^{-1/2}\mathbb{E}[\Gamma] C_{\mathbf{Z}|X}^{-1} \mathbb{E}[\Gamma]^\top \mathbb{E}[\Omega]^{-1/2}}} \tag{11}$$

where $C_{\mathbf{Z}|X} = \mathbb{E}[\Psi] - (\mathbb{E}[\Omega])^{-1}\mathbb{E}[\Gamma]^\top \mathbb{E}[\Gamma]$. We first introduce the following definitions and assumptions.

Definition .1. A random variable $X \in \mathbb{R}$ is said to be sub-gaussian with the variance proxy σ^2 if $\mathbb{E}[X] = 0$ and its moment generating function satisfies

$$\mathbb{E}[\exp(tX)] \leq \exp\left(\frac{\sigma^2 t^2}{2}\right) \quad \text{for any } t \in \mathbb{R}.$$

Definition .2. A random variable $X \in \mathbb{R}$ is said to be sub-exponential with the parameter θ if $\mathbb{E}[X] = 0$ and its moment generating function satisfies

$$\mathbb{E}[\exp(tX)] \leq \exp\left(\frac{\theta^2 t^2}{2}\right) \quad \text{for any } |t| \leq \frac{1}{\theta}.$$

Assumption .1. *Suppose $m_0/m \to \pi_0 \in (0,1)$.*

Assumption .2. *Assume that*

$$\frac{\sum_{i:\alpha_i=0} \mathbf{1}\left\{|\breve{Z}_i^U| \geq t_1, |\breve{Z}_i^A| \geq t_2\right\}}{m_0} \overset{a.s.}{\to} K_0(t_1, t_2),$$
$$\frac{\sum_{i:\alpha_i\neq 0} \mathbf{1}\left\{|\breve{Z}_i^U| \geq t_1, |\breve{Z}_i^A| \geq t_2\right\}}{m_1} \overset{a.s.}{\to} K_1(t_1, t_2),$$

for every $(t_1, t_2) \in \mathbb{R}^+ \times \mathbb{R}^+$, where

$$K_0(t_1, t_2) = \mathbb{E}_{\mathbf{a}^\top \boldsymbol{\xi}}[L_0(A_0 \mathbf{a}^\top \boldsymbol{\xi}, t_1, t_2)] \quad \text{for} \quad \mathbf{a}^\top \boldsymbol{\xi} \sim G_0 \tag{12}$$

and $K_0(t_1, t_2), K_1(t_1, t_2)$ are both non-negative continuous functions of the arguments (t_1, t_2).

Assumption .3. *Assume that*

$$\lambda_{\min}(C_{\mathbf{Z}|X}) > 0, \quad \lambda_{\min}(\mathbb{E}[\Psi]) > 0, \quad \mathbb{E}[X_1^2] > 0,$$
$$0 < \min_{1\le i\le m} \sigma_i \le \max_{1\le i\le m} \sigma_i < \infty.$$

Assumption .4. *Assume that the components of* $\mathbf{Z}$ *and* X *are both sub-Gaussian.*

Assumption .5. *Assume that* $\{\boldsymbol{\xi}_i\}_{i=1}^d$ *is a sequence of i.i.d. continuous random vectors with the density* h *whose support set is given by* $\{x \in \mathbb{R}^d, \|x\|_{\max} \le B(\log m)^b\}$ *for some* $B, b \ge 0$.

Assumption .6. *Assume* $m = m(n)$ *such that* $m(n) \to +\infty$ *as* $n \to +\infty$ *and* $\limsup_{n\to+\infty} \frac{m(n)}{n^{p_0}} < \infty$ *for some* $p_0 > 0$.

Assumption .1 requires the asymptotic null proportion to be strictly between zero and one. Assumption .2 allows certain forms of dependence, such as m-dependence, ergodic dependence and certain mixing type dependence. A justification for equation (12) is given in Corollary 1. Assumption .3 implies that $C_{\mathbf{Z}|X}^{-1}$ exists and the noise level is uniformly bounded from below and above. Assumption .4 allows us to use concentration inequalities for sub-Gaussian and sub-exponential random variables in the proofs. Assumption .5 implies that G_0 has a bounded support that expands slowly with m. From Assumption .6, the number of features m is some function of n and m is allowed to be polynomially larger than the sample size n.

Remark 2. The assumption that $\boldsymbol{\xi}_i$ has a density is merely used to simplify the presentation of the proof of Lemma 9. When $\boldsymbol{\xi}_i$ has a discrete distribution, the proof of Lemma 9 can be modified to obtain a similar result. We omit the details here to conserve the space.

Before stating the main result, we introduce the following lemma which establishes the uniform convergence of $\int L(Ax, t_1, t_2)d\widetilde{G}_{m,n}(x)$.

Lemma 1. *Let* $\widetilde{G}_{m,n}$ *be the estimator of* G_0 *as defined in (8). Under Assumptions .3-.6, for any* $t_1', t_2' > 0$, *we have*

$$\sup_{t_1\le t_1', t_2\le t_2'} \left| \int L(Ax, t_1, t_2)d\widetilde{G}_{m,n}(x) - \int L_0(A_0x, t_1, t_2)dG_0(x) \right| \overset{a.s.}{\to} 0.$$

Let

$$V_m(t_1, t_2) = \sum_{i:\alpha_i=0} \mathbf{1}\left\{|Z_i^U| \ge t_1, |Z_i^A| \ge t_2\right\}, \quad S_m(t_1, t_2) = \sum_{i:\alpha_i\ne0} \mathbf{1}\left\{|Z_i^U| \ge t_1, |Z_i^A| \ge t_2\right\},$$
$$F_m(\lambda) = \frac{1}{m}\sum_{i=1}^m \mathbf{1}\left\{|Z_i^A| \le \lambda\right\}, \quad F(\lambda) = \pi_0(1 - 2\Phi(-\lambda)) + (1 - \pi_0)(1 - K_1(0, \lambda)).$$

The following lemma shows the almost sure convergence as in Assumption .2 with $(\breve{Z}_i^U, \breve{Z}_i^A)$ replaced by (Z_i^U, Z_i^A).

Lemma 2. *Under Assumptions .2-.3 and .6, we have*

$$\frac{1}{m_0}V_m(t_1, t_2) \overset{a.s.}{\to} K_0(t_1, t_2), \quad \frac{1}{m_1}S_m(t_1, t_2) \overset{a.s.}{\to} K_1(t_1, t_2), \quad F_m(\lambda) \overset{a.s.}{\to} F(\lambda).$$

Recall that

$$\widetilde{\mathrm{FDP}}_\lambda(t_1,t_2)=\frac{\int L(Ax,t_1,t_2)d\widetilde{G}_{m,n}(x)\sum_{i=1}^m \mathbf{1}\left\{|Z_i^A|\le\lambda\right\}}{(1-2\Phi(-\lambda))\sum_{i=1}^m \mathbf{1}\left\{|Z_i^U|\ge t_1,|Z_i^A|\ge t_2\right\}}$$

and $(\widetilde{T}_1^*,\widetilde{T}_2^*)$ is the optimal threshold as defined in (10). Define

$$\mathrm{FDP}_\lambda^\infty(t_1,t_2):=\frac{\mathbb{E}_{\boldsymbol{a}^\top\boldsymbol{\xi}}[L_0(A_0\boldsymbol{a}^\top\boldsymbol{\xi},t_1,t_2)]\left\{\pi_0(1-2\Phi(-\lambda))+(1-\pi_0)(1-K_1(0,\lambda))\right\}}{(1-2\Phi(-\lambda))K(t_1,t_2)}, \tag{13}$$

where $K(t_1,t_2)=\pi_0K_0(t_1,t_2)+(1-\pi_0)K_1(t_1,t_2)$. By Lemmas 1-2, it follows that

$$\widetilde{\mathrm{FDP}}_\lambda(t_1,t_2)\overset{a.s.}{\to}\mathrm{FDP}_\lambda^\infty(t_1,t_2).$$

We impose the following assumption to reduce the searching region for (t_1,t_2) to a rectangle of the form $[0,t_1^*]\times[0,t_2^*]$.

Assumption .7. *Assume that there exist t_1^* and t_2^* such that $FDP_\lambda^\infty(t_1^*,0)<q$, $FDP_\lambda^\infty(0,t_2^*)<q$ and $K(t_1^*,t_2^*)>0$.*

Let

$$\widetilde{\mathrm{FDR}}_m=\mathbb{E}\left[\frac{V_m(\widetilde{T}_1^*,\widetilde{T}_2^*)}{V_m(\widetilde{T}_1^*,\widetilde{T}_2^*)+S_m(\widetilde{T}_1^*,\widetilde{T}_2^*)}\right].$$

We show the asymptotic FDR control in the following theorem.

Theorem 1. *Under Assumptions .1-.7, we have*

$$\limsup_m \widetilde{FDR}_m\le q.$$

3.2 Full proof of the theorem

The relationship among the theoretical results is depicted below.

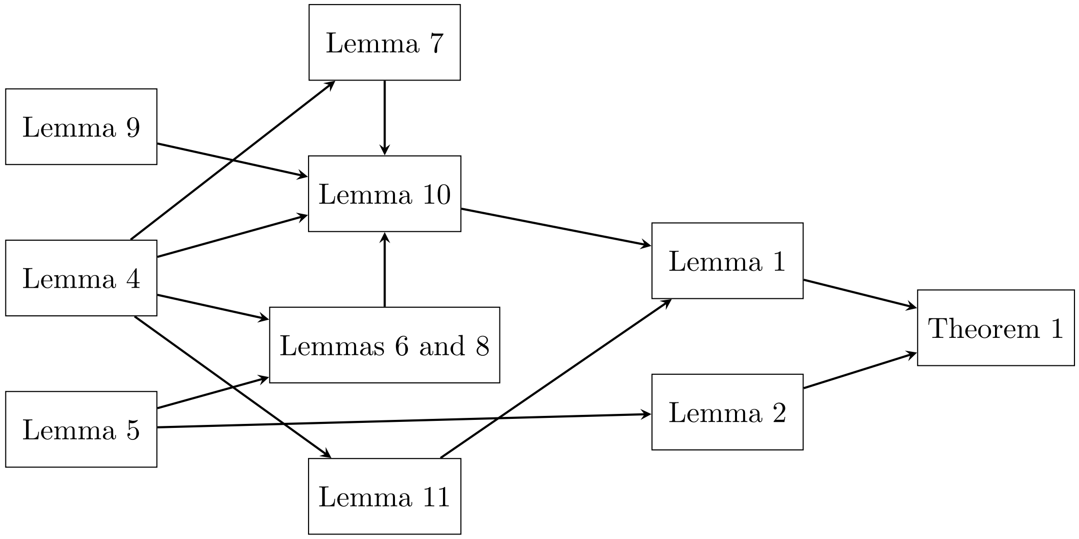

We first introduce some concentration inequalities for a later use.

Lemma 3. *Under Assumptions .3-.4, for* $x_0 > 0$, *we have*

$$\mathbb{P}\left(|\Omega - \mathbb{E}[\Omega]| > x_0\right) \le C_0 \exp\left\{-c_0 n (x_0^2/c_0' \wedge x_0/c_0'')\right\},$$
$$\mathbb{P}\left(\|\Psi - \mathbb{E}[\Psi]\|_{\max} > x_0\right) \le C_1 \exp\left\{-c_1 n (x_0^2/c_1' \wedge x_0/c_1'')\right\},$$
$$\mathbb{P}\left(\|\Gamma - \mathbb{E}[\Gamma]\|_{\max} > x_0\right) \le C_2 \exp\left\{-c_2 n (x_0^2/c_2' \wedge x_0/c_2'')\right\},$$

and, for $0 < x_1 < 3\left(1 \wedge D_0^2/D_2 \wedge D_0^2 E[\Omega]^{-1}\right)$,

$$\mathbb{P}\left(\|\Omega_{\mathbf{Z}|X} - C_{\mathbf{Z}|X}\|_{\max} > x_1\right) \le C_3 \exp\left\{-c_3 n (x_1^2/c_3' \wedge x_1/c_3'')\right\}, \tag{14}$$

and, for $0 < x_2 < L^{-1}\left(3 \wedge 3D_0^2/D_2 \wedge 3D_0^2 E[\Omega]^{-1}\right) \wedge \left(1 \wedge \frac{3w_0^{-1/2}}{(1\wedge D_0^{-1})} \wedge (D_1 d)^{-1}\right) := L'$,

$$\mathbb{P}\left(\|\hat{\boldsymbol{a}} - \boldsymbol{a}\|_{\max} > x_2\right) \le C_4 \exp\left\{-c_4 n (x_2^2/c_4' \wedge x_2/c_4'')\right\}, \tag{15}$$

where $C_l, c_l, c_l', c_l'' > 0$ *denote some absolute constants for* $l = 0, \cdots, 4$ *and, for* $w_0 = \mathbb{E}[\Gamma] C_{\mathbf{Z}|X}^{-1} \mathbb{E}[\Gamma]^\top$,

$$L = \left(\frac{w_0^{3/2}\left(1 \wedge D_0^{-1}\right)}{6\left\{d^2(1+2D_0)D_1 + D_0^2 D_1^2 d^3\right\} + D_1 d w_0^{3/2}\left(1 \wedge D_0^{-1}\right)} \wedge 1\right)$$

and $D_0, D_1, D_2 > 0$ *are some constants such that* $\|\mathbb{E}[\Gamma]\|_{\max} \le D_0$, $\|C_{\mathbf{Z}|X}^{-1}\|_2 \le D_1$ *and* $\mathbb{E}[X_1^2] \ge D_2$. *As a consequence of the above concentration inequalities, we have*

$$\Omega_{\mathbf{Z}|X}^{-1} \overset{a.s.}{\to} C_{\mathbf{Z}|X}^{-1}, \quad \Omega_{X|\mathbf{Z}} \overset{a.s.}{\to} C_{X|\mathbf{Z}}, \quad A \overset{a.s.}{\to} A_0.$$

Proof of Lemma 3. Each element of $\Psi - \mathbb{E}[\Psi]$, $\Gamma - \mathbb{E}[\Gamma]$ and $\Omega - \mathbb{E}[\Omega]$ is sub-exponential. The first three inequalities thus follow from the union bound and the tail bound for sum of sub-exponential random variables, see e.g. Corollary 5.17 of Vershynin (2010). For (14), some algebra gives us

$$\begin{aligned} &\|\Omega_{\mathbf{Z}|X} - C_{\mathbf{Z}|X}\|_{\max} \\ \le &\|\Psi - \mathbb{E}[\Psi]\|_{\max} + \left\{|\Omega^{-1} - \mathbb{E}[\Omega]^{-1}| + D_2^{-1}\right\}\left\{\|\Gamma - \mathbb{E}[\Gamma]\|_{\max}^2 + 2D_0\|\Gamma - \mathbb{E}[\Gamma]\|_{\max}\right\} \\ &+ D_0^2|\Omega^{-1} - \mathbb{E}[\Omega]^{-1}|. \end{aligned} \tag{16}$$

For $0 < x_1 < 3\left(1 \wedge D_0^2/D_2 \wedge D_0^2 E[\Omega]^{-1}\right)$, the following inclusion of events can be verified

$$\begin{aligned} &\left\{\|\Omega_{\mathbf{Z}|X} - C_{\mathbf{Z}|X}\|_{\max} > x_1\right\} \\ \subset &\left\{\|\Psi - \mathbb{E}[\Psi]\|_{\max} > \frac{x_1}{3}\right\} \bigcup \left\{|\Omega^{-1} - \mathbb{E}[\Omega]^{-1}| > \frac{x_1}{3D_0^2}\right\} \bigcup \left\{\|\Gamma - \mathbb{E}[\Gamma]\|_{\max} > \left(\frac{D_2}{2(1+2D_0)} \wedge 1\right)\frac{x_1}{3}\right\} \\ \subset &\left\{\|\Psi - \mathbb{E}[\Psi]\|_{\max} > \frac{x_1}{3}\right\} \bigcup \left\{|\Omega - \mathbb{E}[\Omega]| > \frac{D_2^2 x_1}{6D_0^2}\right\} \bigcup \left\{\|\Gamma - \mathbb{E}[\Gamma]\|_{\max} > \left(\frac{D_2}{2(1+2D_0)} \wedge 1\right)\frac{x_1}{3}\right\}. \end{aligned}$$

The first inclusion follows because, conditional on the events such that

$$\left\{\|\Psi - \mathbb{E}[\Psi]\|_{\max} \le \frac{x_1}{3}\right\} \bigcap \left\{|\Omega^{-1} - \mathbb{E}[\Omega]^{-1}| \le \frac{x_1}{3D_0^2}\right\} \bigcap \left\{\|\Gamma - \mathbb{E}[\Gamma]\|_{\max} \le \left(\frac{D_2}{2(1+2D_0)} \wedge 1\right)\frac{x_1}{3}\right\},$$

(16) implies that

$$\begin{aligned}\|\Omega_{\mathbf{Z}|X} - C_{\mathbf{Z}|X}\|_{\max} &\le \frac{x_1}{3} + \left(\frac{x_1}{3D_0^2} + D_2^{-1}\right)\left\{\left(\frac{D_2}{2(1+2D_0)} \wedge 1\right)^2 \frac{x_1^2}{3^2} + 2D_0\left(\frac{D_2}{2(1+2D_0)} \wedge 1\right)\frac{x_1}{3}\right\} \\ &\quad + D_0^2 \frac{x_1}{3D_0^2} \\ &\le \frac{x_1}{3} + 2D_2^{-1}(1+2D_0)\left(\frac{D_2}{2(1+2D_0)} \wedge 1\right)\frac{x_1}{3} + \frac{x_1}{3} \le x_1.\end{aligned}$$

As $x_1 < 3D_0^2\mathbb{E}[\Omega]^{-1}$ and $\mathbb{E}[\Omega] = \mathbb{E}[X_1^2] \ge D_2$, the second inclusion holds by noticing that

$$\begin{aligned}&\left\{|\Omega^{-1} - \mathbb{E}[\Omega]^{-1}| > \frac{x_1}{3D_0^2}\right\} = \left\{|\Omega - \mathbb{E}[\Omega]| > |\Omega\mathbb{E}[\Omega]|\frac{x_1}{3D_0^2}\right\} \\ &\subset \left\{|\Omega - \mathbb{E}[\Omega]| > |\Omega\mathbb{E}[\Omega]|\frac{x_1}{3D_0^2}, |\Omega\mathbb{E}[\Omega]| > \frac{\mathbb{E}[\Omega]^2}{2}\right\} \bigcup \left\{|\Omega\mathbb{E}[\Omega]| \le \frac{\mathbb{E}[\Omega]^2}{2}\right\} \\ &\subset \left\{|\Omega - \mathbb{E}[\Omega]| > \frac{\mathbb{E}[\Omega]^2 x_1}{6D_0^2}\right\} \bigcup \left\{|\Omega - \mathbb{E}[\Omega]| > \frac{\mathbb{E}[\Omega]}{2}\right\} \subset \left\{|\Omega - \mathbb{E}[\Omega]| > \frac{D_2^2 x_1}{6D_0^2}\right\}.\end{aligned} \tag{17}$$

As for the last inequality, we first observe that

$$\begin{aligned}\|\hat{\boldsymbol{a}} - \boldsymbol{a}\|_{\max} &= \left\|\frac{\Gamma}{\sqrt{w}} - \frac{\mathbb{E}[\Gamma]}{\sqrt{w_0}}\right\|_{\max} \\ &\le \left|\frac{1}{\sqrt{w}} - \frac{1}{\sqrt{w_0}}\right| \{\|\Gamma - \mathbb{E}[\Gamma]\|_{\max} + \|\mathbb{E}[\Gamma]\|_{\max}\} + \frac{1}{\sqrt{w_0}}\|\Gamma - \mathbb{E}[\Gamma]\|_{\max}\end{aligned} \tag{18}$$

where $w = \Gamma\Omega_{\mathbf{Z}|X}^{-1}\Gamma^\top$ and $w_0 = \mathbb{E}[\Gamma]C_{\mathbf{Z}|X}^{-1}\mathbb{E}[\Gamma]^\top$. It also follows that, for $0 < x'' < w_0^{-1/2}$,

$$\begin{aligned}\left\{\left|\frac{1}{\sqrt{w}} - \frac{1}{\sqrt{w_0}}\right| > x''\right\} &\subset \left\{\left|\sqrt{w} - \sqrt{w_0}\right| > \frac{w_0}{2}x''\right\} \\ &= \left\{\left|\frac{\sqrt{w}}{\sqrt{w_0}} - 1\right| > \frac{\sqrt{w_0}}{2}x''\right\} \\ &\subset \left\{\left|\frac{w}{w_0} - 1\right| > \frac{\sqrt{w_0}}{2}x''\right\} = \left\{|w - w_0| > \frac{w_0^{3/2}}{2}x''\right\}.\end{aligned} \tag{19}$$

Combining (18) with (19) implies that, for $0 < x' < \left(3 \wedge \frac{3w_0^{-1/2}}{(1 \wedge D_0^{-1})}\right)$,

$$\begin{aligned}\{\|\hat{\boldsymbol{a}} - \boldsymbol{a}\|_{\max} > x'\} &\subset \left\{\left|\frac{1}{\sqrt{w}} - \frac{1}{\sqrt{w_0}}\right| > \frac{\sqrt{x'}}{\sqrt{3}}\right\} \bigcup \left\{\|\Gamma - \mathbb{E}[\Gamma]\|_{\max} > \frac{\sqrt{x'}}{\sqrt{3}}\right\} \\ &\bigcup \left\{\left|\frac{1}{\sqrt{w}} - \frac{1}{\sqrt{w_0}}\right| \|\mathbb{E}[\Gamma]\|_{\max} > \frac{x'}{3}\right\} \bigcup \left\{\frac{1}{\sqrt{w_0}}\|\Gamma - \mathbb{E}[\Gamma]\|_{\max} > \frac{x'}{3}\right\} \\ &\subset \left\{\left|\frac{1}{\sqrt{w}} - \frac{1}{\sqrt{w_0}}\right| > \frac{x'}{3}\left(1 \wedge D_0^{-1}\right)\right\} \bigcup \left\{\|\Gamma - \mathbb{E}[\Gamma]\|_{\max} > \frac{x'}{3}\left(1 \wedge \sqrt{w_0}\right)\right\} \\ &\subset \left\{|w - w_0| > \frac{x'}{6}w_0^{3/2}\left(1 \wedge D_0^{-1}\right)\right\} \bigcup \left\{\|\Gamma - \mathbb{E}[\Gamma]\|_{\max} > \frac{x'}{3}\left(1 \wedge \sqrt{w_0}\right)\right\}.\end{aligned} \tag{20}$$

Thus, we need to derive the tail bound of $|w - w_0|$. Note that

$$
\begin{aligned}
|w - w_0| &\leq \left|\Gamma\Omega_{\mathbf{Z}|X}^{-1}\Gamma^\top - \mathbb{E}[\Gamma]\Omega_{\mathbf{Z}|X}^{-1}\mathbb{E}[\Gamma]^\top + \mathbb{E}[\Gamma]\Omega_{\mathbf{Z}|X}^{-1}\mathbb{E}[\Gamma]^\top - \mathbb{E}[\Gamma]C_{\mathbf{Z}|X}^{-1}\mathbb{E}[\Gamma]^\top\right| \\
&\leq \underbrace{\left|\Gamma\Omega_{\mathbf{Z}|X}^{-1}\Gamma^\top - \mathbb{E}[\Gamma]\Omega_{\mathbf{Z}|X}^{-1}\mathbb{E}[\Gamma]^\top\right|}_{(*)} + \|\mathbb{E}[\Gamma]\|_2^2\|\Omega_{\mathbf{Z}|X}^{-1} - C_{\mathbf{Z}|X}^{-1}\|_2
\end{aligned}
$$

and

$$
\begin{aligned}
(*) &= \left|(\Gamma - \mathbb{E}[\Gamma])\left(\Omega_{\mathbf{Z}|X}^{-1} - C_{\mathbf{Z}|X}^{-1} + C_{\mathbf{Z}|X}^{-1}\right)(\Gamma - \mathbb{E}[\Gamma] + 2\mathbb{E}[\Gamma])^\top\right| \\
&\leq \left\{\|\Gamma - \mathbb{E}[\Gamma]\|_2^2 + 2\|\Gamma - \mathbb{E}[\Gamma]\|_2\|\mathbb{E}[\Gamma]\|_2\right\}\left\{\|\Omega_{\mathbf{Z}|X}^{-1} - C_{\mathbf{Z}|X}^{-1}\|_2 + \|C_{\mathbf{Z}|X}^{-1}\|_2\right\},
\end{aligned}
$$

which implies that

$$
\begin{aligned}
&|w - w_0| \qquad (21)\\
\leq &d^2\left\{\|\Gamma - \mathbb{E}[\Gamma]\|_{\max}^2 + 2D_0\|\Gamma - \mathbb{E}[\Gamma]\|_{\max}\right\}\left\{\|\Omega_{\mathbf{Z}|X}^{-1} - C_{\mathbf{Z}|X}^{-1}\|_2 + D_1\right\} + D_0^2 d^2\|\Omega_{\mathbf{Z}|X}^{-1} - C_{\mathbf{Z}|X}^{-1}\|_2.
\end{aligned}
$$

We have

$$
\begin{aligned}
\|\Omega_{\mathbf{Z}|X}^{-1} - C_{\mathbf{Z}|X}^{-1}\|_2 &= \|\Omega_{\mathbf{Z}|X}^{-1}(C_{\mathbf{Z}|X} - \Omega_{\mathbf{Z}|X})C_{\mathbf{Z}|X}^{-1}\|_2 \\
&\leq d\|\Omega_{\mathbf{Z}|X} - C_{\mathbf{Z}|X}\|_{\max}\|\Omega_{\mathbf{Z}|X}^{-1}\|_2\|C_{\mathbf{Z}|X}^{-1}\|_2 \qquad (22)\\
&\leq D_1^2 d\|\Omega_{\mathbf{Z}|X} - C_{\mathbf{Z}|X}\|_{\max}\left\{1 - D_1 d\|\Omega_{\mathbf{Z}|X} - C_{\mathbf{Z}|X}\|_{\max}\right\}^{-1}.
\end{aligned}
$$

The last inequality holds when $\|\Omega_{\mathbf{Z}|X} - C_{\mathbf{Z}|X}\|_{\max} < (D_1 d)^{-1}$ due to the fact that

$$
\begin{aligned}
\|\Omega_{\mathbf{Z}|X}^{-1}\|_2 &\leq \|\Omega_{\mathbf{Z}|X}^{-1} - C_{\mathbf{Z}|X}^{-1}\|_2 + \|C_{\mathbf{Z}|X}^{-1}\|_2 \\
&\leq \|\Omega_{\mathbf{Z}|X}^{-1}\|_2\|C_{\mathbf{Z}|X}^{-1}\|_2\|C_{\mathbf{Z}|X} - \Omega_{\mathbf{Z}|X}\|_2 + \|C_{\mathbf{Z}|X}^{-1}\|_2 \\
&\leq D_1 d\|\Omega_{\mathbf{Z}|X}^{-1}\|_2\|\Omega_{\mathbf{Z}|X} - C_{\mathbf{Z}|X}\|_{\max} + \|C_{\mathbf{Z}|X}^{-1}\|_2
\end{aligned}
$$

which is equivalent to $\|\Omega_{\mathbf{Z}|X}^{-1}\|_2\left\{1 - D_1 d\|\Omega_{\mathbf{Z}|X} - C_{\mathbf{Z}|X}\|_{\max}\right\} \leq \|C_{\mathbf{Z}|X}^{-1}\|_2$. For $0 < x < \left(1 \wedge (D_1 d)^{-1}\right)$ where $\kappa = d^2(1 + 2D_0)D_1 + D_0^2 D_1^2 d^3$ and

$$
L = \left\{\frac{w_0^{3/2}\left(1 \wedge D_0^{-1}\right)}{6\kappa + D_1 d w_0^{3/2}\left(1 \wedge D_0^{-1}\right)} \wedge 1\right\},
$$

we have

$$
\left\{\|\Omega_{\mathbf{Z}|X} - C_{\mathbf{Z}|X}\|_{\max} \leq Lx\right\} \cap \left\{\|\Gamma - \mathbb{E}[\Gamma]\|_{\max} \leq Lx\right\} \subset \left\{|w - w_0| \leq \frac{w_0^{3/2}\left(1 \wedge D_0^{-1}\right)}{6}x\right\} \qquad (23)
$$

because, conditional on the two events $\left\{\|\Omega_{\mathbf{Z}|X} - C_{\mathbf{Z}|X}\|_{\max} \leq Lx\right\}$ and $\left\{\|\Gamma - \mathbb{E}[\Gamma]\|_{\max} \leq Lx\right\}$, (21)

and (22) provide

$$
\begin{aligned}
|w - w_0| &\le d^2\left(L^2x^2 + 2D_0Lx\right)\left(\frac{D_1^2 dLx}{1 - D_1dLx} + D_1\right) + D_0^2 d^2 \frac{D_1^2 dLx}{1 - D_1 dLx} \\
&\le d^2(1+2D_0)Lx\left(\frac{D_1}{1 - D_1dLx}\right) + \frac{D_0^2 D_1^2 d^3 Lx}{1 - D_1dLx} = \frac{\kappa Lx}{1 - D_1dLx} \\
&\le \frac{\kappa w_0^{3/2}\left(1 \wedge D_0^{-1}\right)x}{6\kappa + D_1 d w_0^{3/2}\left(1 \wedge D_0^{-1}\right) - D_1 d w_0^{3/2}\left(1 \wedge D_0^{-1}\right)x} \le \frac{w_0^{3/2}\left(1 \wedge D_0^{-1}\right)}{6} x.
\end{aligned}
$$

By (20) and (23), for $0 < x_2 < L^{-1}\left(3 \wedge 3D_0^2/D_2 \wedge 3D_0^2 E[\Omega]^{-1}\right) \wedge \left(1 \wedge \frac{3w_0^{-1/2}}{(1 \wedge D_0^{-1})} \wedge (D_1 d)^{-1}\right)$, we have

$$
\{\|\hat{\boldsymbol{a}} - \boldsymbol{a}\|_{\max} > x_2\} \subset \{\|\Omega_{\mathbf{Z}|X} - C_{\mathbf{Z}|X}\|_{\max} > Lx_2\} \bigcup \left\{\|\Gamma - \mathbb{E}[\Gamma]\|_{\max} > \left(\frac{\left(1 \wedge \sqrt{w_0}\right)}{3} \wedge L\right) x_2\right\},
$$

which completes the proof by applying the union bound together with the tail bounds of $\|\Omega_{\mathbf{Z}|X} - C_{\mathbf{Z}|X}\|_{\max}$ and $\|\Gamma - \mathbb{E}[\Gamma]\|_{\max}$.

A direct implication of the exponential tail bounds is

$$
\Omega \overset{a.s.}{\to} \mathbb{E}[\Omega], \quad \Gamma \overset{a.s.}{\to} \mathbb{E}[\Gamma], \quad \Omega_{\mathbf{Z}|X} \overset{a.s.}{\to} C_{\mathbf{Z}|X} \tag{24}
$$

by the Borel-Cantelli lemma. We next show that

$$
\Omega_{\mathbf{Z}|X}^{-1} \overset{a.s.}{\to} C_{\mathbf{Z}|X}^{-1}, \quad \Omega_{X|\mathbf{Z}} \overset{a.s.}{\to} C_{X|\mathbf{Z}}. \tag{25}
$$

Since $\Omega_{\mathbf{Z}|X} \overset{a.s.}{\to} C_{\mathbf{Z}|X}$, we have $\Omega_{\mathbf{Z}|X}^{-1} \overset{a.s.}{\to} C_{\mathbf{Z}|X}^{-1}$ by (22). Similarly, it can be shown that $\Omega_{X|\mathbf{Z}} \overset{a.s.}{\to} C_{X|\mathbf{Z}}$ under the assumption that $\lambda_{\min}(\mathbb{E}[\Psi]) > 0$. Thus, by the continuous mapping theorem together with (24) and (25), we conclude that $A \overset{a.s.}{\to} A_0$. ♢

The following lemma shows the strong uniform consistency of $\hat{\sigma}_i^2$ and the tail bound for $\hat{\sigma}_i^2/\sigma_i^2$.

Lemma 4. *Under Assumptions .3 and .6,*

$$
\max_{1 \le i \le m}\left|\frac{\hat{\sigma}_i^2}{\sigma_i^2} - 1\right| \overset{a.s.}{\to} 0 \tag{26}
$$

and, for $0 < \delta_1 < (n-d-1)/\log m$,

$$
\mathbb{P}\left(\max_{1 \le i \le m}\left|\frac{\hat{\sigma}_i^2}{\sigma_i^2} - 1\right| > \nu_1\sqrt{\delta_1}\Big|\mathbf{W}\right) \le 2m\exp\{-\delta_1(\log m)\} \tag{27}
$$

where $\nu_1 = 4\sqrt{\log m/(n-d-1)}$.

Proof of Lemma 4. Since $\hat{\sigma}_i^2/\sigma_i^2|\mathbf{W} \sim \chi^2_{n-d-1}/(n-d-1)$, by the tail bound for chi-square random varables as in Lemma 1 of Laurent and Massart (2000), we have

$$
\mathbb{P}\left(\left|\frac{\hat{\sigma}_i^2}{\sigma_i^2} - 1\right| > 2\sqrt{\frac{\delta_0}{n-d-1}} + 2\frac{\delta_0}{n-d-1}\Big|\mathbf{W}\right) \le 2\exp(-\delta_0)
$$

for $\delta_0 > 0$. By the union bound, we have

$$\mathbb{P}\left(\max_{1\le i\le m}\left|\frac{\hat{\sigma}_i^2}{\sigma_i^2}-1\right| > 2\sqrt{\frac{\delta_0}{n-d-1}}+2\frac{\delta_0}{n-d-1}\Big|\mathbf{W}\right) \le 2m\exp(-\delta_0).$$

Letting $\delta_0 = (n-d-1)\delta_0'$ for $0 < \delta_0' < 1$,

$$\mathbb{P}\left(\max_{1\le i\le m}\left|\frac{\hat{\sigma}_i^2}{\sigma_i^2}-1\right| > 2\sqrt{\delta_0'}+2\delta_0'\Big|\mathbf{W}\right) \le \mathbb{P}\left(\max_{1\le i\le m}\left|\frac{\hat{\sigma}_i^2}{\sigma_i^2}-1\right| > 4\delta_0'\Big|\mathbf{W}\right) \le 2m\exp(-(n-d-1)\delta_0').$$

Thus, under Assumptions .3 and .6, (26) follows by the Borel-Cantelli Lemma because

$$\mathbb{P}\left(\max_{1\le i\le m}\left|\frac{\hat{\sigma}_i^2}{\sigma_i^2}-1\right| > 4\delta_0'\right) = \mathbb{E}_{\mathbf{W}}\left[\mathbb{P}\left(\max_{1\le i\le m}\left|\frac{\hat{\sigma}_i^2}{\sigma_i^2}-1\right| > 4\delta_0'\Big|\mathbf{W}\right)\right] \le 2m\exp(-(n-d-1)\delta_0').$$

Choosing $\delta_0 = \delta_1(\log m)$ for $0 < \delta_1 < (n-d-1)/\log m$, we have

$$\begin{aligned}\mathbb{P}\left(\max_{1\le i\le m}\left|\frac{\hat{\sigma}_i^2}{\sigma_i^2}-1\right| \le \nu_1\sqrt{\delta_1}\Big|\mathbf{W}\right) &\ge \mathbb{P}\left(\max_{1\le i\le m}\left|\frac{\hat{\sigma}_i^2}{\sigma_i^2}-1\right| \le 2\left(\frac{\delta_1(\log m)}{n-d-1}+\sqrt{\frac{\delta_1(\log m)}{n-d-1}}\right)\Big|\mathbf{W}\right)\\ &> 1-2m\exp\left\{-\delta_1(\log m)\right\}.\end{aligned}$$

$\diamondsuit$

We next derive the tail bounds for $\max_{1\le i\le m}|\hat{\eta}_i|$ and $\max_{1\le i\le m}|\tilde{\eta}_i|$.

Lemma 5. *Under Assumptions .3-.5, for $0 < \delta_1 < (n-d-1)/(16\log m), \delta_2 > 0$ and x_2 as in Lemma 3, we have*

$$\begin{aligned}&\mathbb{P}\left(\max_{1\le i\le m}|\hat{\eta}_i| > \nu_2(1+\delta_2)\right) \le C_4 m\exp\left\{-c_4 n(x_2^2/c_4'\wedge x_2/c_4'')\right\} + 2m\exp\left\{-\nu_2^2\delta_2^2\right\},\\ &\mathbb{P}\left(\max_{1\le i\le m}|\tilde{\eta}_i| > \nu_2\left(1-\nu_1\sqrt{\delta_1}\right)^{-1}(1+\delta_2)\right)\\ \le& 2m\exp\left\{-\delta_1(\log m)\right\} + C_4 m\exp\left\{-c_4 n(x_2^2/c_4'\wedge x_2/c_4'')\right\} + 2m\exp\left\{-\nu_2^2\delta_2^2\right\},\end{aligned}$$

where $\nu_1 = 4\sqrt{\log m/(n-d-1)}$ and $\nu_2 = (d+\|\boldsymbol{a}\|_1)B(\log m)^{b\vee\frac{1}{2}}$.

Proof of Lemma 5. Note that

$$\mathbb{P}(|\epsilon_i| \le x) \ge 1-2\exp(-x^2) \tag{28}$$

for any $x > 0$ by the well known result about the tail bound of a standard normal random variable. Under model (6), we have

$$\begin{aligned}\mathbb{P}\left(|\hat{\eta}_i| \le \nu_2(1+\delta_2)\right) &\ge \mathbb{P}\left(\{|\eta_i| \le \nu_2\}\bigcap\{|\epsilon_i| \le \nu_2\delta_2\}\right)\\ &\ge \mathbb{P}\left(|\eta_i| \le \nu_2\right) + \mathbb{P}\left(|\epsilon_i| \le \nu_2\delta_2\right) - 1\\ &\ge \mathbb{P}\left(|\eta_i| \le \nu_2, \|\hat{\boldsymbol{a}}-\boldsymbol{a}\|_{\max} \le x_2\right) - 2\exp\left\{-\nu_2^2\delta_2^2\right\}\\ &= \mathbb{P}\left(\|\hat{\boldsymbol{a}}-\boldsymbol{a}\|_{\max} \le x_2\right) - 2\exp\left\{-\nu_2^2\delta_2^2\right\}\\ &\ge 1-C_4\exp\left\{-c_4 n(x_2^2/c_4'\wedge x_2/c_4'')\right\} - 2\exp\left\{-\nu_2^2\delta_2^2\right\}\end{aligned}$$

where the third inequality follows by choosing $x = \nu_2\delta_2$ for $\delta_2 > 0$ in (28) and the equality holds by Lemma 3 because

$$\begin{aligned} |\eta_i| = |\hat{\boldsymbol{a}}^\top \boldsymbol{\xi}_i| \leq \|\hat{\boldsymbol{a}}\|_1 \|\boldsymbol{\xi}_i\|_{\max} &\leq (\|\hat{\boldsymbol{a}} - \boldsymbol{a}\|_1 + \|\boldsymbol{a}\|_1) B(\log m)^b \\ &\leq (d\|\hat{\boldsymbol{a}} - \boldsymbol{a}\|_{\max} + \|\boldsymbol{a}\|_1) B(\log m)^b \\ &\leq (dx_2 + \|\boldsymbol{a}\|_1) B(\log m)^b \leq (d + \|\boldsymbol{a}\|_1) B(\log m)^b = \nu_2 \end{aligned} \tag{29}$$

conditional on the event $\{\|\hat{\boldsymbol{a}} - \boldsymbol{a}\|_{\max} \leq x_2\}$ for x_2 as in Lemma 3 which is smaller than 1. Thus, it follows that

$$\begin{aligned} \mathbb{P}\left(\max_{1\leq i\leq m} |\hat{\eta}_i| \leq \nu_2(1+\delta_2)\right) &= \mathbb{P}\left(\bigcap_{i=1}^m \{|\hat{\eta}_i| \leq \nu_2(1+\delta_2)\}\right) \\ &\geq 1 - C_4 m \exp\left\{-c_4 n(x_2^2/c_4' \wedge x_2/c_4'')\right\} - 2m\exp\left\{-\nu_2^2\delta_2^2\right\}. \end{aligned}$$

For the second inequality, we first observe that

$$\left|\frac{\hat{\sigma}_i}{\sigma_i} - 1\right| \leq \left|\frac{\hat{\sigma}_i^2}{\sigma_i^2} - 1\right| \tag{30}$$

and, provided that $|\hat{\sigma}_i/\sigma_i - 1| < 1$,

$$|\tilde{\eta}_i| = \left|\frac{\sigma_i}{\hat{\sigma}_i} - 1 + 1\right| |\hat{\eta}_i| \leq \left(\frac{|\hat{\sigma}_i/\sigma_i - 1|}{1 - |\hat{\sigma}_i/\sigma_i - 1|}\right)|\hat{\eta}_i| + |\hat{\eta}_i| = \frac{|\hat{\eta}_i|}{1 - |\hat{\sigma}_i/\sigma_i - 1|}.$$

Thus, we have

$$\begin{aligned} &\mathbb{P}\left(\max_{1\leq i\leq m} |\tilde{\eta}_i| \leq \nu_2\left(1 - \nu_1\sqrt{\delta_1}\right)^{-1}(1+\delta_2)\right) \\ \geq &\mathbb{P}\left(\left\{\max_{1\leq i\leq m}\left|\frac{\hat{\sigma}_i}{\sigma_i} - 1\right| \leq \nu_1\sqrt{\delta_1}\right\} \bigcap \left\{\max_{1\leq i\leq m} |\hat{\eta}_i| \leq \nu_2(1+\delta_2)\right\}\right) \\ \geq &\mathbb{P}\left(\left\{\max_{1\leq i\leq m}\left|\frac{\hat{\sigma}_i^2}{\sigma_i^2} - 1\right| \leq \nu_1\sqrt{\delta_1}\right\} \bigcap \left\{\max_{1\leq i\leq m} |\hat{\eta}_i| \leq \nu_2(1+\delta_2)\right\}\right) \\ \geq &1 - 2m\exp\left\{-\delta_1(\log m)\right\} - C_4 m\exp\left\{-c_4 n(x_2^2/c_4' \wedge x_2/c_4'')\right\} - 2m\exp\left\{-\nu_2^2\delta_2^2\right\} \end{aligned}$$

where the last inequality follows due to

$$\begin{aligned} \mathbb{P}\left(\max_{1\leq i\leq m}\left|\frac{\hat{\sigma}_i^2}{\sigma_i^2} - 1\right| \leq \nu_1\sqrt{\delta_1}\right) &= \mathbb{E}_{\mathbf{W}}\left[\mathbb{P}\left(\max_{1\leq i\leq m}\left|\frac{\hat{\sigma}_i^2}{\sigma_i^2} - 1\right| \leq \nu_1\sqrt{\delta_1}\middle|\mathbf{W}\right)\right] \\ &\geq 1 - 2m\exp\left\{-\delta_1(\log m)\right\} \end{aligned} \tag{31}$$

by (27). ◇

We derive the concentration inequalities for $m^{-1}\sum_{i=1}^m \hat{\eta}_i$ and $m^{-1}\sum_{i=1}^m \hat{\eta}_i^2$ in the following lemma.

Lemma 6. *Under Assumptions .3-.5, for $\delta_3 > 0, 0 < \delta_4 < 2^5\nu_3$ and x_2 as in Lemma 3, we have*

$$\mathbb{P}\left(\left|\frac{1}{m}\sum_{i=1}^m \hat{\eta}_i - \mathbb{E}\left[\eta_1\right]\right| > \delta_3\right) \leq 2\exp\left\{-\frac{m\delta_3^2}{2\nu_3}\right\} + C_4\exp\left\{-c_4 n(x_2^2/c_4' \wedge x_2/c_4'')\right\}, \tag{32}$$

and

$$\mathbb{P}\left(\left|\frac{1}{m}\sum_{i=1}^m \hat{\eta}_i^2 - \mathbb{E}\left[\eta_1^2\right] - 1\right| > \delta_4\right) \le 2\exp\left\{-\frac{m\delta_4^2}{2^{11}\nu_3^2}\right\} + 2\exp\left\{-\frac{m\delta_4^2}{32(d+\|\boldsymbol{a}\|_1)^2 B^2 (\log m)^{2b}\nu_3}\right\}$$
$$+ 3C_4 \exp\left\{-c_4 n(x_2^2/c_4' \wedge x_2/c_4'')\right\},$$

where $\nu_3 = 4(d+\|\boldsymbol{a}\|_1)^2 B^2 (\log m)^{2b} + 1$.

Proof of Lemma 6. We have

$$\mathbb{P}\left(\left|\frac{1}{m}\sum_{i=1}^m \hat{\eta}_i - \mathbb{E}\left[\eta_1\right]\right| > \delta_3\right) \le \mathbb{P}\left(\left|\frac{1}{m}\sum_{i=1}^m \hat{\eta}_i - \mathbb{E}\left[\eta_1\right]\right| > \delta_3, \|\hat{\boldsymbol{a}} - \boldsymbol{a}\|_{\max} \le x_2\right)$$
$$+ \mathbb{P}\left(\|\hat{\boldsymbol{a}} - \boldsymbol{a}\|_{\max} > x_2\right).$$

Since the model (6) can be rewritten as

$$\hat{\eta}_i - \mathbb{E}[\eta_1] = \eta_i - \mathbb{E}[\eta_1] + \epsilon_i$$

and, by (29), $|\eta_i - \mathbb{E}[\eta_1]| \le 2(d+\|\boldsymbol{a}\|_1)B(\log m)^b$ conditional on the event $\{\|\hat{\boldsymbol{a}} - \boldsymbol{a}\|_{\max} \le x_2\}$, it can be shown that $(\eta_i - \mathbb{E}[\eta_1])$'s are sub-gaussian with the variance proxy $4(d+\|\boldsymbol{a}\|_1)^2 B^2 (\log m)^{2b}$. Thus, $(\hat{\eta}_i - \mathbb{E}[\eta_1])$'s are also sub-gaussian with the variance proxy ν_3 as ϵ_i's are sub-gaussian with the variance proxy one and η_i's and ϵ_i's are independent. Then, the first inequality follows from Lemma 4 and Corollary 1.7 in Rigollet and Hütter (2015).

For the second inequality, we observe that

$$\begin{aligned}(\hat{\eta}_i - \mathbb{E}[\eta_1])^2 - \mathbb{E}[(\hat{\eta}_i - \mathbb{E}[\eta_1])^2] &= \hat{\eta}_i^2 - \mathbb{E}[\hat{\eta}_1^2] - 2\mathbb{E}[\eta_1](\hat{\eta}_i - \mathbb{E}[\hat{\eta}_1]) \\ &= \hat{\eta}_i^2 - \mathbb{E}[\eta_1^2] - 1 - 2\mathbb{E}[\eta_1](\hat{\eta}_i - \mathbb{E}[\eta_1])\end{aligned}$$

under model (6). By Lemma 1.12 in Rigollet and Hütter (2015), it follows that conditional on the event $\{\|\hat{\boldsymbol{a}} - \boldsymbol{a}\|_{\max} \le x_2\}$,

$$\hat{\eta}_i^2 - \mathbb{E}[\eta_1^2] - 1 - 2\mathbb{E}[\eta_1](\hat{\eta}_i - \mathbb{E}[\eta_1])$$

is sub-exponential with the parameter $16\nu_3$. According to Theorem 1.13 in Rigollet and Hütter (2015),

$$\begin{aligned}&\mathbb{P}\left(\left|\frac{1}{m}\sum_{i=1}^m \hat{\eta}_i^2 - \mathbb{E}[\eta_1^2] - 1 - 2\mathbb{E}[\eta_1]\left(\frac{1}{m}\sum_{i=1}^m \hat{\eta}_i - \mathbb{E}[\eta_1]\right)\right| > \frac{\delta_4}{2}\right) \\ \le& \mathbb{P}\left(\left|\frac{1}{m}\sum_{i=1}^m \hat{\eta}_i^2 - \mathbb{E}[\eta_1^2] - 1 - 2\mathbb{E}[\eta_1]\left(\frac{1}{m}\sum_{i=1}^m \hat{\eta}_i - \mathbb{E}[\eta_1]\right)\right| > \frac{\delta_4}{2}, \|\hat{\boldsymbol{a}} - \boldsymbol{a}\|_{\max} \le x_2\right) \\ &+ \mathbb{P}\left(\|\hat{\boldsymbol{a}} - \boldsymbol{a}\|_{\max} > x_2\right) \\ \le& 2\exp\left\{-\frac{m}{2}\left(\frac{\delta_4^2}{2^{10}\nu_3^2} \wedge \frac{\delta_4}{2^5\nu_3}\right)\right\} + C_4\exp\left\{-c_4 n(x_2^2/c_4' \wedge x_2/c_4'')\right\}\end{aligned}$$

for any $\delta_4 > 0$. We have

$$\begin{aligned}
&\mathbb{P}\left(\left|\frac{1}{m}\sum_{i=1}^m \hat{\eta}_i^2 - \mathbb{E}[\eta_1^2] - 1 - 2\mathbb{E}[\eta_1]\left(\frac{1}{m}\sum_{i=1}^m \hat{\eta}_i - \mathbb{E}[\eta_1]\right)\right| > \frac{\delta_4}{2}\right)\\
\geq &\mathbb{P}\left(\left|\frac{1}{m}\sum_{i=1}^m \hat{\eta}_i^2 - \mathbb{E}[\eta_1^2] - 1\right| > \delta_4\right) + \mathbb{P}\left(\left|2\mathbb{E}[\eta_1]\left(\frac{1}{m}\sum_{i=1}^m \hat{\eta}_i - \mathbb{E}[\eta_1]\right)\right| \leq \frac{\delta_4}{2}\right) - 1\\
\geq &\mathbb{P}\left(\left|\frac{1}{m}\sum_{i=1}^m \hat{\eta}_i^2 - \mathbb{E}[\eta_1^2] - 1\right| > \delta_4\right) + \mathbb{P}\left(\left|\left(\frac{1}{m}\sum_{i=1}^m \hat{\eta}_i - \mathbb{E}[\eta_1]\right)\right| \leq \frac{\delta_4}{4|\mathbb{E}[\eta_1]|}, \|\hat{\boldsymbol{a}} - \boldsymbol{a}\|_{\max} \leq x_2\right) - 1
\end{aligned}$$

and

$$\begin{aligned}
&\mathbb{P}\left(\left|\left(\frac{1}{m}\sum_{i=1}^m \hat{\eta}_i - \mathbb{E}[\eta_1]\right)\right| \leq \frac{\delta_4}{4|\mathbb{E}[\eta_1]|}, \|\hat{\boldsymbol{a}} - \boldsymbol{a}\|_{\max} \leq x_2\right)\\
\geq &\mathbb{P}\left(\left|\left(\frac{1}{m}\sum_{i=1}^m \hat{\eta}_i - \mathbb{E}[\eta_1]\right)\right| \leq \frac{\delta_4}{4(d + \|\boldsymbol{a}\|_1)B(\log m)^b}, \|\hat{\boldsymbol{a}} - \boldsymbol{a}\|_{\max} \leq x_2\right) \quad (\because (29))\\
\geq &1 - 2\exp\left\{-\frac{m\delta_4^2}{32(d + \|\boldsymbol{a}\|_1)^2 B^2 (\log m)^{2b}\nu_3}\right\} - 2C_4 \exp\left\{-c_4 n(x_2^2/c_4' \wedge x_2/c_4'')\right\}.
\end{aligned}$$

The proof is completed by letting $\delta_3 = \delta_4/(4(d + \|\boldsymbol{a}\|_1)B(\log m)^b)$ in (32). ♢

Lemma 7. *For $0 < \delta_1 < (n - d - 1)/(16 \log m)$, $\delta_2, \delta_3 > 0$ and $0 < \delta_4 \leq 2^5\nu_3$ and x_2 as in Lemma 3, under Assumptions .3-.5, we have*

$$\begin{aligned}
&\mathbb{P}\left(\left|\frac{1}{m}\sum_{i=1}^m \log\frac{f_{\widehat{G}_{m,n}}(\hat{\eta}_i)}{f_{\widehat{G}_{m,n}}(\tilde{\eta}_i)}\right| > \frac{\nu_1\sqrt{\delta_1}}{1 - \nu_1\sqrt{\delta_1}}\left(\frac{\mathbb{E}[\eta_1^2] + 1 + \delta_4}{2} + \nu_2(1 + \delta_2)\{|\mathbb{E}[\eta_1]| + \delta_3\}\right)\right)\\
&\leq 2m\exp\left\{-\nu_2^2\delta_2^2\right\} + 2m\exp\left\{-\delta_1(\log m)\right\} + 2\exp\left\{-\frac{m\delta_3^2}{2\nu_3}\right\} + 2\exp\left\{-\frac{m\delta_4^2}{2^{11}\nu_3^2}\right\}\\
&+ 2\exp\left\{-\frac{m\delta_4^2}{32(d + \|\boldsymbol{a}\|_1)^2 B^2(\log m)^{2b}\nu_3}\right\} + (m + 4)C_4\exp\left\{-c_4 n(x_2^2/c_4' \wedge x_2/c_4'')\right\}
\end{aligned}$$

and

$$\begin{aligned}
&\mathbb{P}\left(\left|\frac{1}{m}\sum_{i=1}^m \log\frac{f_{\widetilde{G}_{m,n}}(\hat{\eta}_i)}{f_{\widetilde{G}_{m,n}}(\tilde{\eta}_i)}\right| > \frac{\nu_1\sqrt{\delta_1}}{1 - \nu_1\sqrt{\delta_1}}\left(\frac{\mathbb{E}[\eta_1^2] + 1 + \delta_4}{2} + \frac{\nu_2(1 + \delta_2)}{1 - \nu_1\sqrt{\delta_1}}\{|\mathbb{E}[\eta_1]| + \delta_3\}\right)\right)\\
&\leq 2m\exp\left\{-\nu_2^2\delta_2^2\right\} + 4m\exp\left\{-\delta_1(\log m)\right\} + 2\exp\left\{-\frac{m\delta_3^2}{2\nu_3}\right\} + 2\exp\left\{-\frac{m\delta_4^2}{2^{11}\nu_3^2}\right\}\\
&+ 2\exp\left\{-\frac{m\delta_4^2}{32(d + \|\boldsymbol{a}\|_1)^2 B^2(\log m)^{2b}\nu_3}\right\} + (m + 4)C_4\exp\left\{-c_4 n(x_2^2/c_4' \wedge x_2/c_4'')\right\}
\end{aligned}$$

where $\nu_1 = 4\sqrt{\log m/(n - d - 1)}$, $\nu_2 = (d + \|\boldsymbol{a}\|_1)B(\log m)^{b\vee\frac{1}{2}}$ and $\nu_3 = B^2(\log m)^{2b} + 1$.

Proof of Lemma 7. When $|\hat{\sigma}_i/\sigma_i - 1| < 1$, by (30), we have

$$\left|\sum_{i=1}^m (\tilde{\eta}_i^2 - \hat{\eta}_i^2)\right| \le \max_{1\le i\le m}\left|\frac{\sigma_i^2}{\hat{\sigma}_i^2} - 1\right|\left|\sum_{i=1}^m \hat{\eta}_i^2\right| \le \frac{\max_{1\le i\le m}\left|\frac{\hat{\sigma}_i^2}{\sigma_i^2} - 1\right|}{1 - \max_{1\le i\le m}\left|\frac{\hat{\sigma}_i^2}{\sigma_i^2} - 1\right|}\left|\sum_{i=1}^m \hat{\eta}_i^2\right|,$$

$$\left|\sum_{i=1}^m (\hat{\eta}_i - \tilde{\eta}_i)\right| \le \frac{\max_{1\le i\le m}\left|\frac{\hat{\sigma}_i}{\sigma_i} - 1\right|}{1 - \max_{1\le i\le m}\left|\frac{\hat{\sigma}_i}{\sigma_i} - 1\right|}\left|\sum_{i=1}^m \hat{\eta}_i\right| \le \frac{\max_{1\le i\le m}\left|\frac{\hat{\sigma}_i^2}{\sigma_i^2} - 1\right|}{1 - \max_{1\le i\le m}\left|\frac{\hat{\sigma}_i^2}{\sigma_i^2} - 1\right|}\left|\sum_{i=1}^m \hat{\eta}_i\right|.$$

Recall that $\widehat{G}_{m,n}(u) = \sum_{j=1}^m \hat{\pi}_j \mathbf{1}\{\hat{v}_j \le u\}$ denotes the solution of (7), where $\hat{\pi}_j \ge 0$ with $\sum_{j=1}^m \hat{\pi}_j = 1$ and $\{\hat{v}_1, \cdots, \hat{v}_m\}$ is the set of supportting points for $\widehat{G}_{m,n}$. It follows that

$$\begin{aligned}
&\left|\frac{1}{m}\sum_{i=1}^m \log f_{\widehat{G}_{m,n}}(\hat{\eta}_i) - \frac{1}{m}\sum_{i=1}^m \log f_{\widehat{G}_{m,n}}(\tilde{\eta}_i)\right| = \left|\frac{1}{m}\sum_{i=1}^m \log\left[\frac{\sum_{j=1}^m \phi(\hat{\eta}_i - \hat{v}_j)\hat{\pi}_j}{\sum_{j=1}^m \phi(\tilde{\eta}_i - \hat{v}_j)\hat{\pi}_j}\right]\right| \\
=&\left|\frac{1}{m}\sum_{i=1}^m \log\left[\exp\left\{-(\hat{\eta}_i^2 - \tilde{\eta}_i^2)/2\right\}\frac{\sum_{j=1}^m \exp\left\{\tilde{\eta}_i\hat{v}_j + (\hat{\eta}_i - \tilde{\eta}_i)\hat{v}_j - \hat{v}_j^2/2\right\}\hat{\pi}_j}{\sum_{j=1}^m \exp\left\{\tilde{\eta}_i\hat{v}_j - \hat{v}_j^2/2\right\}\hat{\pi}_j}\right]\right| \\
\le&\left|\frac{1}{m}\sum_{i=1}^m\left(\frac{|\tilde{\eta}_i^2 - \hat{\eta}_i^2|}{2} + |\hat{\eta}_i - \tilde{\eta}_i|\max_{1\le j\le m}|\hat{v}_j|\right)\right| \le \left|\frac{1}{m}\sum_{i=1}^m\left(\frac{|\tilde{\eta}_i^2 - \hat{\eta}_i^2|}{2} + |\hat{\eta}_i - \tilde{\eta}_i|\max_{1\le i\le m}|\hat{\eta}_i|\right)\right| \\
\le&\frac{\max_{1\le i\le m}\left|\frac{\hat{\sigma}_i^2}{\sigma_i^2} - 1\right|}{2\left(1 - \max_{1\le i\le m}\left|\frac{\hat{\sigma}_i^2}{\sigma_i^2} - 1\right|\right)}\left|\frac{1}{m}\sum_{i=1}^m \hat{\eta}_i^2\right| + \max_{1\le i\le m}|\hat{\eta}_i|\frac{\max_{1\le i\le m}\left|\frac{\hat{\sigma}_i^2}{\sigma_i^2} - 1\right|}{1 - \max_{1\le i\le m}\left|\frac{\hat{\sigma}_i^2}{\sigma_i^2} - 1\right|}\left|\frac{1}{m}\sum_{i=1}^m \hat{\eta}_i\right|,
\end{aligned} \tag{33}$$

where the second inequality follows from the fact that the support of $\widehat{G}_{m,n}(u)$ is always within the range of $\hat{\eta}_i$ as noticed in Section 2.3. Let

$$\begin{aligned}
U :=& \frac{\max_{1\le i\le m}\left|\frac{\hat{\sigma}_i^2}{\sigma_i^2} - 1\right|}{2\left(1 - \max_{1\le i\le m}\left|\frac{\hat{\sigma}_i^2}{\sigma_i^2} - 1\right|\right)}\left|\frac{1}{m}\sum_{i=1}^m \hat{\eta}_i^2\right| + \max_{1\le i\le m}|\hat{\eta}_i|\frac{\max_{1\le i\le m}\left|\frac{\hat{\sigma}_i^2}{\sigma_i^2} - 1\right|}{1 - \max_{1\le i\le m}\left|\frac{\hat{\sigma}_i^2}{\sigma_i^2} - 1\right|}\left|\frac{1}{m}\sum_{i=1}^m \hat{\eta}_i\right|, \\
u_{m,n} :=& \frac{\nu_1\sqrt{\delta_1}}{1 - \nu_1\sqrt{\delta_1}}\left(\frac{\mathbb{E}[\eta_1^2] + 1 + \delta_4}{2} + \nu_2(1 + \delta_2)(|\mathbb{E}[\eta_1]| + \delta_3)\right).
\end{aligned}$$

We have the following inclusions of the events

$$\begin{aligned}
&\left\{\max_{1\le i\le m}|\hat{\eta}_i| \le \nu_2(1 + \delta_2)\right\}\bigcap\left\{\max_{1\le i\le m}\left|\frac{\hat{\sigma}_i^2}{\sigma_i^2} - 1\right| \le \nu_1\sqrt{\delta_1}\right\} \\
&\bigcap\left\{\left|\frac{1}{m}\sum_{i=1}^m \hat{\eta}_i - \mathbb{E}\left[\eta_1\right]\right| \le \delta_3\right\}\bigcap\left\{\left|\frac{1}{m}\sum_{i=1}^m \hat{\eta}_i^2 - \mathbb{E}\left[\eta_1^2\right] - 1\right| \le \delta_4\right\} \\
\subset&\left\{\max_{1\le j\le m}|\hat{\eta}_i| \le \nu_2(1 + \delta_2)\right\}\bigcap\left\{\max_{1\le i\le m}\left|\frac{\hat{\sigma}_i^2}{\sigma_i^2} - 1\right| \le \nu_1\sqrt{\delta_1}\right\} \\
&\bigcap\left\{\left|\frac{1}{m}\sum_{i=1}^m \hat{\eta}_i\right| \le |\mathbb{E}[\eta_1]| + \delta_3\right\}\bigcap\left\{\left|\frac{1}{m}\sum_{i=1}^m \hat{\eta}_i^2\right| \le \mathbb{E}[\eta_1^2] + 1 + \delta_4\right\} \subset \left\{U \le u_{m,n}\right\}.
\end{aligned} \tag{34}$$

Thus, by (33)-(34), we have

$$\begin{aligned}
&\mathbb{P}\left(\left|\frac{1}{m}\sum_{i=1}^{m}\log\frac{f_{\widehat{G}_{m,n}}(\hat{\eta}_i)}{f_{\widehat{G}_{m,n}}(\tilde{\eta}_i)}\right|\le u_{m,n}\right)\ge\mathbb{P}\left(U\le u_{m,n}\right)\\
\ge&\mathbb{P}\left(\max_{1\le i\le m}|\hat{\eta}_i|\le\nu_2(1+\delta_2)\right)+\mathbb{P}\left(\max_{1\le i\le m}\left|\frac{\hat{\sigma}_i^2}{\sigma_i^2}-1\right|\le\nu_1\sqrt{\delta_1}\right)\\
&+\mathbb{P}\left(\left|\frac{1}{m}\sum_{i=1}^{m}\hat{\eta}_i-\mathbb{E}\left[\eta_1\right]\right|\le\delta_3\right)+\mathbb{P}\left(\left|\frac{1}{m}\sum_{i=1}^{m}\hat{\eta}_i^2-\mathbb{E}[\eta_1^2]-1\right|\le\delta_4\right)-3,
\end{aligned}$$

which completes the proof of the first inequality by Lemmas 5-6 and (31). Similar argument can be used to verify the second inequality. $\diamondsuit$

We introduce the large deviation inequality for $d_H\left(f_{\widetilde{G}_{m,n}},f_{G_n}\right)$ which can be proved using the arguments for Theorem 1 of Zhang (2009) and (29).

Lemma 8. *Under Assumptions .3-.5 and the event* $\{\|\hat{\boldsymbol{a}}-\boldsymbol{a}\|_{\max}\le x_2\}$ *for* x_2 *as in Lemma 3,* $\widetilde{G}_{m,n}$ *satisfies*

$$\prod_{i=1}^{m}\left\{\frac{f_{\widetilde{G}_{m,n}}(\hat{\eta}_i)}{f_{G_n}(\hat{\eta}_i)}\right\}\ge e^{-2t^2mc_m^2/15}\tag{35}$$

where

$$c_m=\sqrt{\frac{\log(m)}{m}}\left[m^{1/p}\sqrt{\log m}\left\{(d+\|\boldsymbol{a}\|_1)B\vee 1\right\}(\log m)^b\right]^{p/(2+2p)}\tag{36}$$

for some $p>0$*, then there exists an universal constant* t^* *such that for all* $t\ge t^*$ *and* $\log m\ge 4/p$*,*

$$\mathbb{P}\left(d_H\left(f_{\widetilde{G}_{m,n}},f_{G_n}\right)\ge tc_m|\mathbf{W}\right)\le\exp\left\{-\frac{t^2mc_m^2}{2\log m}\right\}\le e^{-t^2\log m}.$$

The following lemma shows that $d_H\left(f_{\widetilde{G}_{m,n}},f_{G_n}\right)=o_{a.s.}(1)$.

Lemma 9. *Under Assumptions .3-.6,* $d_H\left(f_{\widetilde{G}_{m,n}},f_{G_n}\right)=o_{a.s.}(1)$.

Proof of Lemma 9. Define $d_H:=d_H\left(f_{\widetilde{G}_{m,n}},f_{G_n}\right)$ and note that d_H is indexed by both n and m. Since d_H is indexed by only n under Assumption .6, for any $\varepsilon>0$, it suffices to show that $\sum_{n=1}^{\infty}\mathbb{P}\left(d_H\ge\varepsilon\right)<\infty$ by the Borel-Cantelli Lemma.

Under Assumption .6, there exists a $p>0$ such that $(p+1)/(2p)>p_0$. Define c_m as in (36) based on such p. For the same x_2 in Lemma 3 and large enough n, it follows that

$$\mathbb{P}\left(d_H\ge\varepsilon\right)\le\mathbb{P}\left(d_H\ge tc_m\right)\le\mathbb{P}\left(d_H\ge tc_m,\|\hat{\boldsymbol{a}}-\boldsymbol{a}\|_{\max}\le x_2\right)+\mathbb{P}\left(\|\hat{\boldsymbol{a}}-\boldsymbol{a}\|_{\max}>x_2\right)\tag{37}$$

where the first inequality follows because c_m can be made arbitrarily small for large enough n under

Assumption .6. Notice that

$$
\begin{aligned}
&\mathbb{P}\left(d_H \geq tc_m, \|\hat{\boldsymbol{a}}-\boldsymbol{a}\|_{\max} \leq x_2\right) \\
\leq &\mathbb{P}\left(d_H \geq tc_m, \|\hat{\boldsymbol{a}}-\boldsymbol{a}\|_{\max} \leq x_2, \frac{1}{m}\sum_{i=1}^m \log\frac{f_{\widetilde{G}_{m,n}}(\hat{\eta}_i)}{f_{G_n}(\hat{\eta}_i)} \geq -\frac{2t^2c_m^2}{15}\right) \\
+&\mathbb{P}\left(\|\hat{\boldsymbol{a}}-\boldsymbol{a}\|_{\max} \leq x_2, \frac{1}{m}\sum_{i=1}^m \log\frac{f_{\widetilde{G}_{m,n}}(\hat{\eta}_i)}{f_{G_n}(\hat{\eta}_i)} < -\frac{2t^2c_m^2}{15}\right).
\end{aligned}
$$

We shall get an upper bound for each term on the right hand side. Lemma 8 implies that

$$
\begin{aligned}
&\mathbb{P}\left(d_H \geq tc_m, \|\hat{\boldsymbol{a}}-\boldsymbol{a}\|_{\max} \leq x_2, \frac{1}{m}\sum_{i=1}^m \log\frac{f_{\widetilde{G}_{m,n}}(\hat{\eta}_i)}{f_{G_n}(\hat{\eta}_i)} \geq -\frac{2t^2c_m^2}{15}\right) \\
=&\mathbb{E}_{\mathbf{W}}\left[\mathbb{P}\left(d_H \geq tc_m, \frac{1}{m}\sum_{i=1}^m \log\frac{f_{\widetilde{G}_{m,n}}(\hat{\eta}_i)}{f_{G_n}(\hat{\eta}_i)} \geq -\frac{2t^2c_m^2}{15}\Big|\mathbf{W}\right)\mathbf{1}\left\{\|\hat{\boldsymbol{a}}-\boldsymbol{a}\|_{\max} \leq x_2\right\}\right] \leq D_3 m^{-2}
\end{aligned} \tag{38}
$$

for some constant $D_3 > 0$ and large enough t. Moreover, we have

$$
\begin{aligned}
&\mathbb{P}\left(\frac{1}{m}\sum_{i=1}^m \log\frac{f_{\widetilde{G}_{m,n}}(\hat{\eta}_i)}{f_{G_n}(\hat{\eta}_i)} < -\frac{2t^2c_m^2}{15}, \|\hat{\boldsymbol{a}}-\boldsymbol{a}\|_{\max} \leq x_2\right) \\
=&\mathbb{P}\Bigg(\frac{1}{m}\sum_{i=1}^m \log\frac{f_{\widehat{G}_{m,n}}(\hat{\eta}_i)}{f_{G_n}(\hat{\eta}_i)} - \frac{1}{m}\sum_{i=1}^m \log\frac{f_{\widehat{G}_{m,n}}(\hat{\eta}_i)}{f_{\widehat{G}_{m,n}}(\tilde{\eta}_i)} - \frac{1}{m}\sum_{i=1}^m \log\frac{f_{\widetilde{G}_{m,n}}(\tilde{\eta}_i)}{f_{\widetilde{G}_{m,n}}(\hat{\eta}_i)} \\
&+\frac{1}{m}\sum_{i=1}^m \log\frac{f_{\widetilde{G}_{m,n}}(\tilde{\eta}_i)}{f_{\widehat{G}_{m,n}}(\tilde{\eta}_i)} < -\frac{2t^2c_m^2}{15}, \|\hat{\boldsymbol{a}}-\boldsymbol{a}\|_{\max} \leq x_2\Bigg) \\
\leq&\mathbb{P}\left(\frac{2t^2c_m^2}{15} < \left|\frac{1}{m}\sum_{i=1}^m \log\frac{f_{\widehat{G}_{m,n}}(\hat{\eta}_i)}{f_{\widehat{G}_{m,n}}(\tilde{\eta}_i)}\right| + \left|\frac{1}{m}\sum_{i=1}^m \log\frac{f_{\widetilde{G}_{m,n}}(\tilde{\eta}_i)}{f_{\widetilde{G}_{m,n}}(\hat{\eta}_i)}\right|, \|\hat{\boldsymbol{a}}-\boldsymbol{a}\|_{\max} \leq x_2\right) \\
\leq&\mathbb{P}\left(2u_{m,n} < \left|\frac{1}{m}\sum_{i=1}^m \log\frac{f_{\widehat{G}_{m,n}}(\hat{\eta}_i)}{f_{\widehat{G}_{m,n}}(\tilde{\eta}_i)}\right| + \left|\frac{1}{m}\sum_{i=1}^m \log\frac{f_{\widetilde{G}_{m,n}}(\tilde{\eta}_i)}{f_{\widetilde{G}_{m,n}}(\hat{\eta}_i)}\right|, \|\hat{\boldsymbol{a}}-\boldsymbol{a}\|_{\max} \leq x_2\right) \\
\leq&\mathbb{P}\left(u_{m,n} < \left|\frac{1}{m}\sum_{i=1}^m \log\frac{f_{\widehat{G}_{m,n}}(\hat{\eta}_i)}{f_{\widehat{G}_{m,n}}(\tilde{\eta}_i)}\right|\right) + \mathbb{P}\left(u_{m,n} < \left|\frac{1}{m}\sum_{i=1}^m \log\frac{f_{\widehat{G}_{m,n}}(\hat{\eta}_i)}{f_{\widehat{G}_{m,n}}(\tilde{\eta}_i)}\right|\right)
\end{aligned} \tag{39}
$$

where the second inequality follows due to the fact that, conditional on the event $\{\|\hat{\boldsymbol{a}}-\boldsymbol{a}\|_{\max} \leq x_2\}$,

$$
u_{m,n} = \frac{\nu_1\sqrt{\delta_1}}{1-\nu_1\sqrt{\delta_1}}\left(\frac{\mathbb{E}[\eta_1^2]+1+\delta_4}{2} + \nu_2(1+\delta_2)(|\mathbb{E}[\eta_1]|+\delta_3)\right) = O\left(\frac{(\log m)^{b+\frac{1}{2}+(b\vee\frac{1}{2})}}{\sqrt{n}}\right)
$$

by (29) and

$$
\frac{2t^2c_m^2}{15} \geq \frac{2t^2}{15}\frac{1}{m^{p/(1+p)}} \geq \frac{2t^2}{15D_5}\frac{(\log m)^{b+\frac{1}{2}+(b\vee\frac{1}{2})}}{\sqrt{n}} \geq \frac{2t^2}{15D_4D_5}u_{m,n} \geq 2u_{m,n} \tag{40}
$$

for large enough n and $t \geq (t^* \vee \sqrt{15D_4D_5})$, where $D_4, D_5 \geq 0$ denote some constants such that

$u_{m,n} \leq D_4 \frac{(\log m)^{b+\frac{1}{2}+(b\vee\frac{1}{2})}}{\sqrt{n}}$ and

$$m^{p/(1+p)} = m^{1/(2p_0)-\epsilon} \leq D_5 \frac{\sqrt{n}}{(\log m)^{b+\frac{1}{2}+(b\vee\frac{1}{2})}}$$

for $\epsilon = 1/(2p_0) - p/(1+p) > 0$ under Assumptions .5-.6. Thus, for large enough n, combining (37) together with (38) and (39) implies that

$$\begin{aligned}
&\mathbb{P}\left(d_H \geq \varepsilon\right) \\
&\leq D_3 m^{-2} + 4m \exp\left\{-\nu_2^2\delta_2^2\right\} + 6m \exp\left\{-\delta_1(\log m)\right\} + 4\exp\left\{-\frac{m\delta_3^2}{2\nu_3}\right\} + 4\exp\left\{-\frac{m\delta_4^2}{2^{11}\nu_3^2}\right\} \\
&\quad + 4\exp\left\{-\frac{m\delta_4^2}{32(d+\|\boldsymbol{a}\|_1)^2 B^2 (\log m)^{2b}\nu_3}\right\} + (2m+9)C_4 \exp\left\{-c_4 n(x_2^2/c_4' \wedge x_2/c_4'')\right\}
\end{aligned}$$

by Lemma 7. Then, under Assumption .6, each infinite series with respect to n on the right hand side can be shown to be finite with the choice of $\delta_1 > 2$ and $\delta_2 > \sqrt{2}/((d+\|\boldsymbol{a}\|_1)B)$. This implies $\sum_{n=1}^{\infty} \mathbb{P}\left(d_H \geq \varepsilon\right) < \infty$, which completes the proof. ♢

We next show $d_H\left(f_{G_n}, f_{G_0}\right) = o_{a.s.}(1)$ in the following lemma.

Lemma 10. *Under Assumptions .3-.6, we have* $d_H\left(f_{G_n}, f_{G_0}\right) = o_{a.s.}(1)$.

Proof of Lemma 10. We first note that $d_H^2\left(f_{G_n}, f_{G_0}\right) \leq d_{TV}\left(f_{G_n}, f_{G_0}\right)$ where d_{TV} denotes the total variation distance such that

$$d_{TV}\left(f_{G_n}, f_{G_0}\right) = \frac{1}{2}\int\left|\int \phi(x-y)\left\{dP(\hat{\mathbf{a}}^\top\boldsymbol{\xi}_i \leq y|\mathbf{W}) - dP(\mathbf{a}^\top\boldsymbol{\xi}_i \leq y)\right\}\right| dx.$$

The main idea of the proof is to find an upper bound of $d_{TV}\left(f_{G_n}, f_{G_0}\right)$ in terms of $\|\hat{\mathbf{a}} - \mathbf{a}\|_{\max}$.

Let $\mathbf{a} = (a_1, \cdots, a_d)^\top$. Without loss of generality, we suppose the first l elements of $\boldsymbol{a}$ are zero and the rest is non-zero for some $0 \leq l \leq d$. Then, the density of $\mathbf{a}^\top\boldsymbol{\xi}$ can be written as

$$f_{\mathbf{a}^\top\boldsymbol{\xi}}(y) = \begin{cases} \frac{1}{|a_d|}\int h\left(z_1, \cdots, z_{d-1}, \frac{y-\sum_{i=l+1}^{d-1} a_i z_i}{a_d}\right) dz_1\cdots dz_{d-1}, & \text{for } l < d, \\ \delta_{y=0}, & \text{for } l = d, \end{cases}$$

where $\sum_{i=l+1}^{d-1} a_i z_i$ is defined to be 0 if $l = d-1$. We prove the result when $l < d$ as it is straightforward if $l = d$. When $l < d$, the joint density of $(a_1\xi_1, \cdots, a_d\xi_d)$ is

$$g_{\boldsymbol{a}}(\boldsymbol{z}) = \left(\prod_{i=1}^{l} \delta_{z_i=0}\right) \frac{1}{\prod_{i=l+1}^{d}|a_i|} h_{-l}\left(\frac{z_{l+1}}{a_{l+1}}, \cdots, \frac{z_d}{a_d}\right)$$

where $\prod_{i=1}^{l}\delta_{z_i=0}$ is defined to be 1 if $l = 0$ and h_{-l} denotes the density of $(\xi_{l+1}, \cdots, \xi_d)$. Then, the

density of $\boldsymbol{a}^\top \boldsymbol{\xi}$ is

$$\begin{aligned}
f_{\boldsymbol{a}^\top \boldsymbol{\xi}}(y) &= \frac{1}{\prod_{i=l+1}^{d} |a_i|} \int h_{-l}\left(\frac{z_{l+1}}{a_{l+1}}, \cdots, \frac{y - \sum_{i=l+1}^{d-1} z_i}{a_d}\right) dz_{l+1} \cdots dz_{d-1} \\
&= \frac{1}{|a_d|} \int h_{-l}\left(z_{l+1}, \cdots, z_{d-1}, \frac{y - \sum_{i=l+1}^{d-1} a_i z_i}{a_d}\right) dz_{l+1} \cdots dz_{d-1} \\
&= \frac{1}{|a_d|} \int h\left(z_1, \cdots, z_{d-1}, \frac{y - \sum_{i=l+1}^{d-1} a_i z_i}{a_d}\right) dz_1 \cdots dz_{d-1}.
\end{aligned}$$

Suppose the first k elements of $\hat{\boldsymbol{a}}$ are zero and the rest are non-zero for some $0 \leq k \leq d$. Similarly, the density of $\hat{\mathbf{a}}^\top \boldsymbol{\xi}$ is given by

$$f_{\hat{\mathbf{a}}^\top \boldsymbol{\xi}}(y) = \begin{cases} \frac{1}{|\hat{a}_d|} \int h\left(z_1, \cdots, z_{d-1}, \frac{y - \sum_{i=k+1}^{d-1} \hat{a}_i z_i}{\hat{a}_d}\right) dz_1 \cdots dz_{d-1}, & \text{for } k < d, \\ \delta_{y=0}, & \text{for } k = d. \end{cases}$$

When $l < d$ and $k < d$, we have

$$\begin{aligned}
d_{TV}\left(f_{G_n}, f_{G_0}\right) =& \frac{1}{2} \int \left| \int \frac{\phi(x-y)}{|\hat{a}_d|} \int h\left(z_1, \cdots, z_{d-1}, \frac{y - \sum_{i=k+1}^{d-1} \hat{a}_i z_i}{\hat{a}_d}\right) dz_1 \cdots dz_{d-1} dy \right. \\
& \left. - \int \frac{\phi(x-y)}{|a_d|} \int h\left(z_1, \cdots, z_{d-1}, \frac{y - \sum_{i=l+1}^{d-1} a_i z_i}{a_d}\right) dz_1 \cdots dz_{d-1} dy \right| dx \\
\leq& \frac{1}{2} \int \left| \int \phi(x - \hat{\mathbf{a}}^\top \mathbf{z}) h(\mathbf{z}) d\mathbf{z} - \phi(x - \mathbf{a}^\top \mathbf{z}) h(\mathbf{z}) d\mathbf{z} \right| dx \\
\leq& \frac{1}{2} \int \int \left| \phi(x - \hat{\mathbf{a}}^\top \mathbf{z}) - \phi(x - \mathbf{a}^\top \mathbf{z}) \right| dx h(\mathbf{z}) d\mathbf{z} \\
\leq& \frac{1}{2} \int \left\{ \exp\left\{ |(\hat{\mathbf{a}} - \mathbf{a})^\top \mathbf{z}|^2 \right\} - 1 \right\}^{1/2} h(\mathbf{z}) d\mathbf{z} \\
\leq& \frac{1}{\sqrt{2}} \int \sqrt{|(\hat{\mathbf{a}} - \mathbf{a})^\top \mathbf{z}|^2} h(\mathbf{z}) d\mathbf{z} \leq \frac{dB(\log m)^b \|\hat{\mathbf{a}} - \mathbf{a}\|_{\max}}{\sqrt{2}}
\end{aligned}$$

under the event that $\|\hat{\mathbf{a}} - \mathbf{a}\|_{\max} \leq (dB(\log m)^b)^{-1} \varepsilon$ for $0 < \varepsilon < 1$ because $e^x \leq 1 + 2x$ for any $0 < x < 1$ and

$$|(\hat{\mathbf{a}} - \mathbf{a})^\top \mathbf{z}|^2 \leq \|\hat{\mathbf{a}} - \mathbf{a}\|_{\max}^2 \|\mathbf{z}\|_1^2 \leq (dB(\log m)^b)^2 \|\hat{\mathbf{a}} - \mathbf{a}\|_{\max}^2$$

under Assumption .5. The same bound holds if $l = d$ or $k = d$. Then, for $0 < \varepsilon < \sqrt{2} dB(\log m)^b L'$ where L' is the same as in Lemma 3, it follows that

$$\begin{aligned}
& \left\{ d_H^2(f_{G_n}, f_{G_0}) > \varepsilon/2 \right\} \\
\subset & \left\{ d_{TV}(f_{G_n}, f_{G_0}) > \varepsilon/2, \|\hat{\mathbf{a}} - \mathbf{a}\|_{\max} \leq (dB(\log m)^b)^{-1} \varepsilon \right\} \cup \left\{ \|\hat{\mathbf{a}} - \mathbf{a}\|_{\max} > (dB(\log m)^b)^{-1} \varepsilon \right\} \\
\subset & \left\{ \frac{dB(\log m)^b \|\hat{\mathbf{a}} - \mathbf{a}\|_{\max}}{\sqrt{2}} > \varepsilon/2, \|\hat{\mathbf{a}} - \mathbf{a}\|_{\max} \leq (dB(\log m)^b)^{-1} \varepsilon \right\} \cup \left\{ \|\hat{\mathbf{a}} - \mathbf{a}\|_{\max} > (dB(\log m)^b)^{-1} \varepsilon \right\} \\
\subset & \left\{ \|\hat{\mathbf{a}} - \mathbf{a}\|_{\max} > (dB(\log m)^b)^{-1} \varepsilon / \sqrt{2} \right\}.
\end{aligned}$$

Under Assumption .6, for large enough n, we have

$$\sum_{n=1}^{\infty} \mathbb{P}\left(d_H^2(f_{G_n}, f_{G_0}) > \varepsilon/2\right) \leq \sum_{n=1}^{\infty} \mathbb{P}\left(\|\hat{\mathbf{a}} - \mathbf{a}\|_{\max} > (dB(\log m)^b)^{-1}\varepsilon/\sqrt{2}\right)$$
$$\leq \sum_{n=1}^{\infty} \mathbb{P}\left(\|\hat{\mathbf{a}} - \mathbf{a}\|_{\max} > \frac{\varepsilon}{\sqrt{2}dBD_6(\log n)^b}\right),$$

where $D_6 > 0$ is some constant such that $(\log m)^b \leq D_6(\log n)^b$ for large enough n which must exist under Assumption .6. By Lemma 3, the summation above is finite. Thus we conclude that $d_H(f_{G_n}, f_{G_0}) = o_{a.s.}(1)$ by the Borel-Cantelli lemma. ♢

Now we provide the proofs of Lemmas 1 and 2.

Proof of Lemma 1. Note that

$$\left|\int L(Ax, t_1, t_2)d\widetilde{G}_{m,n}(x) - \int L_0(A_0x, t_1, t_2)dG_0(x)\right|$$
$$\leq \left|\int \{L(Ax, t_1, t_2) - L_0(A_0x, t_1, t_2)\}\, d\widetilde{G}_{m,n}(x)\right| + \left|\int L_0(A_0x, t_1, t_2)(d\widetilde{G}_{m,n}(x) - dG_0(x))\right|.$$

It thus suffices to show that

$$\sup_{t_1 \leq t_1', t_2 \leq t_2', x \in \mathbb{R}} |L(Ax, t_1, t_2) - L_0(A_0x, t_1, t_2)| \overset{a.s.}{\to} 0, \tag{41}$$

$$\sup_{t_1 \leq t_1', t_2 \leq t_2'} \left|\int L_0(A_0x, t_1, t_2)(d\widetilde{G}_{m,n}(x) - dG_0(x))\right| \overset{a.s.}{\to} 0. \tag{42}$$

We first verify the pointwise convergence in (41)-(42) and proceed to show the uniform convergence as stated. As $\widetilde{G}_{m,n} = \widetilde{G}_{m(n),n}$ under Assumption .6, throughout the proof, we denote $\widetilde{G}_{m,n}$ by $\widetilde{G}_n$ for notational simplicity.

1. **Pointwise convergence**
   Let $a_n = a_n(t_1, t_2) = \left|\int L_0(A_0x, t_1, t_2)(d\widetilde{G}_n(x) - dG_0(x))\right|$ under Assumption .6. By Lemmas 9 and 10,

$$d_H(f_{\widetilde{G}_n}(x), f_{G_0}(x)) \leq d_H(f_{\widetilde{G}_n}(x), f_{G_n}(x)) + d_H(f_{G_n}(x), f_{G_0}(x)) = o_{a.s.}(1). \tag{43}$$

   Since $\int |f(x) - g(x)|dx \leq 2d_H(f, g)\sqrt{2 - d_H^2(f, g)}$ and $f_G(x) \leq 1/\sqrt{2\pi}$ for any G, we have

$$\int |f_{\widetilde{G}_n}(x) - f_{G_0}(x)|^2 dx \leq \frac{2}{\sqrt{2\pi}} \int |f_{\widetilde{G}_n}(x) - f_{G_0}(x)|dx = o_{a.s.}(1).$$

   For a density function or distribution function f, denote by f^* its Fourier transformation. By the Parseval's identity, we have

$$\int |f_{\widetilde{G}_n}(x) - f_{G_0}(x)|^2 dx = \int \left|\phi^*(t)\widetilde{G}_n^*(t) - \phi^*(t)G_0^*(t)\right|^2 dt$$
$$= \int \phi^*(t)^2 \left|\widetilde{G}_n^*(t) - G_0^*(t)\right|^2 dt = o_{a.s.}(1).$$

Consider any convergent subsequence of a_n, say a_{n_j}. As $\int \phi^*(t)^2 \left|\widehat{G}^*_{n_j}(t) - G^*_0(t)\right|^2 dt = o_{a.s.}(1)$, there exists a further subsequence $\widehat{G}^*_{n_{j_k}}(t)$ such that

$$\widehat{G}^*_{n_{j_k}}(t) \overset{a.s.}{\to} G^*_0(t)$$

for almost every t with respect to the measure $\phi^*(t)dt$ (and thus also with respect to the Lebesgue measure). By the continuity theorem, $\widehat{G}_{n_{j_k}}(t) \overset{a.s.}{\to} G_0(t)$ for any continuous point t of $G_0(t)$. Then, we have $a_{n_{j_k}} \to 0$ by the Portmanteau theorem. As a_{n_j} is convergent, it must converge to zero as well. Since a_{n_j} is an arbitrary convergent subsequence, we have

$$a_n = \left| \int L_0(A_0x, t_1, t_2)(d\widetilde{G}_n(x) - dG_0(x)) \right| \overset{a.s.}{\to} 0 \tag{44}$$

for any t_1, t_2. To show that $|L(Ax, t_1, t_2) - L_0(A_0x, t_1, t_2)| \overset{a.s.}{\to} 0$ for given x, t_1 and t_2, we note that the covariance matrix of (V_1, V_2) converges almost surely to the covariance matrix of $(\breve{V}_1, \breve{V}_2)$ by Lemma 3. It follows from the continuous mapping theorem that

$$|L(Ax, t_1, t_2) - L_0(A_0x, t_1, t_2)| \overset{a.s.}{\to} 0.$$

2. **Uniform convergence**
We first show (41). For any $\delta > 0$, we can choose a large enough $M' = M'(\delta)$ such that

$$\int_{|x|>M'} dG_0(x) < \delta. \tag{45}$$

For such M', using similar argument as above, we can verify that

$$\left| \int_{|x|>M'} (d\widetilde{G}_n(x) - dG_0(x)) \right| \overset{a.s.}{\to} 0. \tag{46}$$

We partition the rectangular region $[0, t'_1] \times [0, t'_2]$ into finite disjoint sets $\cup_{1 \le j \le B_1} \mathcal{V}_j$ such that uniformly over j,

$$\sup_{(t_1,t_2),(\tilde{t}_1,\tilde{t}_2) \in \mathcal{V}_j} \left| \int (L_0(A_0x, t_1, t_2) - L_0(A_0x, \tilde{t}_1, \tilde{t}_2)) dG_0(x) \right| < \delta, \tag{47}$$

and also for $|x| \le M'$

$$\sup_{(t_1,t_2),(\tilde{t}_1,\tilde{t}_2) \in \mathcal{V}_j} \left| L_0(A_0x, t_1, t_2) - L_0(A_0x, \tilde{t}_1, \tilde{t}_2) \right| < \delta. \tag{48}$$

We have for large enough m and uniformly over j,

$$\begin{aligned}
&\sup_{(t_1,t_2),(\tilde t_1,\tilde t_2)\in\mathcal{V}_j}\left|\int (L_0(A_0x,t_1,t_2)-L_0(A_0x,\tilde t_1,\tilde t_2))d\widetilde{G}_n(x)\right|\\
&\le \sup_{(t_1,t_2),(\tilde t_1,\tilde t_2)\in\mathcal{V}_j}\left|\int_{|x|\le M'} (L_0(A_0x,t_1,t_2)-L_0(A_0x,\tilde t_1,\tilde t_2))d\widetilde{G}_n(x)\right|\\
&+ \sup_{(t_1,t_2),(\tilde t_1,\tilde t_2)\in\mathcal{V}_j}\left|\int_{|x|> M'} (L_0(A_0x,t_1,t_2)-L_0(A_0x,\tilde t_1,\tilde t_2))d\widetilde{G}_n(x)\right| \qquad (49)\\
&\le \sup_{(t_1,t_2),(\tilde t_1,\tilde t_2)\in\mathcal{V}_j}\left|\int_{|x|\le M'} (L_0(A_0x,t_1,t_2)-L_0(A_0x,\tilde t_1,\tilde t_2))d\widetilde{G}_n(x)\right|\\
&+2\left|\int_{|x|>M'}(d\widetilde{G}_n(x)-dG_0(x)+dG_0(x))\right|\le 5\delta
\end{aligned}$$

almost surely from (45),(46) and (48). Choosing $(t_1^j,t_2^j)\in\mathcal{V}_j$ for $1\le j\le B_1$, we have

$$\begin{aligned}
&\sup_{t_1\le t_1',t_2\le t_2'}\left|\int L_0(A_0x,t_1,t_2)(d\widetilde{G}_n(x)-dG_0(x))\right|\\
&=\sup_{(t_1,t_2)\in\cup_{1\le j\le B}\mathcal{V}_j}\left|\int L_0(A_0x,t_1,t_2)(d\widetilde{G}_n(x)-dG_0(x))\right|\\
&=\max_{1\le j\le B}\sup_{(t_1,t_2)\in\mathcal{V}_j}\left|\int \left\{L_0(A_0x,t_1,t_2)-L_0(A_0x,t_1^j,t_2^j)+L_0(A_0x,t_1^j,t_2^j)\right\}(d\widetilde{G}_n(x)-dG_0(x))\right|\\
&\le 6\delta+\max_{1\le j\le B_1}\left|\int L_0(A_0x,t_1^j,t_2^j)(d\widetilde{G}_n(x)-dG_0(x))\right|
\end{aligned}$$

by (47) and (49). The proof for (41) is completed in view of (44).

To show (42), by the triangle inequality, it suffices to show that

$$\sup_{t_1\le t_1',t_2\le t_2',x\in\mathbb{R}}|L(Ax,t_1,t_2)-L_0(Ax,t_1,t_2)|\overset{a.s.}{\to}0, \qquad (50)$$

$$\sup_{t_1\le t_1',t_2\le t_2',x\in\mathbb{R}}|L_0(Ax,t_1,t_2)-L_0(A_0x,t_1,t_2)|\overset{a.s.}{\to}0. \qquad (51)$$

Then, (50) follows by applying Scheffe's Lemma together with the pointwise almost sure convergence of the probability density of (V_1,V_2) to that of $(\check V_1,\check V_2)$ by Lemma 3. We next show (51). We first observe that, for any $a,t_1,t_2\in\mathbb{R}$,

$$\begin{aligned}
L_0(a,t_1,t_2)&=\mathbb{P}(a+\check V_1\ge t_1,\check V_2\ge t_2)+\mathbb{P}(a+\check V_1\le -t_1,\check V_2\ge t_2)\\
&+\mathbb{P}(a+\check V_1\ge t_1,\check V_2\le -t_2)+\mathbb{P}(a+\check V_1\le -t_1,\check V_2\le -t_2)\\
&=\{\Phi(-t_2)-\Phi(t_1-a)+\Psi_1(t_1-a,t_2)\} \qquad (52)\\
&+\{\Phi(-t_1-a)-\Psi_1(-t_1-a,t_2)\}+\{\Phi(-t_2)-\Psi_1(t_1-a,-t_2)\}\\
&+\Psi_1(-t_1-a,-t_2),
\end{aligned}$$

where $\Psi_1(t_1,t_2)=\mathbb{P}(\check V_1\le t_1,\check V_2\le t_2)$. For any $\delta>0$, we can choose some large enough

$M''(\delta, t_1', t_2', A_0) > 0$ such that

$$\begin{aligned}&\left\{\Phi(t_1' - A_0M'') \vee \Psi_1(t_1' - A_0M'', t_2')\right\} \leq \delta,\\&\left\{\Phi(-t_1' + A_0M'') \wedge \Psi_1(-t_1' + A_0M'', -t_2')\right\} \geq 1 - \delta\end{aligned} \tag{53}$$

which implies that for any $x \geq M''$ and $t_1 \leq t_1', t_2 \leq t_2'$,

$$\begin{aligned}&\Phi(-t_1 - A_0x) \leq \delta,\\&\Psi_1(-t_1 - A_0x, -t_2) \leq \delta,\\&\Psi_1(t_1 - A_0x, -t_2) \leq \delta,\end{aligned} \tag{54}$$

and, for any $x \leq -M''$,

$$\begin{aligned}&1 - \delta \leq \Phi(t_1 - A_0x),\\&1 - \delta \leq \Psi_1(-t_1 - A_0x, t_2),\\&1 - \delta \leq \Psi_1(t_1 - A_0x, t_2).\end{aligned} \tag{55}$$

Here we use the fact that Φ and Ψ_1 are non-decreasing continuous functions. Thus, to show (51), it suffices to show that

$$\sup_{t_1 \leq t_1', t_2 \leq t_2', |x| \geq M''} |L_0(Ax, t_1, t_2) - L_0(A_0x, t_1, t_2)| \overset{a.s.}{\to} 0, \tag{56}$$

$$\sup_{t_1 \leq t_1', t_2 \leq t_2', |x| < M''} |L_0(Ax, t_1, t_2) - L_0(A_0x, t_1, t_2)| \overset{a.s.}{\to} 0. \tag{57}$$

By (52),

$$\begin{aligned}&\sup_{t_1 \leq t_1', t_2 \leq t_2', |x| \geq M''} |L_0(Ax, t_1, t_2) - L_0(A_0x, t_1, t_2)|\\
\leq &\sup_{t_1 \leq t_1', t_2 \leq t_2', |x| \geq M''} |\Phi(-t_1 - Ax) - \Phi(-t_1 - A_0x)|\\
&+ \sup_{t_1 \leq t_1', t_2 \leq t_2', |x| \geq M''} |\Phi(t_1 - Ax) - \Phi(t_1 - A_0x)|\\
&+ \sup_{t_1 \leq t_1', t_2 \leq t_2', |x| \geq M''} |\Psi_1(-t_1 - Ax, -t_2) - \Psi_1(-t_1 - A_0x, -t_2)|\\
&+ \sup_{t_1 \leq t_1', t_2 \leq t_2', |x| \geq M''} |\Psi_1(-t_1 - Ax, t_2) - \Psi_1(-t_1 - A_0x, t_2)|\\
&+ \sup_{t_1 \leq t_1', t_2 \leq t_2', |x| \geq M''} |\Psi_1(t_1 - Ax, -t_2) - \Psi_1(t_1 - A_0x, -t_2)|\\
&+ \sup_{t_1 \leq t_1', t_2 \leq t_2', |x| \geq M''} |\Psi_1(t_1 - Ax, t_2) - \Psi_1(t_1 - A_0x, t_2)|.\end{aligned}$$

By the fact that $A \overset{a.s.}{\to} A_0$ and (53), there exists some $N(M''(\delta, t_1', t_2', A_0, \omega)) > 0$ such that when $n \geq N(M''(\delta, t_1', t_2', A_0, \omega))$,

$$\begin{aligned}&\left\{\Phi(t_1' - AM'') \vee \Psi_1(t_1' - AM'', t_2')\right\} \leq \delta,\\&\left\{\Phi(-t_1' + AM'') \wedge \Psi_1(-t_1' + AM'', -t_2')\right\} \geq 1 - \delta.\end{aligned}$$

Since Φ and Ψ_1 are both non-decreasing continuous functions, for $|x| \geq M''$,

$$\begin{aligned} &\Phi(-t_1 - Ax) \leq \delta, \\ &\Psi_1(-t_1 - Ax, -t_2) \leq \delta, \\ &\Psi_1(t_1 - Ax, -t_2) \leq \delta, \end{aligned} \tag{58}$$

or

$$\begin{aligned} &1 - \delta \leq \Phi(t_1 - Ax), \\ &1 - \delta \leq \Psi_1(-t_1 - Ax, t_2), \\ &1 - \delta \leq \Psi_1(t_1 - Ax, t_2). \end{aligned} \tag{59}$$

Therefore, (56) can be verified by (54)-(55) and (58)-(59) as δ is arbitrary. For (57), as L_0 is Lipschitz with respect to the first argument, it follows that

$$|L_0(Ax, t_1, t_2) - L_0(A_0x, t_1, t_2)| \leq \frac{6}{\sqrt{2\pi}}|Ax - A_0x| < \frac{6M''}{\sqrt{2\pi}}|A - A_0| \tag{60}$$

which completes the proof because $A \overset{a.s.}{\to} A_0$.

$\diamondsuit$

Proof of Lemma 2. We first note that

$$V_m(t_1, t_2) = \sum_{i:\alpha_i = 0} \mathbf{1}\left\{|\breve{Z}_i^U| \geq \hat{r}_i t_1, |\breve{Z}_i^A| \geq \hat{r}_i t_2\right\} = \breve{V}_m(\hat{r}_i t_1, \hat{r}_i t_2)$$

where $\hat{r}_i = \hat{\sigma}_i / \sigma_i$. Define an event

$$\mathcal{A} = \left\{\max_{1 \leq i \leq m} |\hat{\sigma}_i^2/\sigma_i^2 - 1| \to 0\right\} \bigcap \left\{\sup_{t_1 \leq t_1', t_2 \leq t_2'} \left| m_0^{-1} \sum_{i:\alpha_i = 0} \mathbf{1}\left\{|\breve{Z}_i^U| \geq t_1, |\breve{Z}_i^A| \geq t_2\right\} - K_0(t_1, t_2)\right| \to 0\right\}.$$

Then, $\mathbb{P}(\mathcal{A}) = 1$ by Assumption .2 and Lemma 4. For any $\omega \in \mathcal{A}$ and $\delta > 0$, we have $1 - \delta < \hat{r}_i < 1 + \delta$ for any i when m is large enough. For this ω, we have

$$\breve{V}_m((1+\delta)t_1, (1+\delta)t_2) \leq V_m(t_1, t_2) \leq \breve{V}_m((1-\delta)t_1, (1-\delta)t_2)$$

for large enough m. By Assumption .2, we get

$$K_0((1+\delta)t_1, (1+\delta)t_2) \leq \liminf_m \frac{1}{m_0} V_m(t_1, t_2) \leq \limsup_m \frac{1}{m_0} V_m(t_1, t_2) \leq K_0((1-\delta)t_1, (1-\delta)t_2).$$

As K_0 is continuous and δ is arbitrary, we have $V_m(t_1, t_2)/m_0 \to K_0(t_1, t_2)$ for any $\omega \in \mathcal{A}$. Similar arguments can be used to show the rest. $\diamondsuit$

Theorem 1 is proved as follows.

Proof of Theorem 1. By the Glivenko-Cantelli lemma, we can show that the convergence in Lemma 2 holds uniformly, i.e.,

$$\sup_{t_1 \leq t_1^*, t_2 \leq t_2^*} \left| \frac{V_m(t_1, t_2)}{m_0} - K_0(t_1, t_2) \right| \overset{a.s.}{\to} 0 \quad \text{and} \quad \sup_{t_1 \leq t_1^*, t_2 \leq t_2^*} \left| \frac{S_m(t_1, t_2)}{m_1} - K_1(t_1, t_2) \right| \overset{a.s.}{\to} 0.$$

This implies, under Assumption .1,

$$\sup_{t_1 \le t_1^*, t_2 \le t_2^*} |\mathcal{K}_m(t_1,t_2) - K(t_1,t_2)| \xrightarrow{a.s.} 0 \tag{61}$$

where $\mathcal{K}_m(t_1,t_2) = m^{-1}\{V_m(t_1,t_2) + S_m(t_1,t_2)\}$. We next show that

$$\sup_{t_1 \le t_1^*, t_2 \le t_2^*} |\widetilde{\mathrm{FDP}}_\lambda(t_1,t_2) - \mathrm{FDP}_\lambda^\infty(t_1,t_2)| \xrightarrow{a.s.} 0, \tag{62}$$

$$\sup_{t_1 \le t_1^*, t_2 \le t_2^*} \left| \frac{V_m(t_1,t_2)}{V_m(t_1,t_2) + S_m(t_1,t_2)} - \frac{\pi_0 K_0(t_1,t_2)}{K(t_1,t_2)} \right| \xrightarrow{a.s.} 0. \tag{63}$$

To show (62), we first observe that, for large enough m,

$$|\mathcal{K}_m(t_1,t_2) - K(t_1,t_2)| \le \frac{|K(t_1,t_2)|}{2}$$

which implies

$$|\mathcal{K}_m(t_1,t_2)| \ge \frac{|K(t_1,t_2)|}{2} \ge \frac{K(t_1^*,t_2^*)}{2} > 0$$

because $\inf_{t_1 \le t_1^*, t_2 \le t_2^*} |K(t_1,t_2)| \ge K(t_1^*,t_2^*) > 0$. For large enough m, it follows that

$$\begin{aligned}
&|\widetilde{\mathrm{FDP}}_\lambda(t_1,t_2) - \mathrm{FDP}_\lambda^\infty(t_1,t_2)| \\
=& \frac{1}{(1-2\Phi(-\lambda))} \left| \frac{\int L(Ax,t_1,t_2) d\widetilde{G}_{m,n}(x) F_m(\lambda) K(t_1,t_2) - \mathbb{E}_{\boldsymbol{a}^\top \boldsymbol{\xi}}[L_0(A_0 \boldsymbol{a}^\top \boldsymbol{\xi}, t_1, t_2)] F(\lambda) \mathcal{K}_m(t_1,t_2)}{\mathcal{K}_m(t_1,t_2) K(t_1,t_2)} \right| \\
\le& \frac{2}{(1-2\Phi(-\lambda))} \frac{\left| \int L(Ax,t_1,t_2) d\widehat{G}_m(x) F_m(\lambda) K(t_1,t_2) - \mathbb{E}_{\boldsymbol{a}^\top \boldsymbol{\xi}}[L_0(A_0 \boldsymbol{a}^\top \boldsymbol{\xi}, t_1, t_2)] F(\lambda) \mathcal{K}_m(t_1,t_2) \right|}{K(t_1^*,t_2^*)^2}.
\end{aligned}$$

Thus, for (62), it suffices to verify that

$$\sup_{t_1 \le t_1^*, t_2 \le t_2^*} \left| \int L(Ax,t_1,t_2) d\widehat{G}_m(x) F_m(\lambda) K(t_1,t_2) - \mathbb{E}_{\boldsymbol{a}^\top \boldsymbol{\xi}}[L_0(A_0 \boldsymbol{a}^\top \boldsymbol{\xi}, t_1, t_2)] F(\lambda) \mathcal{K}_m(t_1,t_2) \right| \xrightarrow{a.s.} 0,$$

which can be shown by (61) and Lemmas 1-2. We can also show (63) by using similar argument.

As $\mathrm{FDP}_\lambda^\infty(t_1^*,0) < q$ and $\mathrm{FDP}_\lambda^\infty(0,t_2^*) < q$, we have for large enough m,

$$\widetilde{\mathrm{FDP}}_\lambda(t_1^*,0) < q \quad \text{and} \quad \widetilde{\mathrm{FDP}}_\lambda(0,t_2^*) < q,$$

which implies that $\widetilde{T}_1^* \le t_1^*$ and $\widetilde{T}_2^* \le t_2^*$. Thus we have

$$\liminf_m \left\{ \widetilde{\mathrm{FDP}}_\lambda(\widetilde{T}_1^*, \widetilde{T}_2^*) - \frac{V_m(\widetilde{T}_1^*, \widetilde{T}_2^*)}{V_m(\widetilde{T}_1^*, \widetilde{T}_2^*) + S_m(\widetilde{T}_1^*, \widetilde{T}_2^*)} \right\}$$
$$\ge \lim_m \inf_{t_1 \le t_1^*, t_2 \le t_2^*} \left\{ \widetilde{\mathrm{FDP}}_\lambda(t_1, t_2) - \frac{V_m(t_1, t_2)}{V_m(t_1, t_2) + S_m(t_1, t_2)} \right\}$$
$$= \lim_m \inf_{t_1 \le t_1^*, t_2 \le t_2^*} \left\{ \widetilde{\mathrm{FDP}}_\lambda(t_1, t_2) - \mathrm{FDP}_\lambda^\infty(t_1, t_2) + \frac{\pi_0 K_0(t_1, t_2)}{K(t_1, t_2)} - \frac{V_m(t_1, t_2)}{V_m(t_1, t_2) + S_m(t_1, t_2)} \right.$$
$$\left. + \mathrm{FDP}_\lambda^\infty(t_1, t_2) - \frac{\pi_0 K_0(t_1, t_2)}{K(t_1, t_2)} \right\} \ge 0$$

by (62), (63) and the fact that

$$\mathrm{FDP}_\lambda^\infty(t_1, t_2) - \frac{\pi_0 K_0(t_1, t_2)}{K(t_1, t_2)} \ge 0$$

from (12). As $\widetilde{\mathrm{FDP}}_\lambda(\widetilde{T}_1^*, \widetilde{T}_2^*) \le q$, we obtain

$$\limsup_m \frac{V_m(\widetilde{T}_1^*, \widetilde{T}_2^*)}{V_m(\widetilde{T}_1^*, \widetilde{T}_2^*) + S_m(\widetilde{T}_1^*, \widetilde{T}_2^*)} \le q.$$

Finally by Fatou's lemma, we get

$$\limsup_m \widetilde{\mathrm{FDR}}_m \le \mathbb{E}\left[\limsup_m \frac{V_m(\widetilde{T}_1^*, \widetilde{T}_2^*)}{V_m(\widetilde{T}_1^*, \widetilde{T}_2^*) + S_m(\widetilde{T}_1^*, \widetilde{T}_2^*)} \right] \le q.$$

$\diamondsuit$

We justify equation (12) in the following.

Corollary 1. *Under Assumptions .3-.6, for every $t_1, t_2 > 0$, we have*

$$\left| \int L(Ax, t_1, t_2) dG_n(x) - \int L_0(A_0 x, t_1, t_2) dG_0(x) \right| \overset{a.s.}{\to} 0.$$

Proof of Corollary 1. Note that

$$\left| \int L(Ax, t_1, t_2) dG_n(x) - \int L_0(A_0 x, t_1, t_2) dG_0(x) \right|$$
$$= \left| \int L(Ax, t_1, t_2) dG_n(x) - \int L_0(A_0 x, t_1, t_2) dG_n(x) \right|$$
$$+ \left| \int L_0(A_0 x, t_1, t_2) dG_n(x) - \int L_0(A_0 x, t_1, t_2) dG_0(x) \right|.$$

Thus, it suffices to show that

$$|L(Ax, t_1, t_2) - L_0(A_0 x, t_1, t_2)| \overset{a.s.}{\to} 0,$$
$$\left| \int L_0(A_0 x, t_1, t_2)(dG_n(x) - dG_0(x)) \right| \overset{a.s.}{\to} 0,$$

both of which can be verified by using similar arguments as those for Lemma 1. $\diamondsuit$

Note S4: Power analysis

We derive the asymptotic power of the two-dimensional John Storey procedure. Our derivation is heuristic but can be made rigorous under suitable assumptions. Define

$$(T_{1,\text{Two}}, T_{2,\text{Two}}) = \underset{(t_1,t_2)\in\mathcal{F}_{q,\text{Two}}}{\text{argmax}} \; K(t_1, t_2)$$

where $K(t_1, t_2) = \pi_0 K_0(t_1, t_2) + (1 - \pi_0)K_1(t_1, t_2)$ with K_1 and K_2 defined in Assumption .2 and

$$\mathcal{F}_{q,\text{Two}} = \left\{(t_1, t_2) \in \mathbb{R}^+ \times \mathbb{R}^+ : \text{FDP}_\lambda^\infty(t_1, t_2) \le q\right\}.$$

Then, the asymptotic power of the two-dimensional procedure is given by

$$\text{Power}_{\text{Two}} = \lim_{m\to\infty} \frac{\sum_{i:\alpha_i\neq 0} \mathbf{1}\left\{|Z_i^U| \ge T_{1,\text{Two}}, |Z_i^A| \ge T_{2,\text{Two}}\right\}}{m_1} = K_1(T_{1,\text{Two}}, T_{2,\text{Two}}).$$

As a comparison the asymptotic power of the one-dimensional procedure is equal to

$$\text{Power}_{\text{One}} = K_1(0, T_{2,\text{One}})$$

where $T_{2,\text{One}} = \text{argmax}_{t_2\in\mathcal{F}_{q,\text{One}}} K(t_1, t_2)$ with $\mathcal{F}_{q,\text{One}} = \{t_2 \in \mathbb{R}^+ : \text{FDP}_\lambda^\infty(0, t_2) \le q\}$. Since $\mathcal{F}_{q,\text{One}} \subset \mathcal{F}_{q,\text{Two}}$, we have

$$K(T_{1,\text{Two}}, T_{2,\text{Two}}) \ge K(0, T_{2,\text{One}}),$$

that is, the two-dimensional procedure delivers more rejections.

Lemma 11. *Let $M(\lambda) = (1 - 2\Phi(-\lambda))^{-1}(1 - \pi_0)(1 - K_1(0, \lambda))$ and Suppose $FDP_\lambda^\infty(t_1, t_2)$ is a continuous function of (t_1, t_2). Then we have $Power_{Two} \ge Power_{One}$.*

Proof of Lemma 11. Since $\text{FDP}_\lambda^\infty(t_1, t_2)$ is a continuous function of (t_1, t_2), we must have

$$\frac{\pi_0 K_0(T_{1,\text{Two}}, T_{2,\text{Two}}) + M(\lambda)K_0(T_{1,\text{Two}}, T_{2,\text{Two}})}{K(T_{1,\text{Two}}, T_{2,\text{Two}})} = q, \quad \frac{\pi_0 K_0(0, T_{2,\text{One}}) + M(\lambda)K_0(0, T_{2,\text{One}})}{K(0, T_{2,\text{One}})} = q. \tag{64}$$

The fact that $K(T_{1,\text{Two}}, T_{2,\text{Two}}) \ge K(0, T_{2,\text{One}})$ implies both $K_0(T_{1,\text{Two}}, T_{2,\text{Two}}) \ge K_0(0, T_{2,\text{One}})$ from (64) and

$$\begin{aligned}
&(1 - \pi_0)K_1(T_{1,\text{Two}}, T_{2,\text{Two}}) - M(\lambda)K_0(T_{1,\text{Two}}, T_{2,\text{Two}})\\
=&(1 - q)K(T_{1,\text{Two}}, T_{2,\text{Two}})\\
\ge&(1 - q)K(0, T_{2,\text{One}}) = (1 - \pi_0)K_1(0, T_{2,\text{One}}) - M(\lambda)K_0(0, T_{2,\text{One}})
\end{aligned}$$

after rearranging the terms in (64). As $K_0(T_{1,\text{Two}}, T_{2,\text{Two}}) \ge K_0(0, T_{2,\text{One}})$, it follows that

$$\begin{aligned}
(1 - \pi_0)K_1(T_{1,\text{Two}}, T_{2,\text{Two}}) &\ge (1 - \pi_0)K_1(0, T_{2,\text{One}}) + M(\lambda)\left\{K_0(T_{1,\text{Two}}, T_{2,\text{Two}}) - K_0(0, T_{2,\text{One}})\right\}\\
&\ge (1 - \pi_0)K_1(0, T_{2,\text{One}})
\end{aligned}$$

which completes the proof.

◇

References

[1] Benjamini, Y., & Hochberg, Y. (1995). Controlling the false discovery rate: a practical and powerful approach to multiple testing. *Journal of the Royal statistical society: series B (Methodological)*, 57(1), 289-300.

[2] Efron, B. (2012). *Large-scale inference: empirical Bayes methods for estimation, testing, and prediction*. Cambridge University Press.

[3] Jiang, W., & Zhang, C. H. (2009). General maximum likelihood empirical Bayes estimation of normal means. *The Annals of Statistics*, 37(4), 1647-1684.

[4] Kiefer, J., & Wolfowitz, J. (1956). Consistency of the maximum likelihood estimator in the presence of infinitely many incidental parameters. *The Annals of Mathematical Statistics*, 887-906.

[5] Koenker, R., & Mizera, I. (2014). Convex optimization, shape constraints, compound decisions, and empirical Bayes rules. *Journal of the American Statistical Association*, 109(506), 674-685.

[6] Laurent, B., & Massart, P. (2000). Adaptive estimation of a quadratic functional by model selection. *The Annals of Statistics*, 1302-1338.

[7] Leek, J. T., & Storey, J. D. (2008). A general framework for multiple testing dependence. *Proceedings of the National Academy of Sciences*, 105(48), 18718-18723.

[8] Price, A. L., Patterson, N. J., Plenge, R. M., Weinblatt, M. E., Shadick, N. A., & Reich, D. (2006). Principal components analysis corrects for stratification in genome-wide association studies. *Nature genetics*, 38(8), 904.

[9] Rigollet, P., & Hütter, J. C. (2015). *High dimensional statistics*. Lecture notes for course 18S997.

[10] Spearman, C. (1904). The proof and measurement of association between two things. *American journal of Psychology*, 15(1), 72-101.

[11] Storey, J. D. (2002). A direct approach to false discovery rates. *Journal of the Royal Statistical Society: Series B (Statistical Methodology)*, 64(3), 479-498.

[12] Sun, Y., Zhang, N. R., & Owen, A. B. (2012). Multiple hypothesis testing adjusted for latent variables, with an application to the AGEMAP gene expression data. *The Annals of Applied Statistics*, 6(4), 1664-1688.

[13] Vershynin, R. (2010). Introduction to the non-asymptotic analysis of random matrices. arXiv preprint arXiv:1011.3027.

[14] Wang, J., Zhao, Q., Hastie, T., & Owen, A. B. (2017). Confounder adjustment in multiple hypothesis testing. *The Annals of Statistics*, 45(5), 1863-1894.

[15] Zhang, C. H. (2009). Generalized maximum likelihood estimation of normal mixture densities. *Statistica Sinica*, 1297-1318.
