# Supplementary material for: 2dFDR: a new approach to confounder adjustment substantially increases detection power in omics association studies
Source: Genome Biol. 2021 Jul 13;22:208. doi: 10.1186/s13059-021-02418-8 (PMC8276451; doi:10.1186/s13059-021-02418-8)
Supplement: Supplementary file 2 — Additional file 2: Figure S1. Performance comparison when 50% of the features are affected by the confounder. Figure S2. Performance on simulated datasets across varying density (top to bottom) and strength (left to right) of the confounding signals when there are five confounders. Figure S3. Performance across varying density (top to bottom) and strength (left to right) of the confounding signals when the confounding and true signals do not overlap (“NoCoLoc”) and when the true and confounding signals have extensive overlap (“CoLoc”). Figure S4. Performance across varying density (top to bottom) and strength (left to right) of the confounding signals when the errors have a block correlation structure. Figure S5. Performance across varying density (top to bottom) and strength (left to right) of the confounding signals when the errors have the first-order auto-regressive (AR(1)) correlation structure. Figure S6. Performance comparison across varying density (top to bottom) and strength (left to right) of the confounding signals under smaller sample sizes. Figure S7. Performance comparison across varying density (top to bottom) and strength (left to right) of the confounding signals under smaller feature sizes. Figure S8. Performance comparison across varying sample size (top to bottom) and feature size (left to right). Figure S9. The computation time (in seconds) under different sample sizes and feature sizes based on simulated datasets with one confounder, medium density and strength of the true and confounding signals, and a medium confounding level. Figure S10. The decision boundaries of 2dFDR and 1dFDR-A under two unfavorable scenarios for 2dFDR. Table S1. EWAS datasets used in the evaluation of the empirical power of 2dFDR. [file 13059_2021_2418_MOESM2_ESM.pdf]

Additional file 2:
Fig. S1 to S10 and Table S1 for "A new approach to confounder adjustment substantially increases detection power in omics association studies"

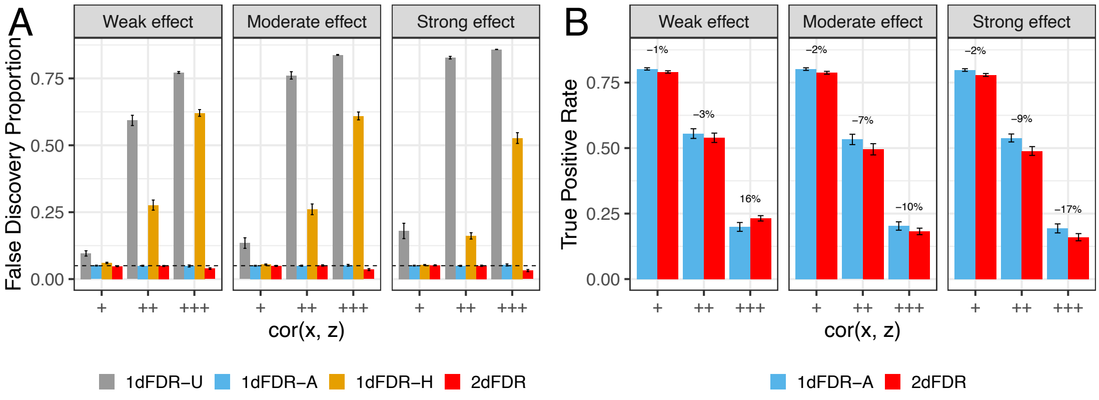

Fig. S1 Performance comparison when 50% of the features are affected by the confounder. From left to right, we increase the strength (effect size) of the confounding signals. False discovery proportions (A) and true positive rates (B) were averaged over 100 simulation runs. Error bars represent the 95% CIs and the dashed horizontal line indicates the target FDR level of 0.05. The density of the true signals is 10% and the strength is moderate. '+', '++' and '+++' represent a low, medium and high correlation be-tween the variable ofinterest and the confounder (ρ = 0 .2, 0.6, 0.8), respectively. 2dFDR maintains FDR at the target level but the power is slightly lower than 1dFDR-A.

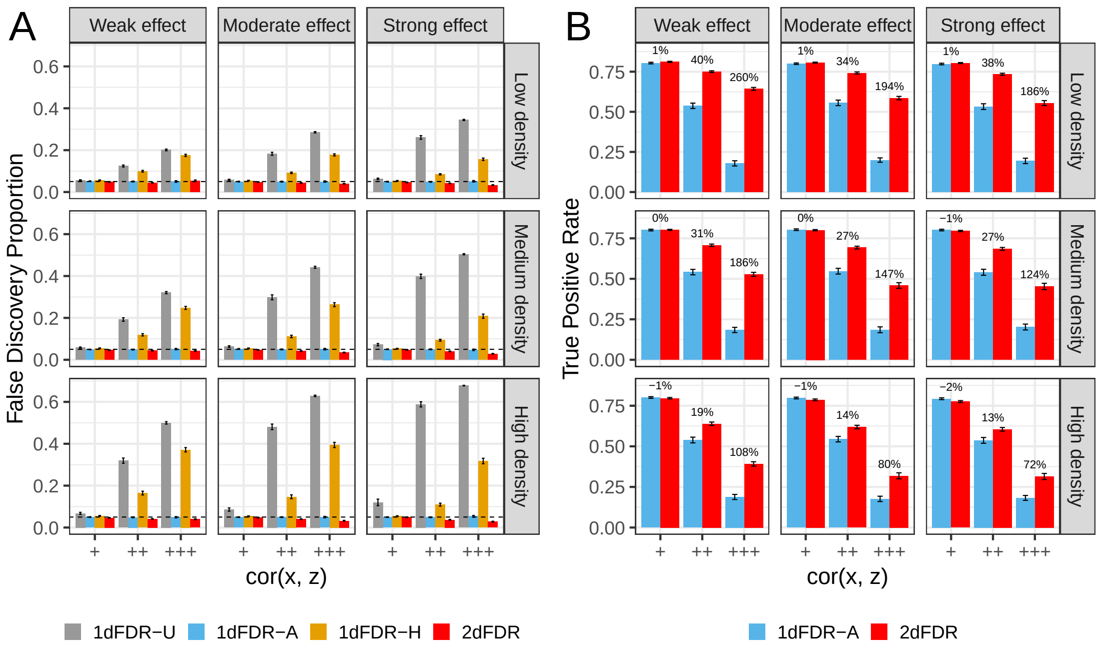

Fig. S2 Performance on simulated datasets across varying density (top to bottom) and strength (left to right) of the confounding signals when there are five confounders. False discovery proportions (A) and true positive rates (B) were averaged over 100 simulation runs. Error bars represent the 95% CIs and the dashed horizontal line indicates the target FDR level of 0.05. The den-sity of the true signals is 10% and the strength is moderate. '+', '++' and '+++' represent a low, medium and high correlation be-tween the variable of interest and the confounders (ρ = 0.2, 0.6, 0.8), respectively. The performance is similar to the setting with one confounder.

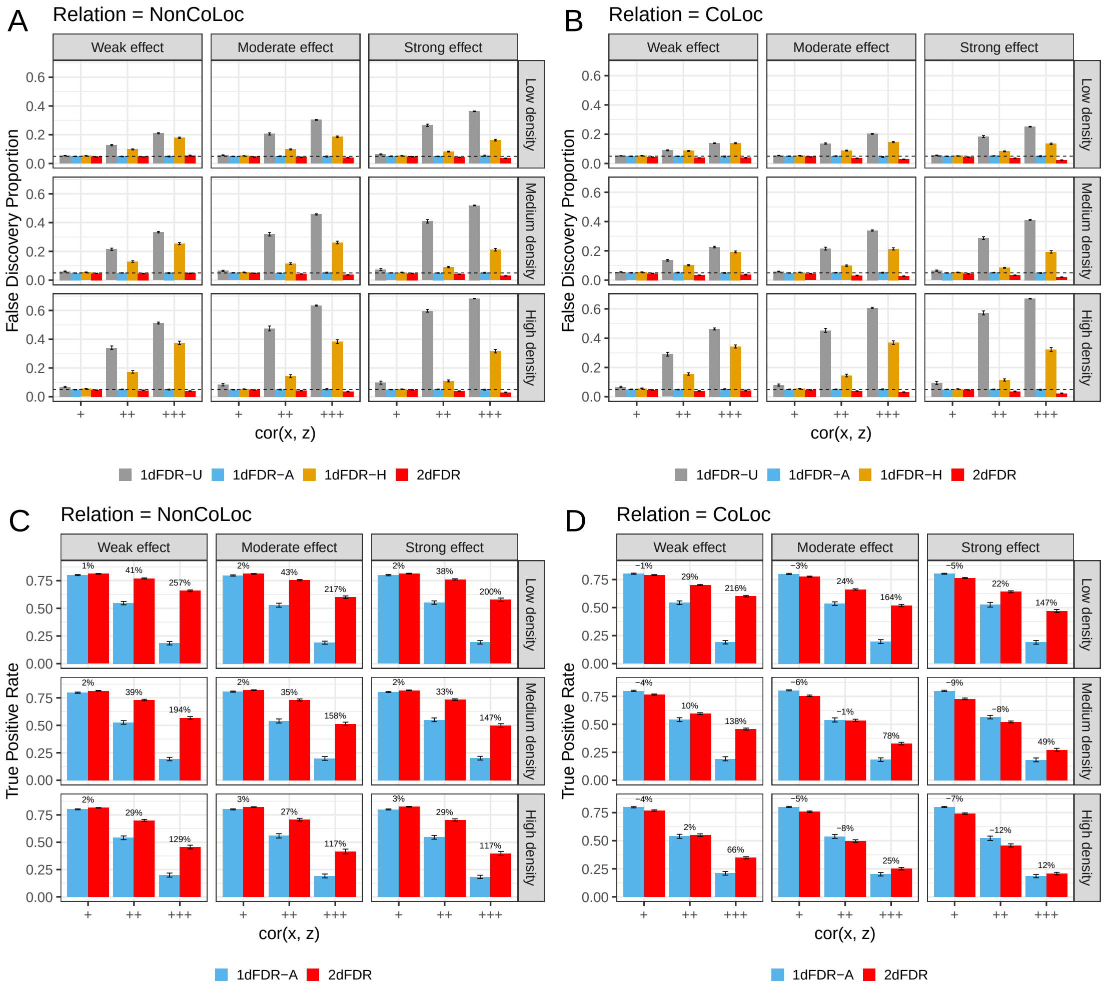

Fig. S3 Performance across varying density (top to bottom) and strength (left to right) of the confounding signals when the confounding and true signals do not overlap ("NoCoLoc") and when the true and confounding signals have extensive overlap ("CoLoc"). False discovery proportions (A, B) and true positive rates (C,D) were averaged over 100 simulation runs. Error bars represent the 95% CIs and the dashed horizontal line indicates the target FDR level of 0.05. The density of the true signals is 10%and the strength is moderate. '+', '++' and '+++' represent a low, medium and high correlation between the variable of interest and the confounder (ρ = 0.2, 0.6, 0.8), respectively. 2dFDR is more powerful than 1dFDR-A when the density of the confounding signals is low and the correlation between the variable of interest and the confounder is high. However, as the confounding signals become denser, the power improvement decreases and 2dFDR is less powerful than 1dFDR-A when the correlation between the variable of interest and the confounder is low.

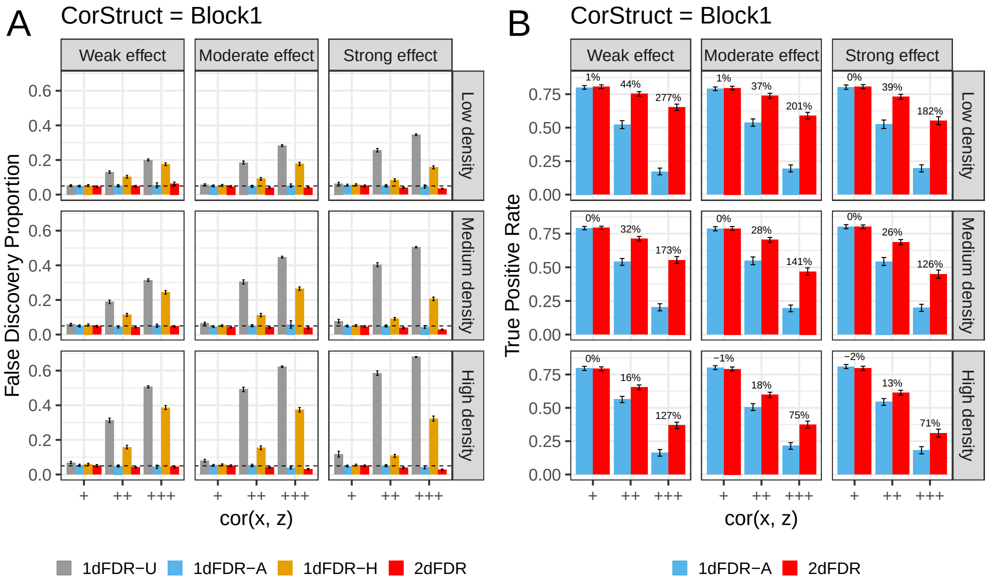

Fig. S4 Performance across varying density (top to bottom) and strength (left to right) of the confounding signals when the errors have a block correlation structure. False discovery proportions (A) and true positive rates (B) were averaged over 100 simulation runs. Error bars represent the 95% CIs and the dashed horizontal line indicates the target FDR level of 0.05. The density of the true signals is 10% and the strength is moderate. '+', '++' and '+++' represent a low, medium and high correlation between the variable of interest and the confounder (ρ = 0.2, 0.6, 0.8), re-spectively. Here we simulate the block correlation structure with positive within-block correlation, which is commonly encountered in genomics data such as gene expression data. Under the block correlation structure, 2dFDR controls the FDR around the target level and the power is similar to that of the independent case.

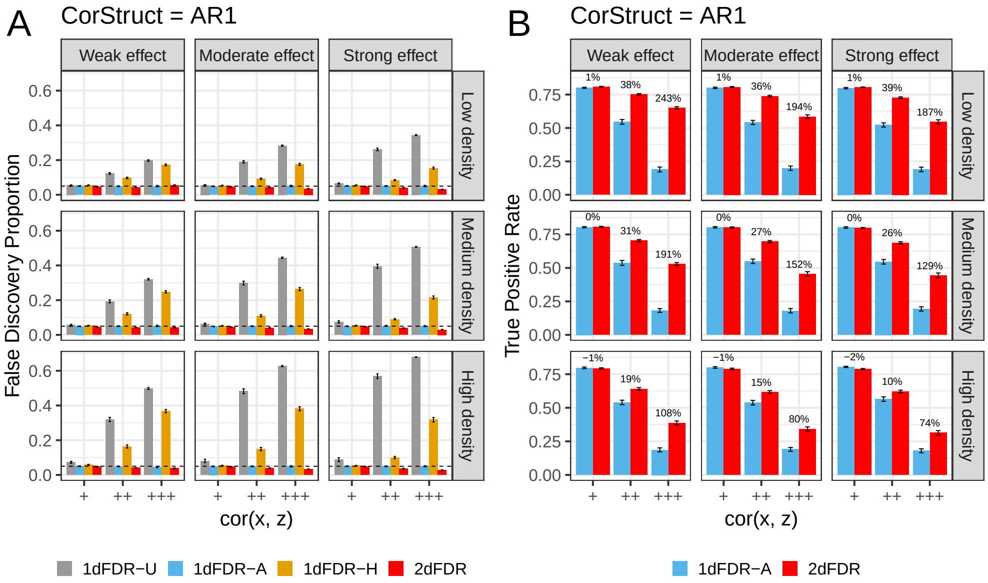

Fig. S5 Performance across varying density (top to bottom) and strength (left to right) of the confounding signals when the errors have the first-order auto-regressive (AR(1)) correlation structure. False discovery proportions (A) and true positive rates (B) were averaged over 100 simulation runs. Error bars represent the 95% CIs and the dashed horizontal line indicates the target FDR level of 0.05. The density of the true signal is 10% and the strength is moderate. '+', '++' and '+++' represent a low, medium and high correlation between the variable of interest and the confounder(ρ = 0.2, 0.6, 0.8), respectively. Here we simulate the AR(1). structure (correlation decays with the distance between the genomic features), which is commonly encountered in genomics data such as DNA methylation data. Under the AR(1) correlation structure, 2dFDR controls the FDR around the target level and the power is similar to that of the independent case

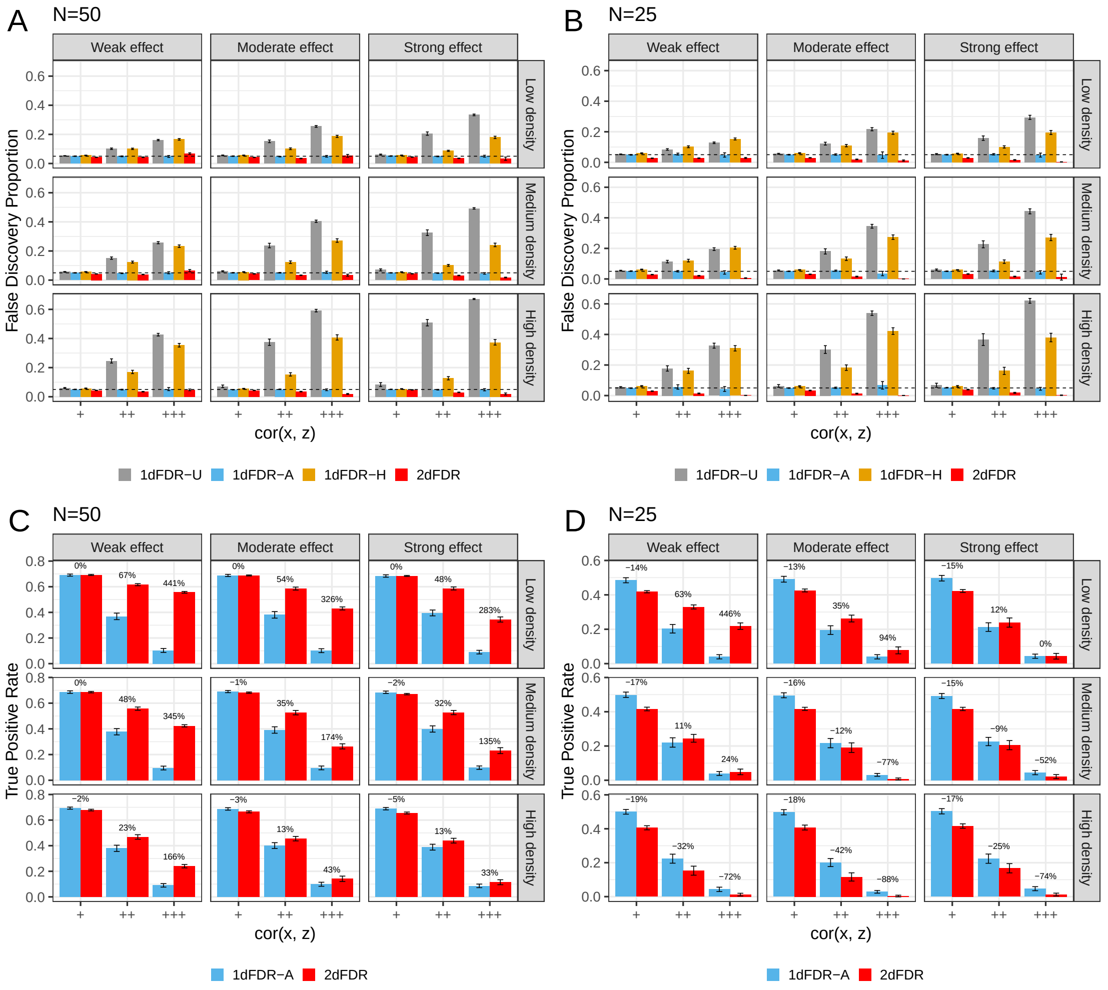

Fig. S6 Performance comparison across varying density (top to bottom) and strength (left to right) of the confound-ing signals under smaller sample sizes. The density of the true signals is 10% and the strength is moderate. '+', '++' and '+++' represent a low, medium and high correlation between the variable of interest and the confounder ($\rho = 0.2$, 0.6, 0.8), respectively. False discovery proportions (A, B) and true positive rates (C,D) were averaged over 100 simulation runs. We observed that the performance at $n = 50$ was similar to that at $n = 100$. At $n = 25$, 2dFDR becomes conser-vative in many settings.

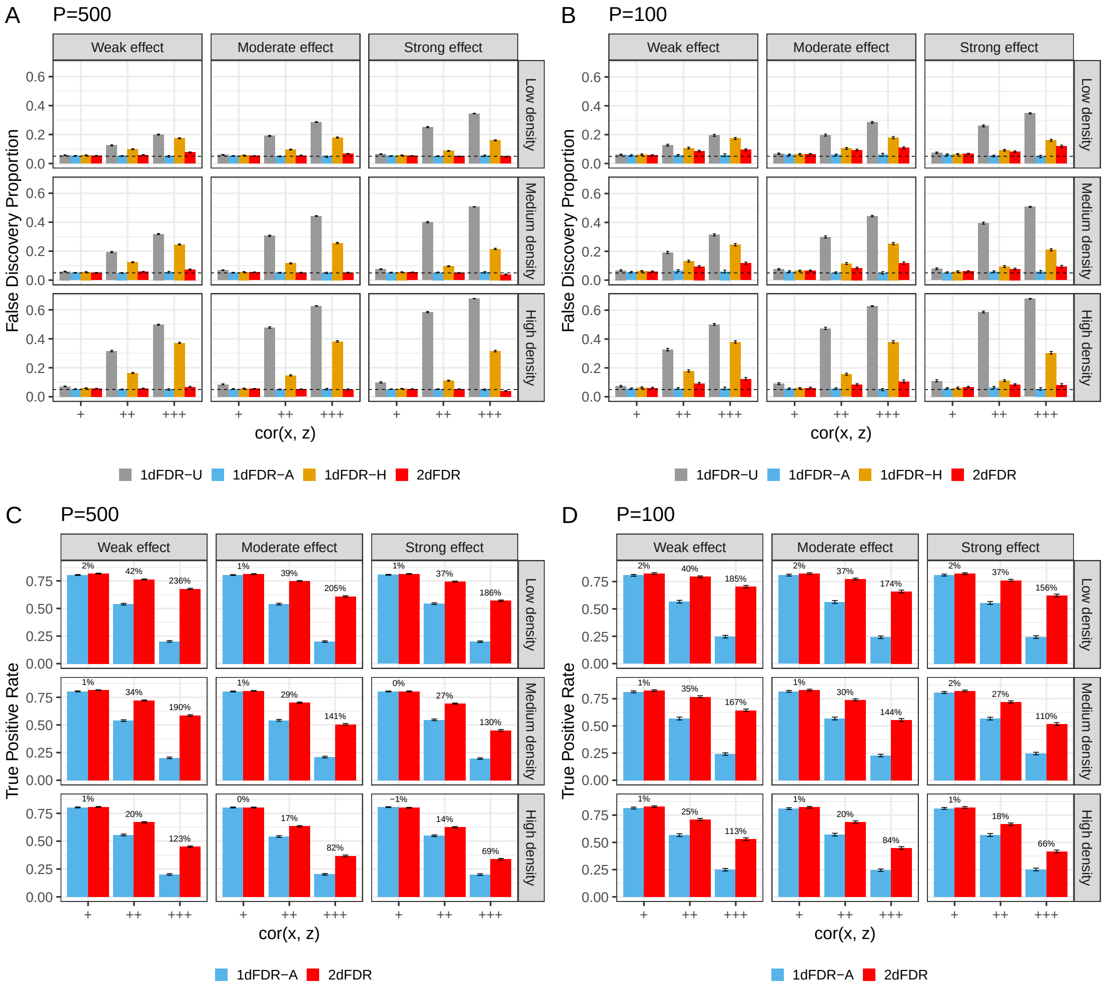

Fig. S7 Performance comparison across varying density (top to bottom) and strength (left to right) of the confounding signals under smaller feature sizes. False discovery proportions (A, B) and true positive rates (C,D) were averaged over 1,000 simulation runs. Error bars represent the 95% CIs and the dashed horizontal line indicates the target FDR level of 0.05. The density of the true signals is 10% and the strength is moderate. '+', '++' and '+++' represent a low, medium and high correlation between the variable of interest and the confounder (ρ = 0.2, 0.6, 0.8), respectively. We observe that the performance at m = 500 is similar to that at m = 10000. However, FDR is inflated at m = 100.

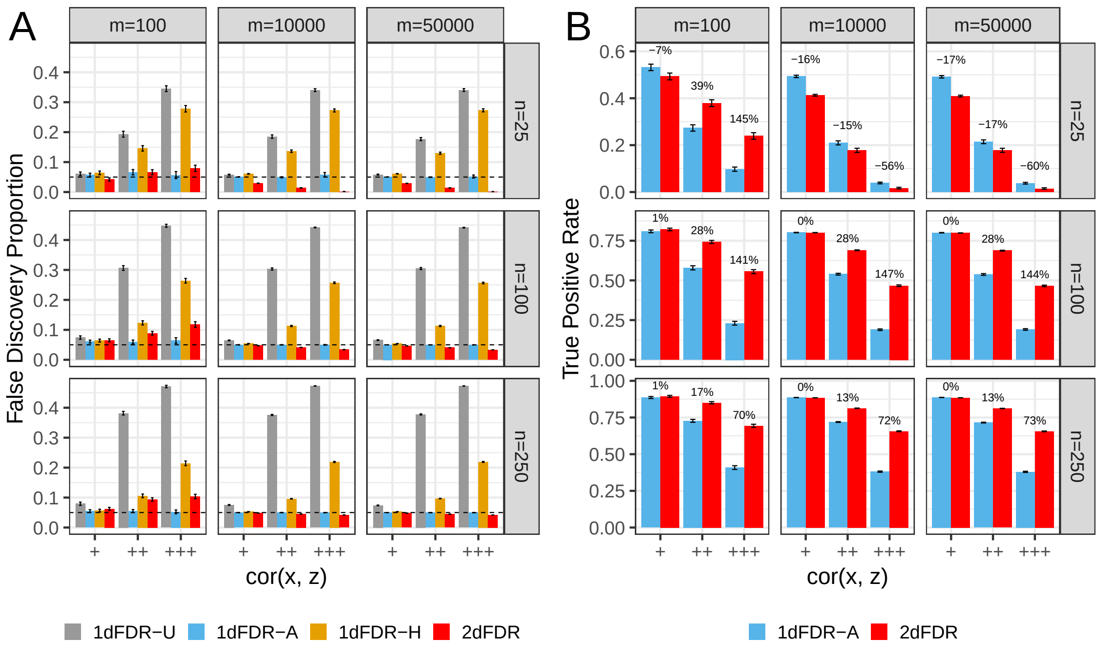

Fig. S8 Performance comparison across varying sample size (top to bottom) and feature size (left to right). False discovery proportions (A) and true positive rates (B) were averaged over 1,000 simulation runs. Error bars represent the 95% CIs and the dashed horizontal line indicates the target FDR level of 0.05. The density of the true/confounding signals is 10% and the strength is moderate. '+', '++' and '+++' represent a low, medium and high correlation between the variable of interest and the confounder(ρ = 0.2, 0.6, 0.8), respectively. When both the sample size and feature size are small, FDR is slightly inflated for 2dFDR. Increasing the sample size or feature size does not rescue the degraded performance due to the other being small.

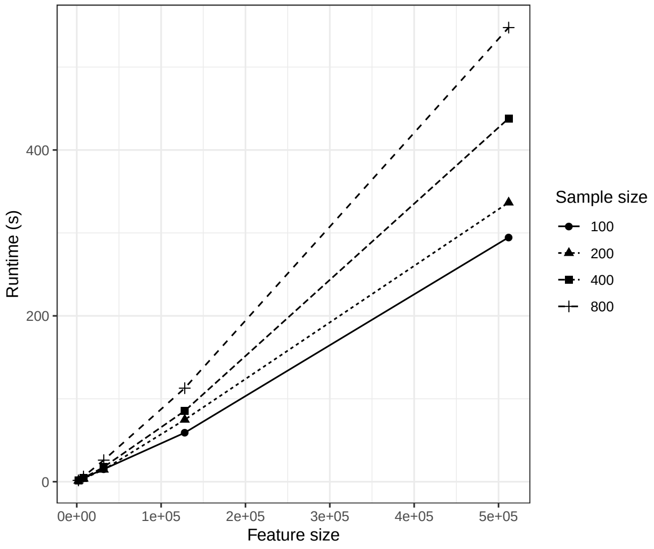

Fig. S9 The computation time (in seconds) under different sample sizes and feature sizes based on simulated datasets with one confounder, medium density and strength of the true and confounding signals, and a medium confounding level. A search grid of 50 × 50 was used without parallelization. The computation was performed under R 3.6.3 and macOS Catalina (v10.15.6) on a MacBook Pro with 2.2 GHz Quad-Core Intel Core i7 and 16 GB 1600 MHz DDR3.

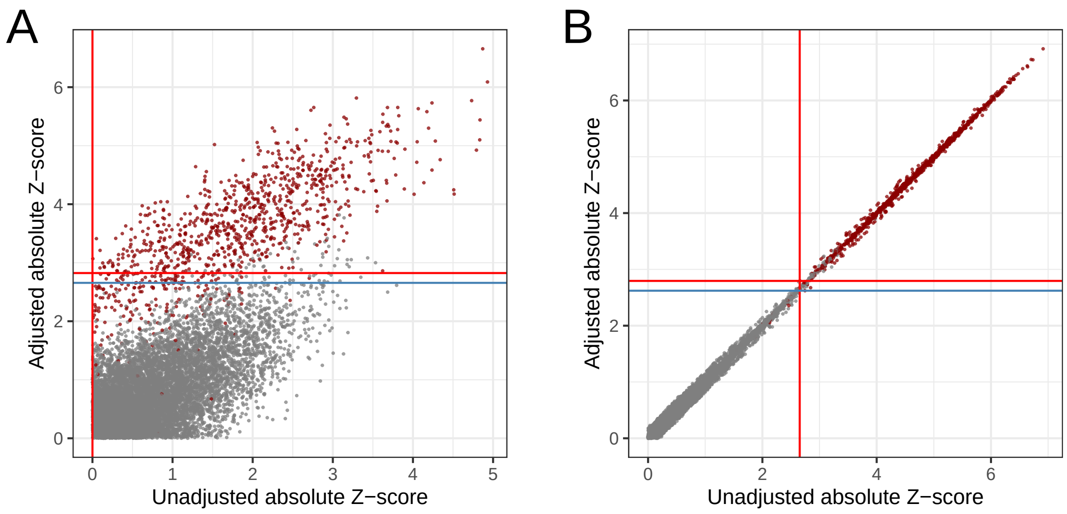

Fig. S10 The decision boundaries of 2dFDR and 1dFDR-A under two unfavorable scenarios for 2dFDR. (A) Colocation of the confounding and true signals with opposite effect sizes so the signals can be revealed only by adjustment. Moderate correlation between the variable of interest and the confounder was simulated ($\rho = 0.6$); (B) Independence between the variable of interest and the confounder. Both data examples were simulated with $n = 100$, $m = 10,000$ and 10% true and confounding signals with moderate strength.

Table S1: EWAS datasets used in the evaluation of the empricial power of 2dFDR

ID	Number of discoveries by 2dFDR	Number of discoveries by 1dFDR-A	% improvement	R^2	GEO accesion	Phenotype	PMID	Tissue	Sample size
EWAS1	301	155	94%	88.9%	GSE101764	Colorectal cancer	28791728	colorectal	261
EWAS10	1	1	0%	14.3%	GSE111223	Parkinson's Disease	26655927	saliva	259
EWAS11	2	1	50%	23.3%	GSE111629	Parkinson's Disease	26655927	blood	571
EWAS12	41	22	83%	31.0%	GSE114753	Smoking	28950428	sperm	156
EWAS13	48	2	1533%	67.9%	GSE39279	Non-small Cell Lung Cancer	24081945	lung	444
EWAS14	75466	80224	-6%	30.4%	GSE40279	Age	23177740	blood	656
EWAS15	5	1	200%	21.9%	GSE40576	Asthma	25769910	blood	194
EWAS16	73	40	80%	49.8%	GSE42861	Rheumatoid Arthritis	23334450	blood	689
EWAS17	3872	4361	-11%	91.8%	GSE47880	Children Born Small For Gestational Age		blood	110
EWAS18	862	227	279%	83.0%	GSE48684	Colorectal Cancer	24793120	colorectal	105
EWAS19	2786	1759	58%	79.2%	GSE49149	Pancreatic Ductal Adenocarcinoma	24500968	pancreatic	196
EWAS2	35	4	620%	23.8%	GSE102468	Panic Disorder	29249830	blood	165
EWAS21	0	0	0%	6.6%	GSE51057	Menarcheal Age	24278132	blood	329
EWAS22	170	75	125%	75.9%	GSE53045	Smoking	24559495	blood	111
EWAS23	30	19	55%	11.4%	GSE53740	Progressive Supranuclear Palsy (PSP) And Frontotemporal Dementia (FTD)	25589773	blood	349
EWAS24	23303	22580	3%	72.6%	GSE54503	Hepatocellular Carcinoma	23208076	liver	132
EWAS25	81	31	156%	89.2%	GSE58218	Anaplastic Gliomas	25008768	brain	157
EWAS26	80	39	103%	93.9%	GSE58888	Age	25888029	blood	143
EWAS27	1150	776	48%	71.0%	GSE59065	Age	26286994	blood	100
EWAS28	822	539	52%	41.5%	GSE59250	Systemic Lupus Erythematosus	23950730	blood	104
EWAS29	87	26	226%	54.4%	GSE59250	Systemic Lupus Erythematosus	23950730	blood	149
EWAS3	4	0	400%	77.4%	GSE103186	Intestinal Metaplasia	29290541	gastric antrum	191
EWAS30	115	6	1557%	89.9%	GSE60132	Age	25806089	blood	192
EWAS31	554	161	243%	83.3%	GSE60185	In Situ And Invasive Carcinoma Of The Breast	25146004	breast	285
EWAS32	1207	1418	-15%	99.5%	GSE61278	Fetal And Adult Liver	25282492	liver	110
EWAS34	19	10	82%	47.7%	GSE64380	Developmental Delay/Intellectual Disability	26003415	blood	100
EWAS35	360	134	167%	19.4%	GSE64495	Syndrome X	25991677	blood	113
EWAS36	20	0	2000%	21.6%	GSE65183	Melanoma	26359985	melanoma	108
EWAS37	4538	4430	2%	26.4%	GSE67393	Sex Differences Of Leukocytes DNA Methylation	26113971	blood	117
EWAS39	31	23	33%	24.6%	GSE69270	Age	26861258	blood	184
EWAS4	4838	4960	-2%	95.5%	GSE104293	Low Grade Glioma	29368212	brain	132
EWAS41	184	90	103%	44.9%	GSE70977	Oral And Pharyngeal Carcinoma	26635906	oral rinse	223
EWAS43	4937	4563	8%	74.7%	GSE72872	Esophageal Adenocarcinoma	26905591	esophagus	104
EWAS44	0	0	0%	7.6%	GSE73103	Obesity	26449484	blood	355
EWAS45	1087	425	155%	29.5%	GSE73115	Age	27498152	blood	180

EWAS47	22	17	28%	48.3%	GSE73894	Psoriatic	26743604	skin	217
EWAS48	17	1	800%	63.1%	GSE74104	Testicular Germ Cell Tumors	27803193	testicular	267
EWAS49	44	15	181%	57.6%	GSE74193	Schizophrenia	26619358	brain	526
EWAS5	8943	6426	39%	85.6%	GSE104707	Barrett's Esophagus And Esophageal Adenocarcinoma	29046735	esophagus	105
EWAS50	35025	35607	-2%	12.6%	GSE74845	Early Ovarian Cancer Evolution	27216078	fallopian tube	216
EWAS51	0	0	0%	37.6%	GSE80261	Human Fetal Alcohol Spectrum Disorder	27358653	buccal epithelial	216
EWAS52	2	0	200%	28.6%	GSE80970	Alzheimer's Disease	29550519	brain	144
EWAS53	0	0	0%	32.0%	GSE80970	Alzheimer's Disease	29550519	brain	142
EWAS54	113	32	245%	81.0%	GSE84207	Breast Cancer	29123100	breast	279
EWAS55	13	2	367%	27.9%	GSE87095	Rheumatoid Arthritis	28475762	blood	122
EWAS56	892	637	40%	76.9%	GSE88883	Breast Cancer	28693600	breast	100
EWAS58	36	0	3600%	81.0%	GSE93933	Non Syndromic Cleft Lip And Palate		blood	126
EWAS59	38	0	3800%	72.7%	GSE97362	CHARGE And Kabuki Syndromes	28475860	blood	125
EWAS6	0	0	0%	12.9%	GSE104942	Susceptibility To Breast Cancer		blood	136
EWAS60	9	2	233%	76.3%	GSE97362	CHARGE And Kabuki Syndromes	28475860	blood	101
EWAS61	16	0	1600%	75.7%	GSE97466	Thyroid Tumors	28938489	thyroid	110
EWAS7	27	9	180%	24.6%	GSE106648	Multiple Sclerosis	29921915	blood	279
EWAS8	58	47	23%	24.0%	GSE107351	Primary Constitutional MLH1 Epimutations		blood	113
EWAS9	16	1	750%	12.2%	GSE109914	Arsenic Exposure	29982128	blood	119

* % improvement is defined as (# TSFDR - # OSFDR) / (# OSFDR + 1), where the addition of 1 is to avoid being divided by 0.

* R^2 (percent explained variance) is calculated by regressing the phenotype on the surrogate variables. It measures the association between the phenotype and the surrogate variables.
