# Supplementary material for: 2dFDR: a new approach to confounder adjustment substantially increases detection power in omics association studies
Source: Genome Biol. 2021 Jul 13;22:208. doi: 10.1186/s13059-021-02418-8 (PMC8276451; doi:10.1186/s13059-021-02418-8)
Supplement: Supplementary file 3 — Additional file 3. Review history. [file 13059_2021_2418_MOESM3_ESM.docx]

Review History

**First round of review**

**Reviewer 1**

**Were you able to assess all statistics in the manuscript, including the appropriateness of statistical tests used? N/A**

**Were you able to directly test the methods? No**

**Comments to author:**

Review of GBIO-D-20-01707, "A new approach to confounder adjustment substantially increases detection power in omics association studies" by Yi et al. The authors present a novel approach for controlling false discovery rate in the presence of confounders. The results are promising, but clarity is needed on when the method may or may not have gains over traditional approaches. Additionally, I was unable to run the method code on my own. Major and minor concerns are detailed below.

Major comments:

1. In the last paragraph of section "Validation of the increased detection power on EWAS datasets", it states "The power improvement was more dramatic at smaller sample sizes." In addition, the discussion states that 2dFDR "performs well under small sample sizes and feature sizes". However, it is earlier recommended not to apply 2dFDR on low sample or feature sizes due to loss of power and/or FDR inflation. Please clarify this contradictory message.

2. A major conclusion of this paper is that the proposed approach improves power in omics association studies. However, many omics analyses involve models more complex than linear models (e.g. generalized linear models for count data that are typical in omics sequencing). A comment is made in the discussion regarding possible extension, but this idea isn't fully explored. Do the major conclusions of the paper hold under this extension?

3. As far as I can tell, the code submitted in the CodeOcean capsule does not actually run the method. It is preloaded with all the results files, and the code provided is simply to arrange the results in figures. While this is an important step for reproducibility, it doesn't demonstrate the use of the software package developed by the authors, nor provide the information necessary for readers to use the code on their own data. However, the relevant code for generating results appears to be available on the tdfdr github page, along with an R package. Unfortunately, when I followed the installation instructions for the package and its dependencies and then tried to run the example code in the readme file, the tdfdr function call resulted in an error: "Error in if (status != "OPTIMAL") warning(paste("Solution status = ", : argument is of length zero" which is not informative, so I'm unable to run the method.

4. Additional justification/citation needed for the claim "However, confounders usually affect only a subset of omics features, and adjusting confounders for every omics feature will be an over-adjustment, leading to substantial power loss" on line 63-65

5. What would happen if the signal is only seen after adjusting for the confounder? Will it be weeded out by Zu?

6. What are the sample sizes and number of features for the TCGA and metabolite analyses described in the section "Evaluation of the detection power on real omics datasets"? Are they subject to the effects of small sample sizes and/or small feature sizes, which can lead to inflated FDR? If so, then the metrics in Figure 4 are not necessarily meaningful.

7. For the real data examples, what is the overlap like between significant features found by 1d and 2dFDR? Are they finding completely different sets of features? Or a common set, with additional found by the method that makes more discoveries?

Minor comments:

1. Interesting to see the effects in GWAS - is there a natural extension to 2dFWER?

2. The paper does not comment on computational feasibility (e.g. speed, memory usage, parallelization capabilities) of the approach.

3. What is the difference between +, ++, +++ and columns in figure 3? Both are confounder strength?

4. Lines in Figure 4A-C don't have a legend, so it's not clear which line represents each method. In addition, would be helpful to label these so it's easier to distinguish between panels without relying on going back and forth between legend and figure.

**Reviewer 2**

**Were you able to assess all statistics in the manuscript, including the appropriateness of statistical tests used? None**

**Were you able to directly test the methods? Yes**

**Comments to author:**

In this manuscript, the authors propose a method to make confounder adjustment for association studies in large omics data. The method uses a 2-dimensional FDR-based approach with two appropriate statistics used for the rejection criteria. It has been shown that the method achieves asymptotic FDR control and increases detection power using theoretical results. Both simulated and real data were analyzed to demonstrate that the proposed method achieves substantial increase in power under certain situations.

Overall, the manuscript has its merits, but there are some concerns/suggestions. The concerns and the suggestions are given below in order of their importance.

1.	One limitation of the motivation for this approach is that it does not apply when a confounder hides a true association. Such situations may arise in real data sets, especially when there are more than one confounders. This is briefly discussed in the discussion section where the authors claim that “such special cases to be relatively uncommon for a data set”. However, there is no justification provided why that is expected to rare. There should be a better justification and/or more discussion on this. It should be noted that the simulations did not rule out such possibilities and the proposed method still performed quite well.

2.	Simulations parameters (signal strength, confounding strength, etc) are somewhat arbitrary. Are they motivated by real data? Please discuss more. Since real data are also analyzed, it should be easy to find estimated parameters from the data and use similar values in the simulations.

3.	Intuitively, are the two methods 2dFDR and 1dFDR equivalent in some sense when there is no confounding? Some insight might be good. This is especially useful since we see very similar performance of the two methods for “weak” confounding, and in fact 1dFDR is slightly better in some of the cases based on simulation results.

4.	Did the authors consider performing any simulations where there are more than one confounders?

5.	Fig 1: 1A seems somewhat redundant given 1B. It also makes the reader wonder what amount of confounding was used in 1A which is not mentioned in the figure captions.

Also, please use appropriate superscripts in the axis labels. lZal and lZul are confusing.

6.	Line 133: The description of figure 3 is confusing. The authors should make clear distinction between “confounding strength” and “confounding signal strength” and define them clearly. The figure caption of figure 3 is somewhat confusing too. These become clearer once the reader goes through the details of the simulations, but it should be made clear up front. Similarly, define “colocation” when it is discussed for the first time in line 141.

7.	Line 139-140: “Higher strength of the confounding signals (left to right) reduced the power difference slightly.” – Do the authors have any insight why this might be the case? In the simulations, the confounding signals are assumed to be in the same scale with the signals of the variables of interest. If the confounding signals are much larger, can that be an issue?

8.	Lines 154-163: The discussion on smaller sample sizes or smaller feature sizes is useful, but these simulations are performed by making one of them small while holding the other one at a large value. It will also be interesting to study the joint behavior; e.g. the following questions come in mind: (i) what happens when both are small? (ii) can the problem of small feature size be overcome to some extent by having a larger sample size and vice versa?

9.	It will also be interesting to see a plot like Fig1A for the cases where 2dFDR performs worse compared to 1dFDR to better understand where it fails. (May be a supplementary figure?)

10.	I suggest that the feature sizes of each data set is mentioned under “Evaluation of the detection power on real omics datasets”.

11.	Fig 4A: The legend does not show which curve is which.

12.	Line 178: Do the authors have any insight on why the power increase is more dramatic if the analysis is confined to the polar metabolites?

13.	Line 196: “(adjusted) p-value” should be defined somewhere in the main manuscript. Line 361 can be more clear on this.

14.	Lines 215-217: “The power improvement was more dramatic at smaller sample sizes”. It sounds counterintuitive since we saw earlier that 2dFDR did not perform very well when the sample size was very small. In fact, the power improvement here is most dramatic for moderate sample sizes such as 60, 80. Please rephrase these sentences.

15.	Lines 230-231: “performs well under small sample sizes and feature sizes” – More careful phrasing should be used since it does not perform as well when sample size and/or feature size becomes very small.

16.	Lines 237-239: One wonders how much the performance may suffer if indeed we apply 2dFDR for a data with very high density of confounding. A short simulation study might be useful.

17.	Line 364: Averaging over 100 simulations may be too small. We recommend 1000 simulations, at least for showing the FDR control. Although the confidence intervals seemed to be narrow enough in most situations, it is not the case for some figures, e.g. Fig S2.

18.	Fig 2B compared to other figures: For consistency, please include the percentage points for other figures too. This looks especially strange as the first bar charts in the same figure do not have the percentage points.

19.	Fig S1: “or 50% (“CoLoc”) of the true signals colocate with the confounding signals”. This is not fully accurate for all cases since the simulation procedure is based on the 50% of the smaller proportion (lines 343-344).

20.	Fig S1: “Although in theory the power of 2dFDR dominates 1dFDR-A, the numerical precision of finding the optimal cutoff values on the two dimensions could affect the power slightly.” – I suspect that there could be more to this beyond the numerical precision. The power difference is more than ‘slight’ (up to approximately 8 percentage points in some cases). Can it be the case that the asymptotics do not apply well under certain situations?

21.	Line 152: “FDR was controlled near the target level” – It seems like the FDR was inflated by as much as 60% compared to the target level in some cases. However, it can be argued that the FDR is inflated for the 1dFDR methods for these cases too, and the 2dFDR method doesn’t do any worse compared to 1dFDR.

22.	Some notes on the computation time should be included somewhere in the manuscript.

**Authors Response**

**Point-by-point responses to the reviewers’ comments:**

Reviewer #1

The authors present a novel approach for controlling false discovery rate in the presence of confounders. The results are promising, but clarity is needed on when the method may or may not have gains over traditional approaches. Additionally, I was unable to run the method code on my own. Major and minor concerns are detailed below.

Major comments:

1. In the last paragraph of section "Validation of the increased detection power on EWAS datasets", it states "The power improvement was more dramatic at smaller sample sizes." In addition, the discussion states that 2dFDR "performs well under small sample sizes and feature sizes". However, it is earlier recommended not to apply 2dFDR on low sample or feature sizes due to loss of power and/or FDR inflation. Please clarify this contradictory message.

*Response: Thanks for pointing out such seemingly contradictory descriptions. The contradiction comes from the definition of a “small” sample size. Based on the study of the effect of sample size (Supplementary Fig. S6), 2dFDR performs similarly well at n=50 compared to n=100 in the main simulations. However, when n=25, 2dFDR becomes less powerful than the traditional 1dFDR-A unless the confounder signals are very sparse/strong. In Fig 5C, we observe that 2dFDR performs well at n = 40. We thus performed additional simulations with a sample size 40. We found that 2dFDR still performs much better than 1dFDR-A (see the picture below). Moreover, when the absolute improvement instead of the percentage improvement is used, the improvement is greater at moderate sample sizes. We thus removed the statement "The power improvement was more dramatic at smaller sample sizes” in the results. We also rephrased the statement "(2dFDR) performs well under small sample sizes and feature sizes" in the discussion to "(2dFDR) performs well at moderate sample and feature sizes”.*

2. A major conclusion of this paper is that the proposed approach improves power in omics association studies. However, many omics analyses involve models more complex than linear models (e.g. generalized linear models for count data that are typical in omics sequencing). A comment is made in the discussion regarding possible extension, but this idea isn't fully explored. Do the major conclusions of the paper hold under this extension?

*Response:* *Thanks for this insightful comment. In fact, we are currently working on such an extension to the generalized linear models (GLM). Under the GLM framework, the response is modeled by the exponential family with the probability density such that*

*where* $g$ *is a known link function. Based on the asymptotic normality of the GLM estimators, we can again define the z-statistic for testing whether* $\alpha_{i}=0$ *after adjusting for the confounding factors, and the unadjusted version without adjusting for the confounding factors, i.e., setting* $\beta_{i}=0$*. Different from the case of linear models, the adjusted/unadjusted estimators for* $\alpha_{i}$*do not have closed forms. However, we still managed to derive the asymptotic joint distribution of the two z-statistics. Using this result, it turns out that the nonparametric empirical Bayes procedure is still applicable with some minor modifications. We believe that the paper's major conclusions hold under this extension, which is partly confirmed by our preliminary numerical results. A manuscript is under preparation, and we plan to report the detailed results elsewhere.*

3. As far as I can tell, the code submitted in the CodeOcean capsule does not actually run the method. It is preloaded with all the results files, and the code provided is simply to arrange the results in figures. While this is an important step for reproducibility, it doesn't demonstrate the use of the software package developed by the authors, nor provide the information necessary for readers to use the code on their own data. However, the relevant code for generating results appears to be available on the tdfdr github page, along with an R package. Unfortunately, when I followed the installation instructions for the package and its dependencies and then tried to run the example code in the readme file, the tdfdr function call resulted in an error: "Error in if (status != "OPTIMAL") warning(paste("Solution status = ",  : argument is of length zero" which is not informative, so I'm unable to run the method.

*Response: We are sorry to know that the reviewer was unable to install and test our tdfdr package. This may be due to the dependency on the mosek software, which requires setting proper search paths. We now removed the dependency on mosek and used the R implementation of NPEB estimation (nspmix package). We have verified that the new implementation produces virtually identical results. Now we believe that tdfdr can be installed more easily. On the GitHub page, we have put all the codes used in this study and an example was also given to show how to run our package. The reviewer could also see the detailed usage by using “?tdfdr” in the R environment after installing the package.*

4. Additional justification/citation needed for the claim "However, confounders usually affect only a subset of omics features, and adjusting confounders for every omics feature will be an over-adjustment, leading to substantial power loss" on line 63-65

*Response: Thanks. We now added four references showing that the common confounders such as sex and age affect a subset of features:*

- Lu T, Pan Y, Kao S-Y, Li C, Kohane I, Chan J, Yankner BA: Gene regulation and DNA damage in the ageing human brain. Nature. 2004, 429: 883-891. 10.1038/nature02661. *A study of postmortem human brain tissue from 30 individuals aged 26 to 106 years showed that approximately 4% of approximately 11,000 genes analyzed show a significant age-related expression change in individuals aged >40 years.*
- Glass, D., Viñuela, A., Davies, M. N., Ramasamy, A., Parts, L., Knowles, D., ... & Spector, T. D. (2013). Gene expression changes with age in skin, adipose tissue, blood and brain. Genome biology, 14(7), 1-12. *Using a linear mixed model, differential expression with age was identified in 1,672 genes in skin and 188 genes in adipose tissue.*
- Gershoni, M., & Pietrokovski, S. (2017). The landscape of sex-differential transcriptome and its consequent selection in human adults. BMC biology, 15(1), 1-15. *Sex-differential expression (SDE) was tested in each of the 45 common tissues by comparing the individual expression values of 18,670 out of 19,644 informative protein-coding genes in men versus women. They identified 1559 sex-specific and moderately sex-specific genes.*
- Chen, J., Behnam, E., Huang, J., Moffatt, M. F., Schaid, D. J., Liang, L., & Lin, X. (2017). Fast and robust adjustment of cell mixtures in epigenome-wide association studies with SmartSVA. BMC genomics, 18(1), 1-13. *Table SN1.1 shows that the percentage of differential CpG sites between any two leukocyte subtypes ranges from 16% to 34% based on a two-component mixture model.*

5. What would happen if the signal is only seen after adjusting for the confounder? Will it be weeded out by Zu?

*Response: Thanks for this insightful question, which the other reviewer also raised. Yes, if the signal is only seen after adjusting for the confounder, we may lose some of these signals during the screening phase. The optimal cutoff of* ${|Z}_{i}^{U}|$ *is determined based on the tradeoff between power reduction due to erroneously excluding these relevant features in the first dimension and power increase due to reducing the multiple testing burden and increasing the signal density in the second dimension. If all the signals are only seen after adjusting for the confounder, the best cutoff on* ${|Z}_{i}^{U}|$ *should be 0 and 2dFDR is then reduced to the traditional 1dFDR-A. However, in finite samples, it may not always be possible to reduce 2dFDR to 1dFDR-A exactly and 2dFDR could be slightly less powerful than 1dFDR-A in such situations (****Supplementary Fig. S10A****). We added more discussion on this point.*

6. What are the sample sizes and number of features for the TCGA and metabolite analyses described in the section "Evaluation of the detection power on real omics datasets"? Are they subject to the effects of small sample sizes and/or small feature sizes, which can lead to inflated FDR? If so, then the metrics in Figure 4 are not necessarily meaningful.

*Response: The sample size and feature size for the TCGA are 342 and 19,329, respectively and those for metabolite analyses are 289 and 1,201, respectively. Based on the evaluation, our method is robust at m = 500 and n= 50 (Supplementary Fig. S6&7). Therefore, the increased power is less likely due to FDR inflation. However, for the polar metabolites, the feature size (m=325) is on the small side. To be conservative, we now removed this in the main text since it did not provide additional insights.*

7. For the real data examples, what is the overlap like between significant features found by 1d and 2dFDR? Are they finding completely different sets of features? Or a common set, with additional found by the method that makes more discoveries?

*Response: Thanks for this great comment! We now did more investigation on the metabolomics dataset (no hits for 1dFDR-A on the transcriptomics dataset at 5%). Venn diagram below shows the overlap between the two methods at 5% FDR. We can see 2dFDR could find the majority of the metabolites by 1dFDR-A. It also recovered a much larger set of features missed by 1dFDR-A. We added some descriptions of the overlap pattern in the results.*

Minor comments:

1. Interesting to see the effects in GWAS - is there a natural extension to 2dFWER?

*Response: Yes, the proposed methodology can be naturally extended to 2dFWER. Let us*

*briefly describe the idea. Recall that the 2d rejection region is given by* $\{1\leq i\leq m:\left| Z_{i}^{U} \right|\geq t_{1}, \left| Z_{i}^{U} \right|\geq t_{2}$*}. Denote* $\mathcal{M}_{0}$ *the set of all true null hypotheses. Then we have*

*Where* $P_{0}$ *denotes the probability under the null hypothesis. It thus seems natural to define*

*A rigorous theoretical justification of this procedure requires nontrivial modification of*

*the current proofs, and we prefer to leave it for future research.*

2. The paper does not comment on computational feasibility (e.g. speed, memory usage, parallelization capabilities) of the approach.

*Response: The package has parallelization capabilities and can be run in parallel on each grid point. We now included a supplementary figure (Supplementary Fig. S9) showing the computational time for running an instance at various sample sizes (100, 200, 400, 800) and feature sizes (2k, 8k, 32k, 128k, 512k). At n=800, m=512k and one confounder, it completes the analysis in 546s (9.1 mins) using a search grid of* $50\times50$ *without parallelization on a MacBook Pro laptop. The memory requirement is the same as fitting regular linear regressions and requires only accommodating a matrix multiplication of* $A_{p\times n} B_{n\times m}$*, where* $p, n, m$ *are the number of covariates, sample size and feature size, respectively. We added a paragraph on computational efficiency in the simulation result section.*

3. What is the difference between +, ++,  +++ and columns in figure 3? Both are confounder strength?

*Response: ‘+’, ‘++’,  ‘+++’ represent the magnitude of correlation between the variable of interest and the confounder (confounding level) while columns represent the effect sizes (strength) of the confounding signals. We now clearly defined the terms “confounding level”, “confounding signal”, “true signal”, “signal density” and “signal strength” up front, following the suggestion of the other reviewer. We also changed the “confounding” to “confounding level” in the figures.*

4. Lines in Figure 4A-C don't have a legend, so it's not clear which line represents each method. In addition, would be helpful to label these so it's easier to distinguish between panels without relying on going back and forth between legend and figure.

*Response: Thanks for the suggestion. We now provided labels for each panel.*

Reviewer #2

In this manuscript, the authors propose a method to make confounder adjustment for association studies in large omics data. The method uses a 2-dimensional FDR-based approach with two appropriate statistics used for the rejection criteria. It has been shown that the method achieves asymptotic FDR control and increases detection power using theoretical results. Both simulated and real data were analyzed to demonstrate that the proposed method achieves substantial increase in power under certain situations.

Overall, the manuscript has its merits, but there are some concerns/suggestions. The concerns and the suggestions are given below in order of their importance.

1. One limitation of the motivation for this approach is that it does not apply when a confounder hides a true association. Such situations may arise in real data sets, especially when there are more than one confounders. This is briefly discussed in the discussion section where the authors claim that “such special cases to be relatively uncommon for a data set”. However, there is no justification provided why that is expected to rare. There should be a better justification and/or more discussion on this. It should be noted that the simulations did not rule out such possibilities and the proposed method still performed quite well.

*Response: Thanks for this insightful comment, which the first reviewer also made. We now revised the statement and expanded the discussion. See below:*

*“Since 2dFDR depends on the unadjusted statistic to filter features, when the confounder and variable of interest have opposite effects on the omics feature with similar magnitude, they will cancel out each other's effect, and the omics feature could be excluded erroneously in the first dimension. The optimal cutoff of* ${|Z}_{i}^{U}|$ *is thus determined based on the tradeoff between power reduction due to erroneously excluding these relevant features in the first dimension and power increase due to reducing the multiple testing burden and increasing the signal density in the second dimension. If all the signals are only seen after adjusting for the confounder, the best cutoff on* ${|Z}_{i}^{U}|$ *should be 0 and 2dFDR is then reduced to the traditional 1dFDR-A. However, in finite samples, it may not always be possible to reduce 2dFDR to 1dFDR-A exactly and 2dFDR could be less powerful than 1dFDR-A in such situations (Supplementary Fig. S10A). “*

2. Simulations parameters (signal strength, confounding strength, etc) are somewhat arbitrary. Are they motivated by real data? Please discuss more. Since real data are also analyzed, it should be easy to find estimated parameters from the data and use similar values in the simulations.

*Response: Thanks for this excellent suggestion. Instead of using a specific parameter value based on a few real datasets, we use a parameter sweep to create different degrees of signal density, signal strength, confounder density, confounder strength and the correlation between the confounder and the variable of interest, so we could have a less biased evaluation of the proposed method. The ranges of these parameters are designed to cover many real-world settings. For example, the confounder density 5%, 10% and 20% are close to what was reported in the literate for different types of confounders such as sex, age and cell type composition. Sex and age were reported to affect the transcription of 5-10% genes while the percentage of cell type-specific methylation could range between 14%-34% (we added these references as suggested by the other reviewer. Please also see our response to comment 4 of the other reviewer). For signal strength, we vary it so the power ranges from low to high and the confounder strength is then taken to be smaller than, similar to, and larger than the signal strength. As for the correlation between the confounder and the variable of interest, we could see a wide range in real data (see the R^2^ in Supplementary Table 1), so we studied a correlation of 0.2, 0.6, and 0.8, representing weak, moderate and strong confounding.*

3. Intuitively, are the two methods 2dFDR and 1dFDR equivalent in some sense when there is no confounding? Some insight might be good. This is especially useful since we see very similar performance of the two methods for “weak” confounding, and in fact 1dFDR is slightly better in some of the cases based on simulation results.

*Response: Yes, the reviewer’s intuition is correct. When there is no confounding, adjusting the covariates will lead to power improvement if the covariate has effects on the outcome and will not hurt the power much if the covariate has no effects on the outcome (however, when the sample size is very small or the number of covariates is very large, adjusting irrelevant covariates could lead to power loss). Therefore, when there is no confounding, performing adjusted analyses may be a good choice. In the absence of confounding, the test statistic* $Z_{i}^{U}$ *and* $Z_{i}^{A}$ *are almost perfectly correlated (Supplementary Fig. 10B), and performing two-dimensional FDR control based on* $Z_{i}^{U}$ *and* $Z_{i}^{A}$ *is almost equivalent to performing one-dimensional FDR control based on only* $Z_{i}^{A}$ *. Asymptotically, if there is no confounding, 2dFDR could be reduced to 1dFDR. However, in finite sample, there could be some power loss when there is no confounding or the confounding is very weak. Fortunately, the confounding level (correlation between the confounder and the variable of interest) is known before the analysis. We thus recommend running 2dFDR only when there is significant confounding. We now added more discussion on this point.*

4. Did the authors consider performing any simulations where there are more than one confounders?

*Response: We now provided additional simulations with five confounders. Please see the new Supplementary Fig. S2. We can see the performance is very similar to that based on one confounder.*

5. Fig 1: 1A seems somewhat redundant given 1B. It also makes the reader wonder what amount of confounding was used in 1A which is not mentioned in the figure captions.
Also, please use appropriate superscripts in the axis labels. lZal and lZul are confusing.

*Response: Thanks. To facilitate explanation, we decide to keep Figure 1A even it is redundant. Figure 1A is based on a highly confounded situation (confounder +++,* $\rho=0.8$*). We now explicitly stated the amount of confounding in the legend. We also corrected the labels.*

6. Line 133: The description of figure 3 is confusing. The authors should make clear distinction between “confounding strength” and “confounding signal strength” and define them clearly. The figure caption of figure 3 is somewhat confusing too. These become clearer once the reader goes through the details of the simulations, but it should be made clear up front. Similarly, define “colocation” when it is discussed for the first time in line 141.
*Response: Thanks for this nice suggestion to help improve the clarity of our writing. We now clearly defined these terms upfront. To avoid confusion, we did not use “confounding strength” and “confounding signal strength” at the same time. Instead, we use “confounding level” instead of “confounding strength” to represent the correlation between the confounder and the variable of interest.*

7. Line 139-140: “Higher strength of the confounding signals (left to right) reduced the power difference slightly.” – Do the authors have any insight why this might be the case? In the simulations, the confounding signals are assumed to be in the same scale with the signals of the variables of interest. If the confounding signals are much larger, can that be an issue?

*Response: If the confounding signals’ strength is much larger, adjustment could help reveal the true signal when the feature is affected by both the confounder and the variable of interest. Thus, adjustment will improve the power for those features affected by both while power remains the same for those features affected by only the variable of interest. This explains the decrease in the power difference.*

8. Lines 154-163: The discussion on smaller sample sizes or smaller feature sizes is useful, but these simulations are performed by making one of them small while holding the other one at a large value. It will also be interesting to study the joint behavior; e.g. the following questions come in mind: (i) what happens when both are small? (ii) can the problem of small feature size be overcome to some extent by having a larger sample size and vice versa?

*Response: Thanks for this excellent suggestion. We have now added more simulations to study the interaction between sample size and feature size. Please see the Supplementary Fig. S8. Since small sample size tends to reduce power while small feature size tends to increase false positive rate, with a small sample size and feature size we see less inflation than that at a larger sample size. It seems the problem of small feature size or sample size could not be overcome by increasing the other. We modified the text accordingly.*

9. It will also be interesting to see a plot like Fig1A for the cases where 2dFDR performs worse compared to 1dFDR to better understand where it fails. (May be a supplementary figure?)

*Response: We now included Supplementary Fig. 10 showing the decision boundaries under two unfavorable scenarios for 2dFDR (Fig. S10A: colocation of the confounding and true signals with opposite effect sizes so signals can be revealed only by adjustment; Fig. S10 B: independence between the variable of interest and the confounder). We referenced this figure when discussing these two unfavorable scenarios.*

10. I suggest that the feature sizes of each data set is mentioned under “Evaluation of the detection power on real omics datasets”.

*Response: Thanks for the suggestion. We now included them in the main text for more clarity.*

11. Fig 4A: The legend does not show which curve is which.

*Response: Thanks for catching it. We now added the labels to the figure.*

12. Line 178: Do the authors have any insight on why the power increase is more dramatic if the analysis is confined to the polar metabolites?

*Response: Since the number of polar metabolites is only 300, it could be subject to FDR inflation as we have observed at m = 100. We repeated the simulation with m= 300 and found there was indeed some inflation. Therefore, to be conservative, we now removed this panel. This is also the concern of the other reviewer.*

13. Line 196: “(adjusted) p-value” should be defined somewhere in the main manuscript. Line 361 can be more clear on this.

*Response: Thanks. We changed to “confounder-adjusted”.*

14. Lines 215-217: “The power improvement was more dramatic at smaller sample sizes”. It sounds counterintuitive since we saw earlier that 2dFDR did not perform very well when the sample size was very small. In fact, the power improvement here is most dramatic for moderate sample sizes such as 60, 80. Please rephrase these sentences.

*Response: Thanks for pointing it out. This is also the comment from the other reviewer. We now rephrased these sentences as suggested and made the statement more accurate. Please see the modified text in red.*

15. Lines 230-231: “performs well under small sample sizes and feature sizes” – More careful phrasing should be used since it does not perform as well when sample size and/or feature size becomes very small.

*Response: We now rephrased it into “performs well at moderate sample and feature sizes.”*

16. Lines 237-239: One wonders how much the performance may suffer if indeed we apply 2dFDR for a data with very high density of confounding. A short simulation study might be useful.

*Response: We now conducted new simulations with 50% features being affected by the confounder. The results are presented in Supplementary Fig. S1. We can see our method is slightly less powerful than 1dFDR-A in most settings. In such scenarios, adjusting the confounders for all features seems to be a good strategy.*

17. Line 364: Averaging over 100 simulations may be too small. We recommend 1000 simulations (indicated in the figure legend), at least for showing the FDR control. Although the confidence intervals seemed to be narrow enough in most situations, it is not the case for some figures, e.g. Fig S2.

*Response: Thanks. We now used 1000 simulations for settings with wider confidence intervals (Supplementary Fig. S7&8). Now the confidence intervals are smaller.*

18. Fig 2B compared to other figures: For consistency, please include the percentage points for other figures too. This looks especially strange as the first bar charts in the same figure do not have the percentage points.

*Response: We have added the percentage improvement for all relevant figures.*

19. Fig S1: “or 50% (“CoLoc”) of the true signals colocate with the confounding signals”. This is not fully accurate for all cases since the simulation procedure is based on the 50% of the smaller proportion (lines 343-344).

*Response: Thanks for pointing this out. We now changed the text.*

20. Fig S1: “Although in theory the power of 2dFDR dominates 1dFDR-A, the numerical precision of finding the optimal cutoff values on the two dimensions could affect the power slightly.” – I suspect that there could be more to this beyond the numerical precision. The power difference is more than ‘slight’ (up to approximately 8 percentage points in some cases). Can it be the case that the asymptotics do not apply well under certain situations?

*Response: Yes, the reviewer is again correct. We have done more investigation by increasing the density of the search grid, but we still see power loss. Thus, it should be the case the asymptotics do not apply well under these situations. We now removed the statement in the figure legend.*

21. Line 152: “FDR was controlled near the target level” – It seems like the FDR was inflated by as much as 60% compared to the target level in some cases. However, it can be argued that the FDR is inflated for the 1dFDR methods for these cases too, and the 2dFDR method doesn’t do any worse compared to 1dFDR.

*Response: Thanks for being so sharp. We have detected an error in the simulation code, where the added error for each sample was the same (so the correlation is 1 instead of 0.6). After correction, the FDR inflation is not as serious. Really sorry for not being extremely careful when conducting the simulation.*

22. Some notes on the computation time should be included somewhere in the manuscript.

*Response: This is also the comment from the other reviewer. We now provided a supplementary figure showing the computation time at various sample sizes and feature sizes (Supplementary Fig. S9). We also added a corresponding paragraph in the simulation results.*

**Second round of review**

**Reviewer 2**

Regarding Reviewer 1s’ concerns:

No further comment.

Regarding Reviewer 2’s concerns:

The author’s response to most of my comments are satisfactory. However, I have some follow up comments.

1.	(a) In line 33, the authors say “Confounding arises when the relationship between the primary variable and the omics feature is distorted by some other variable (confounder) due to its association with both.” It is unclear why then they define confounding level based on only the association between the confounder and the covariate of interest: “We refer the correlation between the variable of interest and the confounder as the “confounding level” ”. Please provide more discussion on this. Why the association between the outcome and the confounder was not considered at all when talking about confounding level?

(b) How does this discussion then relate to Figure S10A? What was the “confounding level” used for that simulation?

(c) In general, it seems now that most of the gain from the 2dFDR method comes from the situations of no colocation of the effect of x and z. Most simulations are done such that the locations of effects of z and x are independent, which actually implies a very small probability of colocation (especially for low confounding densities when the 2dFDR method seems to have most improved power). The only time the authors studied higher colocation, the improvement of the proposed method seemed to be lacking. This appears to be a big drawback since it’s not actually “confounding” (see (a) above) when there is no colocation. What are the authors’ thought on this?

(c) Lines 87-89 also indicate association of the covariate of interest with outcome and confounder, which seems to be not accurate given how Z^U is calculated.

2.	(a) Line 279-281: “Fortunately, the confounding level can be known before the analysis. We thus recommend running 2dFDR only when there is significant confounding.” – It is not clear how to make this decision in practice. For real data, the “confounding level” will not be constant across features. Please advise how the user should make the decision.

(b) A similar comment was made in the response to reviewer 2 comment #16, which was not discussed in the manuscript. Please include it and also explain more on how the user should make a decision.

3.	Please use more conservative language in some of the figure captions and in the discussion of these figures. For example, in figure S6 : “2dFDR is still much more powerful than 1dFDR-A when the density of the confounding signals is low or the confounding level is high. However, as the confounding signals become denser, the power improvement decreases and 2dFDR is slightly less powerful than 1dFDR-A when the confounding level is low.”

This is not fully accurate. Similar claims are made in some of the other supplementary figure captions.

4.	Some figure captions (e.g. Figure 2) still use “confounding strength” instead of “confounding level”. Please check the figure captions carefully. Figure 1 (B and C) panel headers should also use “confounding level” instead of “c

**Authors Response**

**Point-by-point responses to the reviewers’ comments:**

We thank the reviewer for the constructive and helpful comments, which gives us another chance to improve the clarity of our manuscript.

Reviewer 2:

The author’s response to most of my comments are satisfactory. However, I have some follow up comments.

1.	(a) In line 33, the authors say “Confounding arises when the relationship between the primary variable and the omics feature is distorted by some other variable (confounder) due to its association with both.” It is unclear why then they define confounding level based on **only the association between the confounder and the covariate of interest**: “We refer the correlation between the variable of interest and the confounder as the “confounding level” . Please provide more discussion on this. Why the association between the outcome and the confounder was not considered at all when talking about confounding level?

**Response:** The reviewer is absolutely correct. Confounding consists of two parts: (1) the association between the variable of interest X and the potential confounder Z (cor(X, Z)), and (2) the association between the outcome Yj (j=1,...,m) and the confounder Z (cor(Yj, Z)). In the previous revision, we inaccurately referred “cor(X, Z)” as “ the confounding level”, which causes the confusion. In the new revision, we changed the term “confounding level” to “cor(X, Z)” throughout the texts and the figure labels. In the simulation, we varied cor(X, Z) as well as the density and strength of cor(Yj, Z) to create different degrees of confounding. Our method is most powerful when cor(X, Z) is high, cor(Yj, Z) is sparse and cor(Yj, Z)&cor(Yj, X) do not always co-locate. In such situation, Z is not a true confounder for most features, therefore, adjusting Z will be redundant and will hurt the statistical power accordingly. However, there are no rigorous methods out there, to our best knowledge, to decide which features we should adjust or not. The dominant approach in the real world uses the conservative method of adjusting Z for all features. Our method circumvents the difficulty in determining which features to adjust by employing a two-step procedure. We acknowledge that there could be many examples, which fall out of our method’s favorable parameter space. But we also believe that there could be a plenty of examples, where our method is useful.

(b) How does this discussion then relate to Figure S10A? What was the “confounding level” used for that simulation?

**Response:** Figure S10A simulated an unfavorable condition for our method, where the true and confounding signals co-locate with opposite directions. The true signals can be only revealed after we adjust Z. Therefore, the unadjusted statistic is not informative. In Figure S10A, we simulated a moderate cor(X, Z) (++), 10% true and confounding signals with moderate strength. We added more discussions and made the parameter setting clear in the figure legend.

(c) In general, it seems now that most of the gain from the 2dFDR method comes from the situations of no colocation of the effect of x and z. Most simulations are done such that the locations of effects of z and x are independent, which actually implies a very small probability of

colocation (especially for low confounding densities when the 2dFDR method seems to have most improved power). The only time the authors studied higher colocation, the improvement of the proposed method seemed to be lacking. This appears to be a big drawback since it’s not actually “confounding” (see (a) above) when there is no colocation. What are the authors’ thought on this?

**Response:** The reviewer is right that our method is most powerful when Z are not true confounders for most features, so adjustment is not necessary. When Z are true confounders for a large number of features, our method could have some power loss compared to the standard procedure, which adjusts Z for all features. However, in practice, it is very difficult to determine whether Z is a true confounder or not, and what we could observe directly is the correlation between X and Z. Our method aims to rescue power for settings where the traditional procedure is powerless and tries not to lose much power in other settings. We believe that there could be many real applications, where the highly correlated X and Z affect a sparse and possibly different set of features so that our method could be very useful.

(c) Lines 87-89 also indicate association of the covariate of interest with outcome **and confounder**, which seems to be not accurate given how Z^U is calculated.

**Response:** If a feature is not associated with either the outcome or the confounder, we will expect ZU to be small. Thus, if we assume that the signals are sparse, we could filter out a large number of irrelevant features based on Z^U^. We acknowledge that there does exist some situation (see discussions and Figure S10A), where filtering could erroneously exclude true signals.

2.	(a) Line 279-281: “Fortunately, the confounding level can be known before the analysis. We thus recommend running 2dFDR only when there is significant confounding.” – It is not clear how to make this decision in practice. For real data, the “confounding level” will not be constant across features. Please advise how the user should make the decision.

**Response:** This is again due to the misunderstanding of the term “confounding level”. Here we

mean the correlation between the variable of interest and the confounder. We now revised the text to be clearer about that.

(b) A similar comment was made in the response to reviewer 2 comment #16, which was not discussed in the manuscript. Please include it and also explain more on how the user should make a decision.

**Response:** Thanks for pointing this out. We now expanded the discussion and added two potential diagnostic approaches to identify dense confounder signal scenarios. See below for the added discussion:

“However, there could be situations where the assumption is violated. For example, strong batch effects could possibly affect a large number of omics features. In such a case, 2dFDR has a limited power advantage or could be less powerful than the traditional procedure (**Supplementary Fig. S1)**. One diagnostic approach is to calculate the percentage of the genomic variance explained (R^2^) by the variable of interest and the confounder, respectively, using multivariate methods such as PERMANOVA[27]. If the R^2^ of the confounder is substantially larger than that of the variable of interest, it indicates that the confounder signals may be very dense. Another approach is to study the distribution of the p-values of the confounder from the

adjusted analysis. If we see a spike on the left end of the p-value distribution, it also suggests dense confounder signals.”

3. Please use more conservative language in some of the figure captions and in the discussion of these figures. For example, in figure S6 : “2dFDR is still much more powerful than 1dFDR-A when the density of the confounding signals is low or the confounding level is high. However, as the confounding signals become denser, the power improvement decreases and 2dFDR is slightly less powerful than 1dFDR-A when the confounding level is low.”

This is not fully accurate. Similar claims are made in some of the other supplementary figure captions.

**Response:** Thanks for this nice suggestion. We now revised the texts and stated in a more conservative way.

4. Some figure captions (e.g. Figure 2) still use “confounding strength” instead of “confounding level”. Please check the figure captions carefully. Figure 1 (B and C) panel headers should also use “confounding level” instead of “confounding”.

**Response:** Thanks! We now changed the captions and labels accordingly.
